# Efficacy, Safety, and Tolerability of Treatments for Systemic Sclerosis-Related Interstitial Lung Disease: A Systematic Review and Network Meta-Analysis

**DOI:** 10.3390/jcm9082560

**Published:** 2020-08-07

**Authors:** Gian Luca Erre, Marco Sebastiani, Maria Antonietta Fenu, Angelo Zinellu, Alberto Floris, Lorenzo Cavagna, Elisabetta Renzoni, Andreina Manfredi, Giuseppe Passiu, Richard John Woodman, Arduino Aleksander Mangoni

**Affiliations:** 1 Dipartimento di Scienze Mediche, Chirurgiche e Sperimentali, Università degli Studi di Sassari, 07100 Sassari, Italy; gpassiu@uniss.it; 2 Dipartimento di Specialità Mediche, Azienda Ospedaliero-Universitaria di Sassari, 07100 Sassari, Italy; maria26fe@gmail.com; 3 Chair and Rheumatology Unit, University of Modena and Reggio Emilia, Azienda Ospedaliero-Universitaria Policlinico di Modena, 41121 Modena, Italy; marco.sebastiani@unimore.it (M.S.); andreina.manfredi@gmail.com (A.M.); 4 Dipartimento di Scienze Biomediche, Università degli Studi di Sassari, 07100 Sassari, Italy; azinellu@uniss.it; 5 Azienda Ospedaliero-Universitaria di Cagliari, 09042 Monserrato, Italy; alberto.floris@unica.it; 6 Division of Rheumatology, University and IRCCS Policlinico S. Matteo Foundation, 27100 Pavia, Italy; lorenzo.cavagna@unipv.it; 7 Interstitial Lung Disease Unit, Royal Brompton Hospital, London SW3 6NP, UK; e.renzoni@imperial.ac.uk; 8 Flinders Centre for Epidemiology and Biostatistics, College of Medicine and Public Health, Flinders University and Flinders Medical Centre, Adelaide 5001, Australia; richard.woodman@flinders.edu.au; 9 Discipline of Clinical Pharmacology, College of Medicine and Public Health, Flinders University and Flinders Medical Centre, Adelaide 5001, Australia; arduino.mangoni@flinders.edu.au; 10 Medizinische Fakultät Carl Gustav Carus, Technische Universität Dresden, 01307 Dresden, Germany

**Keywords:** interstitial lung disease, systemic sclerosis, network meta-analysis, randomized controlled trials, cyclophosphamide, mycophenolate mofetil, rituximab, nintedanib, hematopoietic stem-cell transplantation, pomalidomide

## Abstract

Background: There is a paucity of head-to-head comparisons of the efficacy and harms of pharmacological treatments for systemic sclerosis-related interstitial lung disease (SSc-ILD). Methods: We conducted a network meta-analysis (NMA) in order to compare the effects of different treatments with the placebo on change in forced vital capacity (FVC), change in diffusion lung capacity for CO (DLCO), serious adverse events (SAEs), discontinuation for adverse events and mortality in SSc-ILD. Standardized mean difference (SMD) and log odds ratio were estimated using NMA with fixed effects. Results: Nine randomized clinical trials (926 participants) comparing eight interventions and the placebo for an average follow-up of one year were included. Compared to the placebo, only rituximab significantly reduced FVC decline (SMD (95% CI) = 1.00 (0.39 to 1.61)). Suitable data on FVC outcome for nintedanib were not available for the analysis. No treatments influenced DLCO. Safety and mortality were also not different across treatments and the placebo, although there were few reported events. Cyclophosphamide and pomalidomide were less tolerated than the placebo, mycophenolate, and nintedanib. Conclusion: Only rituximab significantly reduced lung function decline compared to the placebo. However, direct head-to-head comparison studies are required to confirm these findings and to better determine the safety profile of various treatments.

## 1. Introduction

Systemic sclerosis (SSc) is an immune-mediated connective tissue disease (CTD) characterized by extensive fibrosis of the skin and internal organs and vasculopathy [1]. Interstitial lung disease (SSc-ILD) affects a considerable proportion of SSc patients [2] early in the course of disease and especially in the presence of diffuse skin involvement (diffuse SSc, dcSSc) [3]. The presence of ILD predicts poor outcome, accounting for about 20% of overall mortality in SSc patients [4]. 

Patients with SSc-ILD experience exertional dyspnoea, non-productive cough, and fatigue, with interstitial abnormalities on high-resolution computerized tomography (HRCT) detected in as many as 90%. Pulmonary function tests (PFTs) typically demonstrate a restrictive pattern, with reduced forced vital capacity (FVC) and diffusing capacity of the lung for carbon monoxide (DLCO).

SSc-ILD may remain substantially stable over time or show a slow progression accompanied by decline in pulmonary function test parameters and worsening of symptoms. Although infrequently, rapid loss of lung function can occur in the context of acute exacerbation [5]. 

Treatment of SSc-ILD is still challenging due to scarcity of available treatments. Immunosuppressors are considered the mainstay of treatment for SSc-ILD, with cyclophosphamide and mycophenolate mofetil (mycophenolate) being the most widely used agents based on the results of two large randomized clinical trials (RCTs) that assessed the effect of cyclophosphamide against the placebo (The Scleroderma Lung Study-I, SLS-I) and cyclophosphamide against mycophenolate (The Scleroderma Lung Study-II, SLS-II) on lung function decline [6,7,8]. Moreover, observational studies and two small RCTs reported efficacy of rituximab in decreasing the rate of SSc-ILD progression, suggesting its use as rescue therapy in patients progressing despite treatment with cyclophosphamide and mycophenolate [9]. Recently, nintedanib, a tyrosine kinase inhibitor, was licensed in USA and Europe to treat adult patients with SSc-ILD based on the results of the SENSCIS trial, a phase III, double-blind, placebo-controlled trial that evaluated the effect of nintedanib against the placebo on the annual rate of FVC decline [10]. 

However, head-to-head direct comparisons of different drugs in high quality studies that may inform evidence-based treatment regimens in SSc-ILD are still lacking. A recently published network meta-analysis (NMA) reported the potential benefit of cyclophosphamide plus azathioprine and mycophenolate compared to other treatment interventions in reducing the lung functional decline [11]. However, this NMA did not fully analyze the available evidence as it focused on selected immunosuppressive drugs. More importantly, the inclusion of both RCT and non-randomized observational trials may have affected the quality of the evidence generated.

Given the ongoing clinical need to establish evidence-based recommendations regarding the treatment of SS-ILD, we performed a multiple treatment comparison based on a NMA that considered both direct and indirect comparisons of all interventions tested in RCTs of patients with SSc-ILD. We focused on clinically relevant measures of efficacy and safety such as absolute change in FVC % of predicted and DLCO % of predicted, number of patients with serious adverse events (SAEs), number of patients discontinuing treatment due to adverse events (AEs) and mortality.

## 2. Experimental Section

This study was reported according to the Preferred Reporting Items for Systematic Reviews and Meta Analyses (PRISMA) statement for reporting of systematic reviews incorporating network meta-analyses of health care interventions [12]. 

### 2.1. Eligibility Criteria

The PICOS (problem/patient, intervention, comparison, outcome, study design) method was used to screen studies. Selection criteria were as follows:

1. Participants: females and males aged ≥18 years. Diagnosis of SSc-ILD according to validated criteria.
2. Interventions and comparisons: various pharmacological interventions, alone or in combination, and placebo
3. Outcome measures: (a) mean change in “FVC % of predicted” from baseline to 12 months; (b) mean change in “DLCO % of predicted” from baseline to 12 months; (c) number of patients with SAEs; (d) number of patients discontinuing treatment due to AEs; (e) mortality. Outcome measures collected at different follow-up timepoints were pooled at annual intervals, plus or minus six months, with the primary analysis covering the first 12 months.
4. Study design: RCTs.

### 2.2. Exclusion Criteria 

We excluded RCTs not specifically enrolling patients with a definite diagnosis of SSc-ILD made according to PFTs and HRCT criteria. Quasi-randomized studies were also excluded. 

### 2.3. Information Sources and Searches

The following electronic databases were searched from inception to 14 April 2020: (1) Scopus; (2) EU Clinical Trials Registry EudraCT; (3) ClinicalTrials.gov; (4) Web of Science (Web of Science Core Collection, Biological Abstracts, KCI, Korean Journal Database, MEDLINE^®^, Russian Science Citation Index, and SciELO Citation Index). The search strategy in Scopus was as follows: (((((KEY (systemic sclerosis)) or (KEY (scleroderma)) or (KEY (SSc-ILD))) and not ((KEY (multiple sclerosis)) or (KEY (tuberous sclerosis)))) and ((KEY (pulmonary fibrosis)) or (KEY (interstitial lung disease)) or (KEY (pneumonia)) or (KEY (lung)))) and ((TITLE-ABS-KEY (randomized)) or (TITLE-ABS-KEY(randomised)) or (TITLE-ABS-KEY(trial)) or (TITLE-ABS-KEY(controlled)) or (TITLE-ABS-KEY (placebo)) or (TITLE-ABS-KEY (versus)))) and not ((TITLE-ABS-KEY (review)) or (TITLE-ABS-KEY (open-label)) or (TITLE-ABS-KEY (retrospective))) and not index (medline). The search strategies for different databases are described in the Supplementary material 11, page 62. There was no restriction to English language. The reference list of individual papers was also reviewed to identify additional studies. 

### 2.4. Data Extraction and Summary Measures

Two reviewers (G.L.E. and A.Z.) independently reviewed the literature and extracted the following data: title and reference details (first author, year), study population characteristics (age, proportion of females, disease duration, proportion of patients with dcSSc, and baseline values of FVC % of predicted and DLCO % of predicted), inclusion/exclusion criteria, outcomes measures, sponsor, type of interventions, and outcome data for pooling. 

Hedges’ standardized mean difference (SMD) of the change between baseline and follow-up visit (change score) and the log odds-ratio (OR) with 95% confidence intervals (CIs) were used to describe the effects. Although the changes in FVC % of predicted and DLCO % of predicted were expressed using the same unit of measurement in each RCT, we used SMD as a measure of effect size, instead of the mean difference, because it is more easily interpretable by clinicians.

When not available, mean change scores and/or standard deviations of the mean change scores were calculated according to recommendations provided in the Cochrane Handbook for Systematic Reviews of Interventions [13]. In particular, standard deviations of change scores were calculated using an imputed correlation factor (Corr.) of 0.8, together with the following formula [13]:

SD_E, change_ = √SD^2^_E, baseline_ + SD^2^_E, final_ − (2 × Corr × SD_E, baseline_ × SD_E, final_).

### 2.5. Risk of Bias (RoB)

G.L.E. (as content expert) and A.Z. (as methodologist) independently assessed the risk of bias (RoB) of each study for the following domains: sequence generation, allocation concealment, blinding of participants and personnel, blinding of pulmonary function technologists, selective reporting, attrition bias, and other RoB. In case of disagreement, the final rating of RoB was reached by consensus with involvement of a third reviewer (A.A.M., as methodologist). According to Cipriani et al. [14,15], the overall RoB of each study was assessed as follows: (1) low RoB studies: none of the domains was rated as high risk and three or less were rated as unclear risk; (2) moderate RoB studies: one domain was rated as high risk or none was rated as high risk but four or more were rated as unclear risk; (3) high RoB studies: all other combinations.

### 2.6. Assessment of Reporting Bias and Sensitivity Analysis

Given that only one study per comparison group was available, data were insufficient to evaluate reporting bias by funnel plot. We performed sensitivity analysis according to different values of correlation factor (0.8, 0.7, and 0.5) and excluded studies with length of follow-up <12 months. 

### 2.7. GRADE Quality Assessment 

We assessed the level of certainty of evidence contributing to estimates for each outcome included in the NMA using the GRADE framework, rating the quality of a body of evidence on the basis of the study limitations, imprecision, inconsistency, and indirectness [16]. We started rating as high the certainty of evidence of the estimate in each network estimate, and downgraded according to the assessments of these five domains. We downgraded by 1 point in the presence of serious concerns and by 2 points in the presence of very serious concerns [17]. 

### 2.8. Network Meta-Analysis

We used the multivariate meta-analysis model where different treatment comparisons are treated as different outcomes [18,19]. We used the “network” suite of commands available in STATA (StataCorp, 2011; Stata Statistical Software: Release 14. College Station, TX, USA [19,20]. Given the absence of heterogeneity (only one RCT for every comparison included in the NMA), a fixed–effects model with the inverse variance method was used [19]. Indirect comparisons between active treatments were performed using the placebo as a common comparator. Consistency between direct and indirect effect estimates was assessed by local and global approaches. We evaluated local consistency by the inconsistency factor (IF) in closed loops and node splitting method [21]. IF is the logarithm of risk odds ratio (ROR) between direct and indirect estimates in a given closed loop. An IF higher than 2 indicates high inconsistency in a closed loop. Loops in which the lower confidence interval limit of the IF reaches the zero line are considered to present no statistically significant inconsistency [20]. To check the assumption of consistency in the entire network, we calculated the Chi^2^ test of the “design-by-treatment” model [22]. Treatments were then ranked according to surface under the cumulative ranking curves (SUCRAs). SUCRA transforms the cumulative probabilities of being the best treatment into a single value between 0% and 100%, where the larger the SUCRA value, the better the ranking of the treatment with a value of 100%, representing a high probability of being the best ranking treatment. 

## 3. Results

### 3.1. Search Results

**Figure 1:**
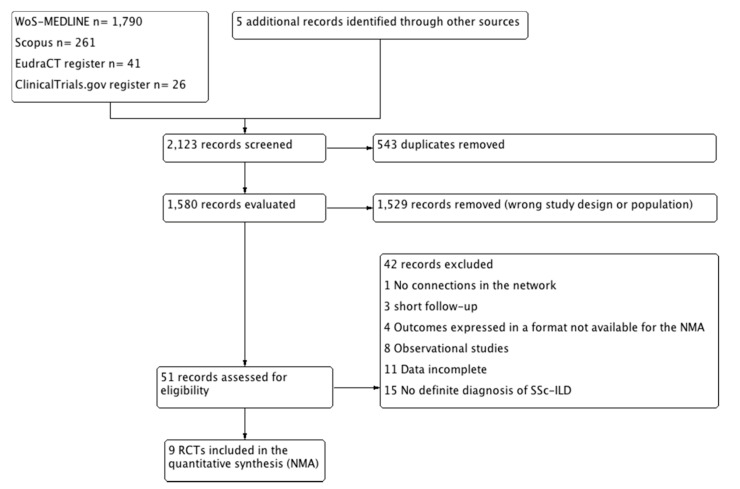
Flowchart. SSc-ILD, systemic sclerosis-related interstitial lung disease; RCTs, randomized controlled trials; NMA, network meta-analysis.

A total of 2118 titles were initially identified and combined with five studies that were found after manual search of the reference lists. Of these 2123 references, 1529 were considered ineligible after duplicate removal and review of titles and abstracts, leaving 51 records for full text review (see Figure 1).

### 3.2. Included Studies

Nine RCTs (SLS-I, 2006 [8]; SLS-II, 2016 [7]; Domiciano, D.S., 2011 [23]; Hoyles, R.K., 2006 [24]; Naidu, G.S.R.S.N.K., 2020 [25]; SENSCIS, 2019 [10]; Sircar, G., 2018 [26]; Acharya, N., 2019 [27]; Hsu, V.M., 2018 [28]) were considered eligible and were included in the final quantitative analysis (see Supplementary material 1.1, page 5). These studies were conducted in a total of 926 participants, comparing 8 interventions (cyclophosphamide, mycophenolate, cyclophosphamide plus high dose prednisone (CYCPRED), cyclophosphamide followed by azathioprine (CYCAZA), rituximab, pirfenidone, nintedanib, and pomalidomide), and placebo for an average follow-up of 1 year. 

Mean age ranged from 34.6 to 54.6 years. As expected, patients were more frequently females (see Supplementary material 1.2, page 6). SSc-ILD was diagnosed using HRCT criteria in 5 RCTs (SLS-II, 2016 [7]; Naidu G.S.R.S.N.K., 2020 [25]; SENSCIS, 2019 [10]; Acharya, N., 2019 [27]; Hsu, V.M., 2018 [28]), HRCT plus biopsy, PFTs or biopsy in 3 RCTs (SLS-I, 2006 [8]; Hoyles, R.K., 2006 [24]; Sircar, G., 2018 [26]), and biopsy only in the remaining RCT (Domiciano, D.S., 2011 [23]). Domiciano, D.S., 2011 [23] enrolled only SSc-ILD patients with biopsy-proven nonspecific interstitial pneumonia (NSIP), while Naidu, G.S.R.S.N.K., 2020 [25], Sircar, G., 2018 [26], and Acharya, N., 2019 [27] enrolled patients with both NSIP and usual interstitial pneumonia (UIP). SSc-ILD pattern was not specified in the remaining RCTs (see Supplementary material 1.2, page 6).

**Figure 2:**
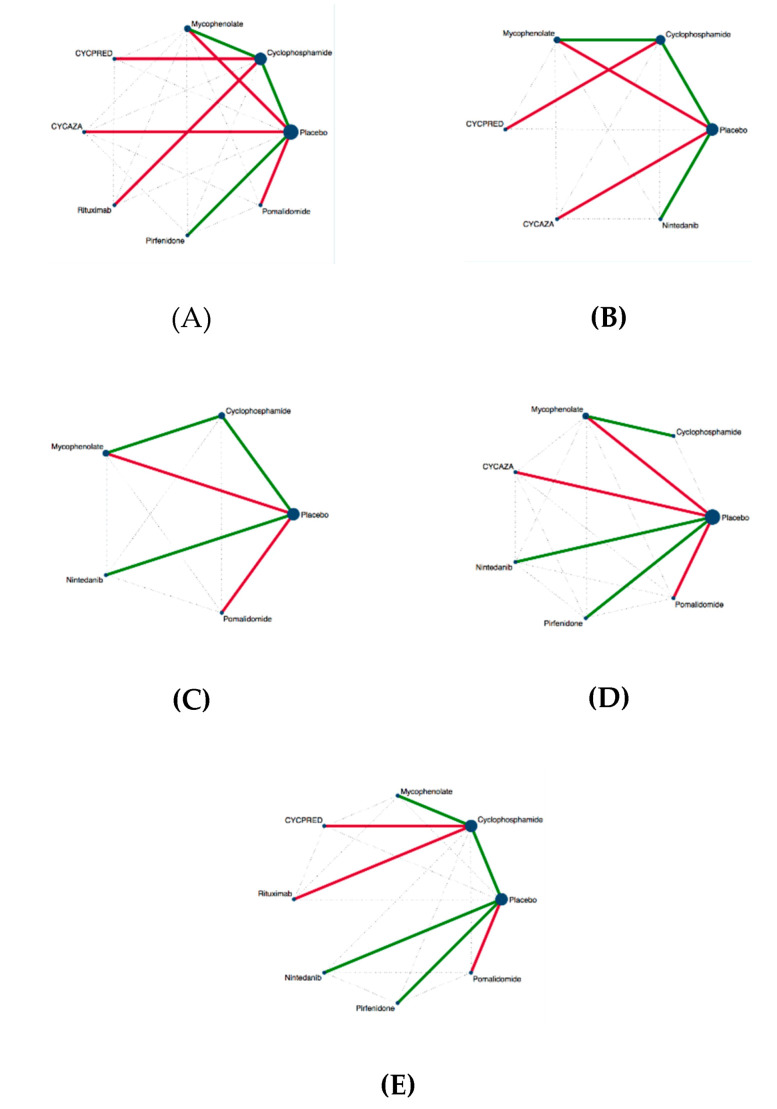
Network structures. (**A**), change in FVC % predicted network; (**B**), change in DLCO % predicted network; (**C**), number of patients with SAEs network; (**D**), number of patients discontinuing treatment for AEs network; (**E**), deaths network; Edges are colored according to the level of RoB of trials: green low RoB; red high RoB. Nodes were sized according to the number of participants. Dotted lines represent primary indirect comparisons.

Figure 2 describes the networks of eligible comparisons for outcomes included in the NMA. Cyclophosphamide vs. placebo [8] was the direct comparison contributing the most to all networks. The relative contribution of direct and indirect comparisons and overall contributions are reported as netweight plots in the Supplementary materials 5.1–5.5, pages 34–38.

### 3.3. Excluded Studies 

We excluded 42 studies (Abou-Raya, A., 2013 [29]; Allanore, Y., 2018 [30]; Allanore, Y., 2019 [31]; ASSET, 2019 [32]; ASSIST, 2011 [33]; Boonstra, M., 2017 [34]; Chakravarty, E.F., 2015 [35]; Daoussis, D., 2010 [36]; Daoussis, D., 2012 [37]; Daoussis, D., 2017 [38]; Denton, C., 2007 [39]; EDITA, 2019 [40]; FaSScinate, 2016 [41]; FocuSSced, 2020 [42]; Gordon, J.K., 2018 [43]; Gruber, B.L., 1991 [44]; Guillevin, L., 1982 [45]; Guo, H.M., 2008 [46]; Henes, J., 2020 [47]; Herrick, A.L., 2017 [48]; Hoffmann-Vold, A.M., 2019 [49]; Khanna, D., 2009 [50]; Khanna, D., 2019 [50]; Mehrabi, S., 2019 [51]; Nadashkevich, O., 2008 [52]; NCT02283762 [53]; NCT02465437 [54]; NCT02745145 [55]; Pakas, J., 2002 [56]; Panopoulos, S.T., 2013 [57]; Poormoghim, H., 2013 [58]; Pope, J.E., 2001 [59]; Prey, S., 2012 [60]; Quillinan, N.P., 2014 [61]; Schiopu, E., 2016 [62]; Sclero XIII, 2019 [63]; Seibold, J.R., 2000 [64]; Seibold, J.R., 2010 [65]; Su, T.I.K., 2009 [66]; Sullivan, A., 2018 [67]; van den Hoogen, F.H.J., 1996 [68]; van Laar, J.M., 2014 [69]). Reasons for exclusion are described in the Supplementary material 2.1, pages 25–26.

### 3.4. Quality Assessment 

Four trials were scored with a low RoB (Tashkin, D.P., 2006 [8]; Tashkin, D.P., 2016 [7]; SENSCIS, 2019 [10]; Acharya, N., 2019 [27]), whereas the remaining five trials were scored with a high RoB (Domiciano, D.S., 2011 [23]; Hoyles, R.K., 2006 [24]; Naidu, G.S.RS.N.K., 2020 [25]; Sircar, G., 2018 [26]; Hsu, V.M., 2018 [28]). No trial was scored with an intermediate RoB (see Supplementary Material 3.1–3.3, pages 30–31). 

### 3.5. Inconsistency in NMA

A total of three closed loops were found in the efficacy and safety networks, including cyclophosphamide, mycophenolate, and placebo. The IF values in these loops were <1, suggesting consistence in direct and indirect estimates (see Supplementary materials 6.1–6.3, pages 40–42). The node splitting approach demonstrated no significant differences between direct and indirect estimates. Similarly, results of design by treatment tests were not significant for presence of inconsistence in the entire networks (see Supplementary materials 6.1–6.3, pages 40–42). The tolerability and mortality network did not include closed loops.

### 3.6. Similarities Between Studies

According to the selection criteria, the period of observation was one year in six trials and six months in the remaining three trials (Naidu, G.S.R.S.N.K., 2020 [25]; Sircar, G., 2018 [26]; Acharya, N., 2019 [27]). 

Baseline values of efficacy outcomes were similar across trials with Naidu, G.S.R.S.K.N., 2011 [25], Domiciano, D.S., 2011 [23], and Sircar, G., 2018 [26] enrolling patients with milder disease. All studies enrolled subjects with disease duration <5 years, with the exception of Domiciano, D.S., 2011 [23] (mean disease duration 5.8/6.0 years). In most trials, low-dose steroids were administered as background therapy, and in four, the concomitant use of immunosuppressors was allowed. Based on visual inspection, patient characteristics were considered similar not only across the selected RCTs but also to those of SSc-ILD patients treated in routine clinical practice. Therefore, barring differences in the length of follow-up, we did not downgrade for intransitivity. The characteristics of RCTs included in the NMA are described in the Supplementary material 1.2, page 6.

### 3.7. Efficacy Analysis

**Figure 3:**
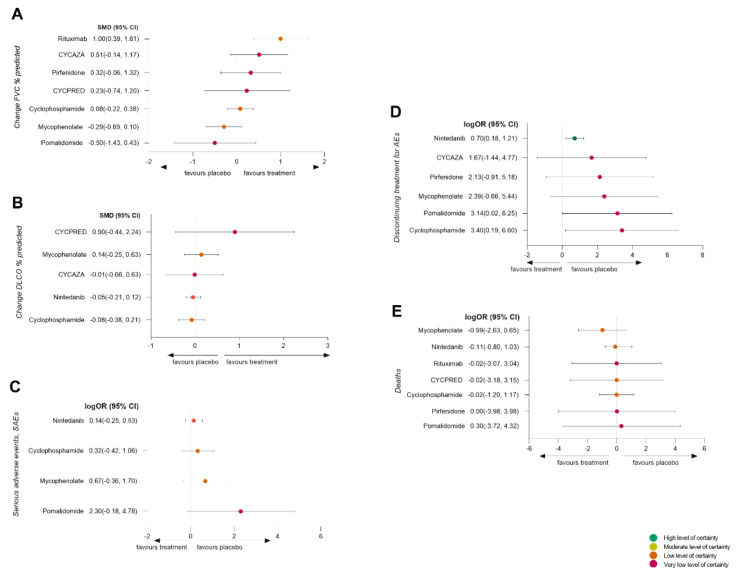
Forest plots. Forest plots of network meta-analysis of all trials: (**A**), change in forced vital capacity % of predicted (FVC %); (**B**), change in diffusion lung capacity for CO % of predicted (DLCO %); (**C**), number of patients with serious adverse events (SAEs); (**D**), number of patients discontinuing treatment for adverse events (AEs); (**E**), deaths. Treatments were compared with the placebo. SMD, standardized mean difference; log OR, odds ratio. CI, confidence interval.

Eight RCTs (for a total of 451 patients) reporting change in FVC % of predicted and seven RCTs (for a total of 813 patients) reporting change in DLCO % of predicted were included in the analysis. Rituximab was the only treatment that was significantly more effective than the placebo in reducing decline of FVC % of predicted (SMD (95% CI) = 1.00 (0.39 to 1.61), low certainty of evidence) (see Figure 3A; Supplementary materials 4.1, page 32, and 7.1, pages 42–44). Efficacy of the remaining treatments, including cyclophosphamide and mycophenolate, on reducing decline of FVC % of predicted was not significantly different from the placebo (SMD (95% CI), ranging from 0.51 (−0.14 to 1.17) for CYCAZA to −0.50 (−1.43 to 0.43) for pomalidomide; certainty of evidence low to very low) (see Figure 3A; Supplementary materials 4.1, page 32, and 7.1, pages 42–44). In the SENSCIS, 2019 [10] trial, the efficacy of nintedanib in reducing the decline of FCV % of predicted was significant with respect to the placebo. However, the outcome was expressed as an annual decline rate, and therefore, study data were not suitable for the statistical analysis in this NMA.

**Figure 4:**
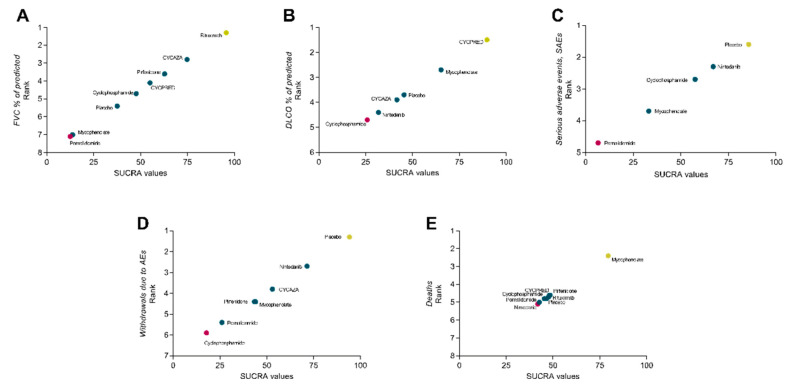
Surface under the cumulative ranking curves (SUCRAs) vs. rank. (**A**), change in forced vital capacity % of predicted (FVC %); (**B**), change in diffusion lung capacity for CO % of predicted (DLCO %); (**C**), number of patients with serious adverse events (SAEs); (**D**), number of patients discontinuing treatment for adverse events (AEs); (**E**), deaths. Treatments have been ranked according to SUCRAs. For example, in (**A**), rituximab is the treatment with the highest probability of being the best (RANK 1) having the highest SUCRA (95.7). (**C**–**E**), treatment with the best rank are those with better tolerability, safety, and lower number of deaths, respectively.

Rituximab ranked as the best treatment for reducing the decline of FVC % of predicted (SUCRA = 95.7), followed by CYCAZA (SUCRA = 74.8) and pirfenidone (SUCRA = 62.8) (See Figure 4A and Supplementary material 8.1, page 53). However, the certainty of the evidence for the ranking was low. 

None of the assessed treatments were significantly more effective than the placebo in reducing the decline of DLCO % of predicted (SMD (95% CI), ranging from 0.90 (−0.44 to 2.24) for CYCPRED to −0.08 (−0.38 to 0.21) for cyclophosphamide; certainty of evidence was low to very low) (see Figure 3B and Supplementary material 8.2, page 55). 

CYCPRED was rated as the best treatment (SUCRA = 89.8), followed by mycophenolate (SUCRA = 65.3), but the certainty of the evidence for the ranking was low (see Figure 4B and Supplementary material 8.2, page 54). 

### 3.8. Safety Analysis 

The method of reporting the type and rate of AEs varied widely between RCTs. Only five RCTs (for a total of 777 patients) reporting the number of patients with SAEs were included in the NMA. 

There was no significant difference between treatments and the placebo in terms of number of patients experiencing SAEs (see Figure 3C and Supplementary material 4.1, page 33), with logOR (95% CI) ranging from 0.14 (−0.25 to 0.53) for nintedanib to 2.30 (−0.18 to 4.78) for pomalidomide. However, low to very low certainty of evidence informed our results for number of patients with SAEs (see Supplementary material 7.3, page 47). Among treatments, nintedanib ranked second as the safest treatment after the placebo (see Figure 4C and Supplementary material 8.3, page 55). However, the grade of confidence in the safety ranking was low.

### 3.9. Tolerability Analysis 

Six RCTs (for a total of 703 patients) reporting tolerability, i.e., the number of patients discontinuing treatment because of the occurrence of AEs, were included in the NMA. 

The use of nintedanib, pomalidomide, and cyclophosphamide was associated with a significantly higher number of patients withdrawing treatments due to AEs compared to the placebo (logOR (95% CI) = 0.70 (0.18 to 1.21), 3.14 (0.02 to 6.25), and 3.40 (0.19 to 6.60), respectively (see Supplementary material 4.4 page 33); level of certainty of evidence was high for nintedanib and low for pomalidomide and cyclophosphamide) (see Supplementary material 7.4, pages 48–50). However, nintedanib ranked second, only after the placebo, for tolerability (SUCRA = 71.5) (see Figure 4D and Supplementary material 8.4, page 56). Again, certainty of evidence for tolerability ranking was low.

### 3.10. Mortality Analysis 

We included in the NMA seven RCTs (for a total of 855 patients) reporting the number of deaths at the longest available follow-up. In the context of a very low number of events, the number of deaths was not significantly different between treatments and the placebo with logOR (95% CI) ranging from -0.99 (−2.63 to 0.65) for mycophenolate to 0.30 (−3.72 to 4.32) for pomalidomide (see Figure 3E; Supplementary file 4.5, page 33). However, low to very low quality evidence informed our results for the number deaths (see Supplementary material 7.5, pages 50–52). Mycophenolate was the treatment with higher SUCRA (SUCRA = 79.5) (see Figure 4E and Supplementary material 8.5, page 57), but the grade of confidence in the ranking was low.

### 3.11. Sensitivity Analysis

There was no significant difference in effect size estimates in the primary analysis of efficacy according to different values of correlation factor (see Supplementary Materials Tables S9.1.1 and 9.1.2, page 58). Removing studies with short length of follow-up (<12 months, n = 3), there was no significant change in the size, direction, and significance of effect estimates for the efficacy of treatments against the placebo (see Supplementary Materials Tables S9.2.1 and 9.2.2, page 58).

## 4. Discussion

In this review and NMA of nine RCTs investigating eight treatments and the placebo in a total of 926 patients with SSc-ILD, only rituximab significantly reduced the decline of FVC % of predicted, but not of DLCO % of predicted, compared with the placebo. The effect of the remaining treatments, including cyclophosphamide and mycophenolate, on lung function was not significantly different from that of the placebo. 

Rituximab is a chimeric (human/mouse) monoclonal antibody targeting CD20-positive B-cell. Beside hematological neoplasms, rituximab-mediated rapid B-cell depletion has proven to be effective in immune-mediated diseases such as rheumatoid arthritis, and granulomatosis with polyangiitis. This has prompted various investigators to study the effect of rituximab on lung function in patients with SSc-ILD. However, as reviewed by Bellan et al. [70], results from open label observational studies were largely contradictory, with some reporting efficacy [36,37,38] while others reported null effects [71,72,73] on lung function. In addition, an analysis of prospectively collected data from the European League Against Rheumatism Scleroderma Trials and Research (EUSTAR) Group showed no significant improvement in lung function in patients receiving rituximab over a mean follow-up period of two years [74]. Similarly, in a small, randomized, double-blind trial, rituximab did not significantly reduce decline of FVC % of predicted over 24 months when compared to the placebo [34].

In our NMA, the use of rituximab was associated with a significant reduction in FVC decline. However, certainty of evidence is low. The effect estimate of rituximab compared with the placebo was in fact indirectly calculated from a small-sized unblinded RCT that compared the effect of rituximab and cyclophosphamide on lung function over six months [26]. Therefore, evidence from larger prospective trials that provide additional directly estimated effects are required to confirm the results of our NMA, suggesting a potential positive effect of rituximab in SSc-ILD. Results from RECITAL [75], a multicenter, prospective, randomized, double-blind, trial comparing rituximab with cyclophosphamide in patients with severe, progressive CTD-ILD, are expected to build on the available evidence regarding efficacy and safety of rituximab in SSc-ILD.

Cyclophosphamide is the most widely used treatment for SSc-ILD worldwide [76], and the only treatment together with hematopoietic stem cell therapy (HSCT) recommended by the European League against Rheumatism (EULAR) for SSc-ILD [6]. This recommendation is based on the results of the SLS-I trial that showed a modest, but significant, improvement in FVC % of predicted at one year of follow-up in patients receiving cyclophosphamide when compared to the placebo with an adjusted (for baseline value) mean absolute difference in FVC at 12 months of 2.53 percent (95% CI, 0.28 to 4.79) [8]. However, this average small difference was probably related to the fact that many patients included in the trial had limited and stable ILD, as suggested by the average stability of the placebo group in the SLS I trial. The benefit of cyclophosphamide was maximal at 18 months and rapidly dissipated after cessation of treatment [77], suggesting the need for ongoing immunosuppression. 

Contrary to what was expected, in our NMA, the SMD in FVC at 12 months between cyclophosphamide and the placebo, although showing a positive trend in favor of cyclophosphamide (SMD = 0.08), did not reach statistical significance (95% CI, −0.22 to 0.38). In this regard, it should be emphasized that the effect size of cyclophosphamide is the result of a multiple comparison between treatment effects of cyclophosphamide and the placebo, resulting from the pooling of studies with different length of follow-up. However, the effect size of cyclophosphamide remained unchanged after sensitivity analysis, which excluded studies with short follow-up. 

The findings of our NMA contrast with the results of two meta-analyses [78,79] reporting a significant, albeit modest, benefit in FVC decline in people with SSc-ILD receiving cyclophosphamide. In this regard, it should be underlined that both meta-analyses used imputed adjusted mean differences for FVC at 12 months, while in our NMA, we imputed unadjusted change scores as calculated from published raw data. Accordingly, in a meta-analysis of RCTs from Nannini et al. [80] based on unadjusted change scores, the use of cyclophosphamide did not result in a statistically meaningful improvement in lung functional decline. 

However, it is conceivable that the inclusion of SSc-ILD patients with different disease severity may have influenced the study results and, therefore, the NMA effect size estimate. Patients with more severe lung disease are, in fact, likely to respond better to cyclophosphamide compared to patients with mild, stable, disease. In a retrospective analysis of the SLS-I trial, the subgroup of patients with reticular infiltrates on more than 50% of the lung volume on baseline HRCT showed a significant cyclophosphamide treatment effect of 4.73% at 12 months and 9.81% at 18 months. By contrast, the use of cyclophosphamide was not effective (−0.58%) in patients with milder extent of lung fibrosis on HRCT [77]. Therefore, the pooled analysis of treatment effects from studies including patients with different extent of lung involvement may well have diluted the effect of cyclophosphamide. Unfortunately, in this review, there were insufficient observations to perform a reliable meta-regression analysis accounting for HRCT fibrotic score (and baseline symptoms) that allowed evaluating the weight of disease severity on the efficacy of cyclophosphamide. 

The use of high dose steroids in the induction phase (i.e., CYCPRED) or the use of azathioprine as a therapy of consolidation (i.e., CYCAZA) were also not associated with a significant improvement in lung function decline compared to the placebo.

Mycophenolate is widely used as an alternative to, or after failure of, cyclophosphamide to improve lung function in patients with SSc-ILD. Evidence of efficacy of mycophenolate was mainly based on the result of the SLS-II study [7], in which two-year mycophenolate treatment improved FVC % of predicted to a degree that was comparable to one-year cyclophosphamide treatment. However, interpretation of data from the SLS-II trial is limited because of the lack of comparison with the placebo. In this context, a recent joint analysis of results from SLS-I and SLS-II trials reported a significant improvement with mycophenolate in FVC % of predicted and DLCO % of predicted, compared with the placebo [81]. The effect estimate for the efficacy of mycophenolate compared with the placebo in this review was not statistically significant and the certainty of evidence was low due to severe imprecision (as shown by the 95% CI intervals, including the null effect) and study limitations. However, the exclusion of the RCT by Naidu et al. [25] (a study with a small sample size, short follow-up, and high risk of bias) in the sensitivity analysis did not significantly change the effect estimate of mycophenolate.

Given the presence of substantial clinical and pathological similarities between SSc-ILD and idiopathic pulmonary fibrosis (IPF), nintedanib and pirfenidone, two drugs licensed for IPF, have been investigated also in SSc-ILD. In the SENSCIS trial, nintedanib was significantly superior to the placebo in reducing the annual rate of decline in FVC assessed over 52 weeks. However, absence of data on absolute change of FVC % of predicted from baseline to follow-up did not allow to evaluate efficacy of nintedanib on this outcome in the present NMA. 

In the SENSCIS trial, there was no significant difference between nintedanib and the placebo in the decline of DLCO % of predicted (mean (SE) difference −0.44 (−1.94 to 1.06)). 

Similarly, the use of pirfenidone was not associated with significant reduction in decline of DLCO % of predicted compared with the placebo in the RCTs from Acharya et al. [27]. However, as DLCO is also a marker of pulmonary vascular involvement, it is still debated whether it should be considered a valid outcome measure in SSc-ILD. Therefore, the efficacy of treatments on DLCO decline, including nintedanib and pirfenidone, should be interpreted with caution.

Regarding safety, this NMA reported no increased risk of SAEs in SSc-ILD patients treated with different agents compared to the placebo. However, these results should be interpreted with caution considering the low number of trials included in the NMA and the different methods used to report SAEs across trials. Moreover, the relative short follow-up in these trials did not allow assessing possible between-treatment differences in long-term SAEs. 

Regarding tolerability, pomalidomide, cyclophosphamide, and nintedanib were associated with a significantly higher number of patients discontinuing treatment compared to the placebo. Mycophenolate, although not directly compared to the placebo, was associated with a significantly lower discontinuation rate compared to cyclophosphamide. As reported in the meta-analysis from Barnes et al. [78], in accordance with the results of our NMA, patients receiving cyclophosphamide were more likely to discontinue treatment because of AEs such as nausea, leukopenia, and neutropenia. Moreover, the risk of long-term cyclophosphamide-related SAEs, such as cancer, should be taken into account when selecting treatment for SSc-ILD patients. Patients taking nintedanib were also more likely to withdrawn treatment, mostly due to gastrointestinal intolerance [10]. 

Finally, mortality rates were relatively low and not significantly different across treatments and the placebo. However, given the small sample size and the short follow-up, the RCTs included were substantially underpowered to detect such a difference.

To make our results as solid and clinically relevant as possible, we performed a comprehensive literature search (including unpublished data) and restricted our analysis to “lung-focused RCTs,” specifically addressing efficacy and safety of drugs in patients with a definite diagnosis of SSc-ILD based on HRCT and/or lung biopsy. One of the major strengths of our NMA is the comparison of the results from all published RCTs, thus achieving information regarding ranking among treatments even in the absence of evidence from head-to-head trials.

Nonetheless, some limitations of this review and NMA should be acknowledged. First, the small number of RCTs included in the NMA, the presence of only one trial for each comparison, the small simple size in many RCTs, and the large imprecision in the effect estimate may limit the robustness of our results. Second, most of RCTs included in the NMA enrolled SSc-ILD patients with relatively stable or mild disease; therefore, the conclusions of this NMA are not directly applicable to SSc-ILD with severe disease and/or rapid functional decline, a sub-group of patients that are likely to benefit the most from immunosuppression. Third, intervention protocols largely varied across trials, in terms of dose, route of administration, and background medications (i.e., corticosteroids and/or immunosuppressors). Therefore, the possibility that treatment effect might have been influenced by such factors cannot be ruled out. Fourth, as pointed out by The British Thoracic Society (BTS) Interstitial Lung Diseases Guideline, the reproducibility of FVC and DLCO is not optimal in CTD-ILD, particularly in patients with milder forms of the disease, due to the multi-compartment (i.e., vascular, muscular, and pleural) nature of lung involvement [82]. Fifth, possible differences in response according to ILD pattern (NSIP vs. UIP) and disease duration could not be assessed due to the low number of trials and limited available data. Finally, we found the evidence to be of low to very low quality. Certainty of evidence was often downgraded due to severe limitations in the quality of RCTs, indirectness in terms of length of follow-up, and wide confidence intervals including the null effect in the effect estimate, indicating severe imprecision. 

## 5. Conclusions

Based on studies of varying methodological quality and overall low to very low certainty of evidence, we observed a significant benefit with rituximab, in terms of mean difference in FVC % of predicted over one year, in SSc-ILD patients. By contrast, neither cyclophosphamide (alone or combined with high dose steroids or followed by azathioprine), mycophenolate, or pirfenidone were associated with significant reduction in lung functional decline. The effect of nintedanib on FVC decline could not be assessed due to the lack of suitable data. 

Taken collectively, these data suggest a minimal average benefit of current treatments in reducing lung function decline. Pending additional studies, this benefit could be maximized with accurate patient selection for specific therapies. 

Safety, tolerability, and mortality analysis did not demonstrate significant differences between treatments and the placebo, apart from a higher frequency of treatment withdrawal in patients taking nintedanib, pomalidomide, and cyclophosphamide. However, these findings should be interpreted with caution because of trial limitations and low number of events. 

Further adequately powered studies, especially in patients with severe and/or rapidly progressing fibrotic lung disease, are required to examine the efficacy and harms of immunosuppressors and anti-fibrotic drugs for the treatment of SSc-ILD, a condition still in search of effective treatments.

## Supplementary Materials

The following are available online at https://www.mdpi.com/2077-0383/9/8/2560/s1.
