# Supplementary material for: Efficacy, Safety, and Tolerability of Treatments for Systemic Sclerosis-Related Interstitial Lung Disease: A Systematic Review and Network Meta-Analysis
Source: J Clin Med. 2020 Aug 7;9(8):2560. doi: 10.3390/jcm9082560 (PMC7465266; doi:10.3390/jcm9082560)
Supplement: Supplementary file 1 [file jcm-09-02560-s001.pdf]

EFFICACY, SAFETY AND TOLERABILITY OF TREATMENTS FOR SYSTEMIC SCLEROSIS-RELATED INTERSTITIAL LUNG DISEASE: A NETWORK META-ANALYSIS

Erre GL, Sebastiani M, Fenu MA, Zinellu A, Floris A,
Cavagna L, Renzoni E, Manfredi A, Passiu G, Woodman RJ, and Mangoni AA.

Supplementary appendix

INDEX

Abbreviations 4

1. RCTs included in the NMA 5

1.1 Matrix of RCTs with outcomes available for the NMA 5

1.2 Summary of characteristics of RCTs included in the NMA 6

1.3 Characteristics of RCTs 7

1.4 Reference list of RCTs included in the NMA 24

2. Studies excluded from the NMA 25

2.1 List of studies excluded from the NMA and reasons for exclusion 25

2.2 Reference list of studies excluded from the NMA 27

3. Evaluation of Risk of Bias 30

3.1 Summary of Risk of Bias 30

3.2 Graph of Risk of Bias 30

3.3 Overall Risk of Bias of RCTs included in the NMA 31

4. Network meta-analysis 32

4.1 League table change in "FVC % of predicted" 32

4.2 League table change in "DLCO % of predicted" 32

4.3 League table number of patients with serious adverse events 32

4.4 Number of patients discontinuing treatments for adverse events 33

4.5 Deaths 33

5. Netweigth 34

5.1 Change in "FVC % of predicted" 34

5.2 Change in "DLCO % of predicted" 35

5.3 Number of patients with serious adverse events 36

5.4 Number of patients discontinuing treatments for adverse events 37

5.5 Deaths 38

6. Evaluation of inconsistency 39

6.1 Change in "FVC % of predicted" 39

6.1.1 Loop-specific heterogeneity estimate 39

6.1.2 Node-splitting approach 39

6.1.3 Design-by-treatment test 39

6.2 Change in "DLCO % of predicted" 40

6.2.1 Loop-specific heterogeneity estimate 40

6.2.2 Node-splitting approach 40

6.2.3 Design-by-treatment test 40

6.3 Serious adverse events 41

6.3.1 Loop-specific heterogeneity estimate 41

6.3.2 Node-splitting approach 41

6.3.3 Design-by-treatment test 41

7. Quality ratings 42

7.1 GRADE "change in "FVC % of predicted" 42

7.2 GRADE "change in "DLCO % of predicted" 45

7.3 GRADE "number of patients with serious adverse events" 47

7.4 GRADE "number of patients discontinuing treatments for adverse events 48

7.5 GRADE “Deaths”……………………………………………………………………………………………………... 50
8. SUCRAs and cumulative probability plots.. 53
8.1 SUCRA “change in FVC % of predicted”…………………………………………...…………….……... 53
8.2 SUCRA “change in “DLCO % of predicted”…………………………………...…………..……………… 54
8.3 SUCRA “number of patients with serious adverse events………………………………..……………... 55
8.4 SUCRA “number of patients discontinuing treatments for adverse events……………………...……………… 56
8.5 SUCRA “Deaths”……………………………………………………...…………………………….. 57
9. Sensitivity analysis 58
9.1 Effect estimates according to correlation factor……………………………………………………………….. 58
9.1.1 FVC % of predicted…………………………………………………………………………………… 58
9.1.2 DLCO % of predicted…………………………………………………………………………………. 58
9.2 Effect estimates according to length of follow-up…………………………………………………………... 58
9.2.1 FVC % of predicted…………………………………………………………………………………… 58
9.2.2 DLCO % of predicted…………………………………………………………………………………. 58
10. Dataset…………………………………………………………………………………………………... 59
11. Stata syntax………………………………………………………………………………………………... 61
12. Search strategy.. 64

ABBREVIATIONS

**ABA**	Abatacept
**ACR**	American College of Rheumatology
**AEs**	Adverse events
**AMBRI**	Ambrisentan
**Anti-TGFbeta1**	Anti-transforming growth factor beta1
**AZA**	Azathioprine
**BELI**	Belimumab
**CYC**	Cyclophosphamide
**CYCAZA**	Cyclophosphamide + azathioprine
**CYCPRED**	Cyclophosphamide + high dose prednisone
**CRISS**	Combined Response Index for Systemic Sclerosis
**DLCO**	Diffusing capacity of lung for carbon monoxide
**dcSSc**	Diffuse systemic sclerosis
**ESR**	Erythrocyte sedimentation rate
**FVC**	Forced Vital Capacity
**FXIII**	Factor XIII
**HAQ-DI**	Health assessment questionnaire disability index
**HRCT**	High-resolution (chest) computerized tomography
**HSCT**	Haemopoietic stem-cell transplantation
**ILD**	Interstitial lung disease
**IQR**	Interquartile range
**LoF**	Length of follow-up
**LPA**	Lysophosphatidic Acid 1 receptor-antagonist
**MDI**	Mahler Dyspnoea Index
**MMF**	Mycophenolate mofetil
**mRSS**	modified Rodnan skin score
**MTX**	Methotrexate
**n.a**	not available
**NAC**	N-acetylcysteine
**NMA**	Network meta-analysis
**NSIP**	Non Specific Interstitial Pneumonia
**NTD**	Nintedanib
**PAH**	Pulmonary arterial hypertension
**(m)PAP**	(mean)Pulmonary arterial pressure
**PBO**	Placebo
**PFD**	Pirfenidone
**POMA**	Pomalidomide
**RAPA**	Rapamycin
**RIO**	Riociguat
**RoB**	Risk of bias
**RP**	Raynaud’s phenomenon
**SAE**	Serious adverse event
**SF-36**	Medical Outcome Short Form 36
**SSc**	Systemic sclerosis
**SD**	Standard deviation
**VAS**	Analogue scale for pain

1. RCTs INCLUDED IN THE NMA

1.1 Matrix of RCTs with outcomes available for the NMA

	Study	Change FVC % of predicted	Change DLCO % of predicted	Number of patients with SAEs	Number of patients discontinuing treatment for AEs	Deaths
1	SLS-I, 2006	145	145	145	-	145
2	SLS-II, 2016	104	104	104	104	104
3	Domiciano DS, 2011	18	18	-	-	18
4	Hoyles RK, 2006	37	37	-	37	-
5	Naidu GSRSNK, 2020	34	34	34	34	-
6	Sircar G, 2018	60	-	-	-	60
7	SENSCIS, 2019	-	475	475	475	475
8	Acharya N, 2019	34	-	-	34	34
9	Hsu VM, 2018	19	-	19	19	19
	**Total studies**	**8**	**5**	**5**	**6**	**7**
	**Total participants**	**451**	**813**	**777**	**703**	**855**

1.2 Summary of characteristics of RCTs included in the NMA

Study years, country	Arms	Follow-up months	Age years	F %	Dis. duration years	dcSSc %	Criteria SSc-ILD	UIP/NSIP %	Criteria FVC %	Criteria DLCO %	Baseline FVC %	Baseline DLCO %	Low dose steroids	High dose steroids	Other Immunos.	Sponsor
SLS-I<br>2006, USA	CYC<br>PBO	12	48<br>47	75.6<br>64	3.2<br>3.1	62.8<br>57.7	HRCT<br>BAL, PFT	n.a	>45<85	>30	67.6<br>68.6	47<br>47.4	yes	no	yes	no
SLS-II<br>2016, USA	CYC<br>MMF	3, 6<br>12, 24	52.0<br>52.6	78.1<br>69.6	2.5<br>2.6	54.8<br>62.3	HRCT	n.a	>45<85	>40	66.5<br>66.5	54.1<br>54	n.a	n.a	yes	no
Domiciano DS<br>2011, Brazil	CYC<br>CYCPRED	12	44.6<br>41.2	100<br>100	5.8<br>6.0	31.8<br>39.8	Lung biopsy	0/100	n.a	n.a	67.3<br>64.7	61.8<br>69.8	no	yes	no	no
Hoyles RK<br>2006, UK	CYCAZA<br>PBO	12	n.a	77.3<br>75.2	2.7<br>5.5	31.8<br>39.1	HCRT<br>Lung biopsy	n.a	n.a	n.a	80<br>81	52<br>55	prednisolone<br>20 mg alt. day	no	no	no
Naidu GSRSNK<br>2011, India	MMF<br>PBO	6	40.5<br>40	95<br>95.1	4.5<br>3	60<br>38	HRCT	41.5/58.5	>70	n.a	75.6<br>85	43<br>53	prednisolone<br>≤10 mg	no	no	no
SENSCIS<br>2019, multicenter	NTD<br>PBO	12	54.6<br>53.4	76.7<br>73.6	3.4<br>3.5	53.1<br>53.4	HRCT	n.a	>40	>30<89	72.4<br>72.7	52.9<br>53.2	prednisone<br>10 mg/day	no	MMF<br>or MTX	yes
Sircar G<br>2018, India	CYC<br>RTX	6	36.5<br>34.6	83<br>83	1.9<br>1.7	100<br>100	HRCT<br>and PFTs	16.6/80	>45<85	n.a	59.2<br>61.3	n.a	prednisolone<br>10 mg/day	no	no	no
Acharya N<br>2019, India	PFD<br>PBO	6	42<br>40	100<br>82	4<br>3	35.2<br>35.2	HRCT	58.8/35.3<br>64.7/29.4	>50<80	>30	65<br>62.7	45<br>50	prednisone<br>≤10 mg	no	CYC, AZA<br>MMF, MTX	no
Hsu VM<br>2018, multicenter	POMA<br>PBO	12	48.9<br>44.8	90.9<br>83.3	4.7<br>5.3	80<br>75	HRCT	n.a	>45<75	>35<80	57.7<br>60.9	n.a	no	no	no	yes

Alt. day, alternate day; BAL, bronchoalveolar lavage; n.a, not available; dcSSc, diffuse cutaneous SSc; F%, female %; PFTs, pulmonary function tests; UIP/NSIP, HRCT pattern type Usual Interstitial Pneumonia or Non-Specific Interstitial Pneumonia;

1. SLS-I, 2006			
Methods	Design: multicenter, double-blind, randomized, placebo-controlled parallel trial<br>Duration: 1 year of treatment followed by 1 year of observation<br>Location: 13 investigational centres in USA<br>Years: 2000-2004		
Participants	Population: 158 participants were randomised to cyclophosphamide, CYC (79) and placebo PBO (79).		
Baseline characteristics	Age, mean ± SE: 48.2 ± 1.4 CYC, 47.5 ± 1.4 PBO<br>% female: 75.6 CYC, 64.6 PBO<br>Disease duration (mean yrs.): 3.2 ± 0.3 CYC, 3.1 ± 0.2 PBO<br>Diffuse SSc %: 62.8 CYC, 57.7 PBO<br>Baseline FVC % predicted: 67.6 ± 1.5 CYC, 68.6 ± 1.5 PBO<br>Baseline DLCO % predicted: 47.0 ± 1.6 CYC, 47.4 ± 1.6 PBO		
Inclusion criteria	Limited or diffuse SSc with evidence of active alveolitis on bronchoalveolar lavage fluid or thoracic high-resolution computed tomography; any ground-glass opacity; onset of the first symptom of scleroderma other than Raynaud's phenomenon within the previous 7 years; FVC % between 45 and 85 percent of the predicted value; exertional dyspnoea ≥grade 2 on the Magnitude of Task component of the Mahler Baseline Dyspnoea Index.		
Exclusion criteria	DLCO <30 % predicted; history of smoking within the preceding six months; other clinically significant pulmonary abnormalities, or clinically significant pulmonary hypertension requiring drug therapy. Patients taking prednisone >10 mg per day. Patients previously treated >4 weeks with oral cyclophosphamide or >2 intravenous doses. Patients on other potentially disease-modifying medications.		
Interventions	Treatment: oral cyclophosphamide ≤2 mg/kg daily<br>Comparator: placebo		
Concomitant medications	Prednisone at a dose of less than 10 mg per day.		
Primary outcome	Change in FVC % predicted at 24 months.		
Secondary outcomes	Pre-specified secondary outcomes included values at month 12, adjusted for baseline values, for total lung capacity (expressed as a percentage of the predicted value), DLCO, the diffusing capacity adjusted for alveolar volume (Dl:Va), the disability index of the Health Assessment Questionnaire (HAQ), and the Medical Outcomes Study 36-item Short-Form General Health Survey (SF-36).		
Patients available for the analysis	73 CYC, 72 PBO		
Outcomes included in the NMA	1: Change in "FVC % of predicted value at 1 year from baseline<br>2: Change in "DLCO % of predicted value" at 1 year from baseline<br>3: Number of patients with SAEs at the longest available follow-up<br>4: Deaths at the longest available follow-up		
Sponsor	Investigator-initiated		
Funding	Supported by a grant from the Public Health Service and by grants from the National Heart, Lung, and Blood Institute, by the National Institute of Arthritis and Musculoskeletal and Skin Diseases, and by a grant from the National Center for Research Resources, National Institutes of Health.		
Trial registration	n.a		
Summary statistics (outcome)	mean ± SE (1, 2)<br>n (3,4)		
Imputed variables	SD		
Formula	SD = SE*√(n)		
	Summary statistics	CYC n=73	PBO n=72
1. Change FVC % predicted	mean ± SD	-1.0 ± 7.8	-2.6 ± 7.6
2. Change DLCO % predicted	mean ± SD	-4.2 ± 9.9	-3.5 ± 8.4
	Summary statistics	CYC n=79	PBO n=76
3. Number of patients with SAEs	n	20	16
4. Deaths	n	6	6

Risk of Bias	Author's judgment	Support for judgement
Random sequence generation	Low risk	*"Patients who met all the inclusion criteria were randomly assigned with the use of a permuted-block design and a 1:1 allocation (in blocks of four to six patients per center).*
Allocation concealment	Unclear risk	Details not available
Blinding of participants and personnel	Low risk	*"Cyclophosphamide and placebo were formulated into matching gel caps"*
Blinding of outcome assessment	Low risk	"*FVC, the primary endpoint, and the other physiological measures were determined by trained, project-certified hospital-based pulmonary function technologists, Since these technicians were unaware of changes in study medication or the results of other outcomes, it is unlikely that they could have become unintentionally unblinded"*
Incomplete outcome data	Low risk	*"Of a total of 158 patients, 3 assigned to placebo and 1 assigned to cyclophosphamide withdrew before starting study treatment and were not included in the analysis. A total of 20 participants in the cyclophosphamide group and 13 in the placebo group withdrew within 12 months after randomisation, most because of adverse events or serious adverse events. Many participants who withdrew were available for endpoint measurement at 12months; how- ever some 12-month data were extrapolated from 6- or 9-month data. For remaining participants who withdrew prematurely, a generalised estimating- equation regression model was fitted, and data missing at 12 months were imputed. Intention-to-treat analysis was used." "A high percentage of the randomized participants yielded evaluable data that permitted analysis of the primary endpoint (12-month % predicted FVC): 90.1% CYC and 89% placebo subjects*".<br><br>An appropriate imputation method has been employed and the proportion of missing outcome data is 20% or less overall and is balanced between arms.
Selective reporting	Unclear risk	Pre-publication study protocol not available. No trial registration.
Other bias	Low risk	
Overall RoB	Low risk	

2. SLS-II, 2016			
Methods	Design: multicenter, double-blind, randomized, two-arm parallel trial<br>Duration: 24 months.<br>Location: 14 centers in USA.<br>Years: 2009-2013		
Participants	Population: 142 participants were randomised to cyclophosphamide, CYC (73) and mycophenolate mofetil, MMF (69).		
Baseline characteristics	Age, mean ± SD years: 52.0 ± 9.8 CYC, 52.6 ± 9.7 MMF<br>% female: 78.1 CYC, 69.6 MMF<br>Disease duration, mean ± SD years: 2.5 ± 1.8 CYC, 2.6 ± 1.7 MMF<br>Diffuse SSc %: 54.8 CYC, 62.3 MMF<br>Baseline FVC % predicted, mean ± SD: 66.5 ± 8.3 CYC, 66.5 ± 9.1 MMF<br>Baseline DLCO % predicted, mean ± SD: 54.1 ± 14.1 CYC, 54.0 ± 11.1 MMF		
Inclusion criteria	Defined SSc; Age 18–75 years; FVC <80% but ≥ 45% of the predicted value; exertional dyspnoea ≥Grade 2 on the Magnitude of Task component of the Mahler Baseline Dyspnoea Index; any ground glass opacity on whether associated with reticulations (fibrosis) or not; onset of their first non-Raynaud's symptom of SSc within the previous 7 years.		
Exclusion criteria	FVC<45% predicted, FEV1/FVC ratio <65%, pulmonary hypertension DLCO <40% predicted; clinically significant abnormalities on HRCT not attributable to SSc; smoking within the past 6 months; evidence of significant airflow obstruction; persistent unexplained haematuria, leukopenia, thrombocytopenia and clinically significant anaemia. increased liver function test and serum creatinine; uncontrolled congestive heart failure; pregnancy and/or breast feeding; prior use of oral CYC or MMF for more than 8 weeks or the receipt of more than two intravenous doses of CYC in the past; use of CYC and/or MMF in the 30 days prior to randomization; active infection; other serious concomitant medical illness and chronic debilitating illness. Use of medications with disease-modifying properties within the past month.		
Interventions	Treatment: oral cyclophosphamide ≤2 mg/kg daily for 1 year followed by placebo for another year<br>Comparator: mycophenolate mofetil 1500 mg twice daily		
Concomitant medications	n.a		
Primary outcome	Change in FVC % of predicted at 12 and 24 months		
Secondary outcomes	Total lung capacity as a Percent of the Age, Height, Gender, and Ethnicity Adjusted Predicted Value Single-breath<br>DLCO, as a Percent of the Age, Height, Gender, and Ethnicity Adjusted Predicted Value<br>Fibrosis Score, as Measured by HRCT<br>Transitional Dyspnea Index Score<br>HAQ-DI<br>Skin Involvement, as measured by the mRSS<br>Toxicity, as Measured by Adverse Events, Serious Adverse Events, and Death<br>Tolerability, as Assessed by the Time to Withdrawal from the Study Drug or Meeting Protocol-defined Criteria for Treatment Failure.		
Patients available for the analysis	59 MMF, 51 CYC for FVC;<br>58 MMF, 51 CYC for DLCO<br>69 MMF, 73 CYC for the others outcomes		
Outcomes included in the NMA	1: Absolute change in "FVC % of predicted" value at 12 months from baseline<br>2: Absolute change in "DLCO % of predicted" value at 12 months from baseline<br>3: Number of patients with SAEs at the longest available follow-up<br>4: Number of patients discontinuing treatment for AEs<br>5: Deaths at the longest available follow-up<br>Outcomes at 12 months were available on Study Results at ClinicalTrials.gov NCT00883129<br>https://clinicaltrials.gov/ct2/show/results/NCT00883129?term=Tashkin%2C+MMF&draw=2&rank=1		
Sponsor	Investigator-initiated		
Funding	National Heart, Lung and Blood Institute/National Institutes of Health		
Trial registration	ClinicalTrials.gov NCT00883129.		
Summary statistics (outcome)	mean (95% CI) (1, 2)<br>n (3-5)		
Imputed variables	1) SD<br>2) SD of change		
Formula	1) Sample size <60 SD= $\sqrt{n}$*(Upper limit of CI – Lower limit of CI)/2t<br>t value for a 95% confidence interval from a sample size <60 =tinv(0.05, n-1)<br>2) SD change = $\sqrt{SDbaseline^2 + SDfinal^2 - (2*Corr*SDbaseline*SDfinal)}$. Corr=0.8		
	Summary statistics	CYC n=51	MMF n= 59
1.Change FVC % predicted	mean ± SD	3.36 ± 6.6	1.93 ± 6.9

	Summary statistics	CYC n=51	MMF n= 58
2.Change DLCO % predicted	mean ± SD	-7.88 ± 10.3	-5.58 ± 9.3
	Summary statistics	CYC n=73	MMF n= 69
3.Number of patients with SAEs	n	22	27
4. Number of patients discontinuing treatment for AEs	n	15	7
5. Deaths	n	11	5

Risk of Bias (RoB)	Author's judgment	Support for judgement
Random sequence generation	Low risk	*"Randomly assigned patients using a double-blind, double-dummy, center-blocked design"*
Allocation concealment	Low risk	Not explicitly stated. However, it is likely that a process of central allocation (pharmacy controlled) was used.
Blinding of participants and personnel	Low risk	Central pharmacy formulated *"all study drugs (25 mg of CYC, 250 mg of MMF or placebo) into matching 250 mg gel-capsules. Patients received medications as single dose packages containing either 6 or 8 capsules, depending upon patient weight, with the composition of the capsules (active vs placebo) adjusted by the pharmacist to administer the required daily dose while maintaining the blind"*
Blinding of outcome assessment	Low risk	Not stated. However blinding of pulmonary function technologists is likely to be that of the previous study from Tashkin DP (Tashkin DP 2006: "*FVC, the primary endpoint, and the other physiological measures were determined by trained, project-certified hospital-based pulmonary function technologists").*
Incomplete outcome data	Low risk	*"In the CYC arm, 36 patients prematurely stopped drug treatment (2 deaths, 2 treatment failures, and 32 other withdrawals), while only 20 patients in the MMF arm prematurely stopped drug treatment (1 death, 0 treatment failures, 19 other withdrawals)".*<br><br>*"A modified intention-to-treat principle was applied to all analyses using an inferential joint model consisting of a mixed effects model for longitudinal outcomes and a survival model to handle non-ignorable missing data due to study dropout, treatment failure or death (i.e. likely related to disease or treatment and therefore not random). Consistent with the intention-to-treat principle, treatment failures and others who prematurely withdrew from the double-blind treatment phase were encouraged to return for outcome monitoring at the 12, 18 and 24 month visits and their outcomes included in the analysis."*
Selective reporting	Low risk	Protocol is available (ClinicalTrials.gov, NCT00883129). All outcome of interest for this NMA have been reported in the pre-specified way.
Other bias	Low risk	
Overall RoB	Low risk	

3. Domiciano DS, 2011	
Methods	Design: randomized, open-label controlled study<br>Duration: 12 months<br>Location: Brazil<br>Years: 2002-2004
Participants	Population: 18 participants were randomised to cyclophosphamide, CYC (9) and cyclophosphamide + prednisone, CYCPRED (9)
Baseline characteristics	Age, mean ± SD years: 44.6 ± 7.9 CYC, 41.2 ± 10.6 CYCPRED<br>% female: 100<br>Disease duration, mean ± SD years: 5.8 ± 3.9 CYC, 6.0 ± 2.3 CYCPRED<br>Diffuse SSc %: 66.7 CYC, 44.4 CYCPRED<br>Baseline FVC % predicted, mean ± SD: 67.3 ± 16.4 CYC, 64.7 ± 7.7 CYCPRED<br>Baseline DLCO % predicted: mean ± SD: 61.8 ± 16.8 CYC, 69.8 ± 22.5 CYCPRED
Inclusion criteria	Fulfilment of SSc diagnostic criteria.<br>NSIP pattern on lung biopsy
Exclusion criteria	n.a
Interventions	Treatment: CYC IV with monthly infusions of 1 g/m2/dose during 12 months<br>Comparator: CYC+PRED with CYC IV in similar dosage associated to prednisone 60 mg per day during 1 month followed by decreased dosages reaching 10 mg a day on the end of the second month and maintaining the same dose until the end of the treatment
Concomitant medications	Other immunosuppressive agents as D-penicillamine, azathioprine, and methotrexate were not allowed 6 months before and during treatment.
Primary outcomes	Changes in Pulmonary Function Test immediately after treatment (1year) and in prolonged follow-up (after 3 years).
Secondary outcomes	Changes in mRSS<br>Mortality rate
Patients available for the analysis	9 CYC, 9 CYCPRED for FVC<br>5 CYC, 6 CYCPRED for DLCO
Outcomes included in the NMA	1: Absolute change in "FVC % of predicted value" at 1 year from baseline<br>2: Absolute change in "DLCO % of predicted value" at 1 year from baseline<br>3: Deaths at the longest available follow-up
Sponsor	Investigator-initiated
Funding	CNPQ (305468/ 2006-5), Federico Foundation Wilhelm Agricola Research FAPESP (2007/53982-4) and CNPQ (301576/2004-1).
Trial registration	n.a
Summary statistics (outcome)	mean ± SD (1,2)<br>n (3)
Imputed variables	SD of change score
Formula	SD change score = √SDbaseline^2 + SDfinal^2- (2*Corr*SDbaseline*SDfinal). Corr=0.8

	Summary statistics	CYC n=9	CYCPRED n=9
1.Change FVC % predicted	mean ± SD	-2.11 ± 10.7	-0.77 ± 5.8
	Summary statistics	CYC n=5	CYCPRED n=6
2.Change DLCO % predicted	mean ± SD	-14.6 ± 9.1	-4.0 ± 10.2
	Summary statistics	CYC n=9	CYCPRED n=9
3. Deaths	n	1	1

Risk of Bias (RoB)	Author's judgment	Support for judgement
Random sequence generation	Unclear risk	Random sequence generation not defined
Allocation concealment	High risk	Open-label trial
Blinding of participants and personnel	High risk	Open-label trial
Blinding of outcome assessment	High risk	Open-label trial
Incomplete outcome data	High risk	Change in “DLCO % of predicted outcome”: 4/9 patients lost at follow-up in the CYC group and 3/9 in the CYCPRED group.<br>Likely full available set analysis
Selective reporting	Unclear risk	Trial registration not available.
Other bias	Unclear risk	No details available to judge
Overall RoB	High risk	

4. Hoyles RK, 2006	
Methods	Design: Multicenter, Prospective, Randomized, Double-Blind, Placebo-Controlled Trial<br>Duration: 12 months<br>Location: UK<br>Years: 1999-2003
Participants	Population: 45 participants were randomised to cyclophosphamide plus azathioprine, CYCAZA (22) and placebo, PBO (23).
Baseline characteristics	Age: n.a<br>% female: 77.3 CYCAZA, 65.2 PBO<br>Disease duration, median (range) months: 33 (1-204) CYCAZA, 66 (3-322) PBO<br>Diffuse SSc %: 31.8 CYCAZA, 39.1 PBO<br>Baseline FVC % predicted, mean ± SD: 80.1 ± 10.3 CYCAZA, 81.0 ± 18.8 PBO<br>Baseline DLCO % predicted, mean ± SD : 52.9 ± 11.5 CYCAZA, 55.0 ± 12.9
Inclusion criteria	To be included in the study, patients had to be age 18–75 years, fulfil the American College of Rheumatology (ACR; formerly, the American Rheumatism Association) preliminary criteria for a diagnosis of SSc, have SSc-associated pulmonary fibrosis, as indicated by HRCT or thoracoscopic lung biopsy, and comply with therapy and with regular specialty center attendance.
Exclusion criteria	Patients were excluded from the study if they had had previous AZA or CYC therapy for >3 months, had had previous high-dose oral corticosteroid therapy (30 mg of prednisolone or equivalent daily) for >3 months, had had oral corticosteroid therapy (prednisolone dosage >10 mg daily) in the 3 months before study entry, had contraindications to oral corticosteroids such as poorly controlled diabetes or severe osteoporosis, were likely to require lung transplantation within 1 year, had a history of or laboratory data suggestive of other serious systemic or psychological disease unrelated to SSc, were pregnant or lactating, exhibited evidence of alcohol or drug abuse, or were unable to give written informed consent.
Interventions	Treatment: 20 mg oral prednisolone on alternate days and 6 IV infusions of CYC at a dose of 600 mg/m^2 (mean dose 1,050 mg) at 4-week intervals, followed by oral AZA at 2.5 mg/kg/day (maximum 200 mg/day) as maintenance therapy.<br>Comparator: PBO
Concomitant medications	n.a
Primary outcomes	Change in percent predicted FVC and corrected DLCO.
Secondary outcomes	Change in dyspnoea scores (>1 grade), sustained across 2 time points, and change in HRCT extent and pattern of disease at 1 year.
Patients available for the analysis	19 CYCAZA, 18 PBO for FVC and DLCO<br>22 CYCAZA, 23 PBO for number of patients discontinuing treatment for AEs
Outcomes included in the NMA	1: Absolute change in "FVC % of predicted value" at 1 year from baseline<br>2: Absolute change in "DLCO % of predicted value" at 1 year from baseline<br>3: Number of patients discontinuing treatment for AEs
Sponsor	Investigator-initiated
Funding	Supported by the Arthritis Research Campaign (grant 14791) and the Raynaud's & Scleroderma Association, UK.
Trial registration	n.a
Summary statistics (outcome)	Mean ± SD (1,2)<br>n (3)
Imputed variables	SD of change score
Formula	SD change score = √SDbaseline^2 + SDfinal^2- (2*Corr*SDbaseline*SDfinal). Corr=0.8

	Summary statistics	CYCAZA n=19	PBO n=18
1.Change FVC % predicted	mean ± SD	2.4 ± 6.8	-3.0 ± 13.0
2.Change DLCO % predicted	mean ± SD	-3.3 ± 7.0	-3.2 ± 8.9
	Summary statistics	CYCAZA n=22	PBO n=23
3. Number of patients discontinuing treatment for AEs	n	0	2

Risk of Bias (RoB)	Author's judgment	Support for judgement
Random sequence generation	Low risk	*"...minimization method with balancing for the following known prognostic factors: age, baseline HRCT pattern and extent of disease, and autoantibody profile".*
Allocation concealment	Low risk	*"Investigators were blinded to the treatment allocation"* and *"Randomization was undertaken ....by members of the Clinical Trials and Evaluation Unit, who were not involved in the analysis of data."*
Blinding of participants and personnel	Low risk	
Blinding of outcome assessment	Low risk	
Incomplete outcome data	High risk	*"Analysis was based on intent-to-treat subject to the availability of*

		data at 1 year." Missing outcome data at 12-month follow-up: 3/22(13.6%) in the CYCAZA group and 5/23 (21.7%) in the Placebo group. It is not clear whether ITT has been performed imputing missing outcome data.
Selective reporting	Unclear risk	No protocol available. No trial registration.
Other bias	Low risk	
Overall RoB	High risk	

5. Naidu GSRSNK, 2020			
Methods	Design: randomized, double-blind, placebo-controlled study<br>Duration: 6 months<br>Location: India<br>Years: 2016-2018		
Participants	Population:		
Baseline characteristics	Age; median (range) years: 40.5(26-57) MMF, 40(19-61) PBO<br>% female: 95 MMF, 95.1 PBO<br>Disease duration; median (range) years: 4.5(0.75-21) MMF, 3(0.5-40) PBO<br>Diffuse SSc %: 60% MMF, 38% PBO<br>Baseline FVC % predicted, median(range): 75.6(70-94.3) MMF, 85(70-104) PBO<br>Baseline DLCO % predicted, median(range): 43(29-66) MMF, 53(28-81) PBO		
Inclusion criteria	Patients with SSc with presence of ILD on HRCT chest<br>FVC ≥ 70% of predicted on pulmonary function tests<br>Age ≥18 years<br>Consenting for participating in study		
Exclusion criteria	Received immunosuppression (except low dose steroids, prednisolone equivalent ≤10 mg/day) for ILD in the last 3 years<br>Persistent leukopenia or thrombocytopenia<br>Pregnant or breastfeeding females<br>Severe pulmonary arterial hypertension (mean pulmonary arterial pressure >55mmHg) requiring drug therapy<br>Uncontrolled congestive heart failure<br>Any other abnormalities noted on chest X-ray or HRCT other than ILD<br>Active infection<br>Inflammatory myositis<br>Overlap syndrome<br>Mixed connective tissue disease<br>Other serious co-morbidities which could compromise patient's ability to complete the study		
Interventions	Treatment: Mycophenolate Mofetil 500 mg twice a day and increased by 500 mg every 2 weeks, if tolerated, to a target dose of 2gram per day.<br>Comparator: placebo		
Concomitant medications	n.a		
Primary outcome	Change from baseline in FVC at 6 months, after treatment with oral mycophenolate mofetil or placebo		
Secondary outcomes	Change from baseline in QoL score by SF-36 at 6 months<br>Change from baseline in MDI at 6 months<br>Number of participants with serious and non-serious adverse events with MMF and placebo<br>Change in FVC from baseline to 6 months according to antibody profile		
Patients available for the analysis	15 MMF, 19 PBO for FVC and DLCO<br>20 MMF, 21 PBO for the other outcomes		
Outcomes included in the NMA	1: Absolute change in "FVC % of predicted value at 6 months from baseline<br>2: Absolute change in "DLCO % of predicted value at 6 months from baseline<br>3: Number of patients with SAEs at the longest available follow-up<br>4: Number of patients discontinuing treatment for AEs		
Sponsor	Postgraduate Institute of Medical Education and Research, India		
Trial registration	ClinicalTrials.gov, NCT02896205.		
Summary statistics (outcome)	median(range), (1, 2)<br>n (3, 4)		
Imputed variables	mean ± SD		
Formula	Mean ± SD derived from median(range) with online calculator at<br>http://www.math.hkbu.edu.hk/~tongt/papers/median2mean.html<br>Estimated mean of the sample from: Luo D et al. Optimally estimating the sample mean from the sample size, median, mid-range and/or mid-quartile range", *Statistical Methods in Medical Research*, 2018.27:1785-1805.<br>Estimated standard deviation of the sample from: Wan X et al. Estimating the sample mean and standard deviation from the sample size, median, range and/or interquartile range, BMC Medical Research Methodology 2014.14: 135.		
	Summary statistics	MMF n=20	PBO n=21
1.Change FVC % predicted	mean ± SD	-3.68 ± 8.0	1.28 ± 4.2
2.Change DLCO % predicted	mean ± SD	2.93 ± 14.7	1.5 ± 10.8
	Summary statistics	MMF n=20	PBO n=21
3.Number of patients with SAEs	n	1	0

4.Number of patients discontinuing treatment for AEs	n	3	0

Risk of Bias (RoB)	Author's judgment	Support for judgement
Random sequence generation	Low risk	*"All eligible subjects were randomized in a 1:1 ratio in blocks of ten to the two study groups: MMF and placebo."*
Allocation concealment	Low risk	*"Allocation concealment was ensured by enclosing the randomization sequence in sealed opaque envelopes. The primary investigator was blinded to the allocated treatment group of the study subjects and was involved in the assessment of the study subjects during the study period."*
Blinding of participants and personnel	Low risk	*"The study drugs, namely MMF and placebo, were produced by the same manufacturer and provided as tablets of identical shape and colour and were packed into matching boxes. The drugs were dispensed to the subjects by another co-investigator, who was not involved in the randomization process and assessment of the patients."*
Blinding of outcome assessment	Low risk	
Incomplete outcome data	High risk	Drop-out rate 5/20 (25%) in the MMF group and 3/22 (13.6%) in the placebo group. Intention to treat analysis
Selective reporting	Low risk	Protocol available. Trial registration. Outcomes reported in the pre-specified way
Other bias	Low risk	
Overall RoB	High risk	

6. Sircar G, 2018	
Methods	Design: randomized, open-label, parallel-group trial<br>Duration: 6 months<br>Location: India<br>Years: 2016-2017
Participants	Population: 64 participants were randomized to rituximab, RTX(32) and Cyclophosphamide, CYC (32)
Baseline characteristics	Age, mean ± SD years: 34.67 ± 8.13 RTX, 36.5 ± 9.73 CYC<br>% female: 83 RTX, 83 CYC<br>Disease duration, mean ± SD months: 21.5 ± 8.4 RTX, 23 ± 10.1 CYC<br>Diffuse SSc %: 100<br>Baseline FVC % predicted, mean ± SD: 61.3 ± 11.2 RTX, 59.2 ± 12.9 CYC<br>Baseline DLCO % predicted, mean ± SD: n.a
Inclusion criteria	Diffuse SSc fulfilling the 2013 ACR/EULAR classification criteria. Anti-Scl-70 antibody positivity; Age 18-60 years; Presence of interstitial lung disease by high resolution HRCT thorax. FVC% of predicted <80% but at least 45% and reproducible within 10% at the baseline visit; Onset of the patient's first symptom of SSc (including RP) within 3 years of inclusion in the trial. Baseline dyspnoea level New York Heart Association Class II and III.
Exclusion criteria	Any immunosuppression including CYC or RTX of any length before inclusion; pregnancy or breast-feeding; active systemic infections; presence of hepatitis B and C, HIV infections or active tuberculosis; autoimmune overlap syndromes; the New York Heart Association functional class IV symptoms of shortness of breath; presence of moderate to severe pulmonary hypertension (mean pulmonary artery pressure by echocardiogram >40 mmHg), FVC of <45% predicted, ratio of FEV1 (forced expiratory volume during first second) to FVC of <65%; clinical evidence of substantial airflow obstruction; clinically significant abnormalities on HRCT not attributable to SSc; smoking within the past 6 months; persistent unexplained haematuria (>5 red blood cells per high power field); persistent leukopenia or thrombocytopenia; clinically significant anaemia (haemoglobin <80 g/l); baseline AST/ALT 1.5 times the upper limits of normal; serum creatinine >1.3 mg/dl or presence of scleroderma renal crisis and uncontrolled congestive heart failure.
Interventions	Treatment: two RTX pulses of 1000 mg at 0 and 15 days<br>Comparator: 500 mg/m2 of CYC IV pulses every 4 weeks for 24 weeks.
Concomitant medications	Prednisolone 10 mg/day and calcium and vitamin D throughout the course
Primary outcome	Change in FVC % predicted at 24 weeks.
Secondary outcomes	Absolute change in litres (FVC-l) at 6 months; mRSS at 6 months, 6-min walk test, Medsger's score (sum of individual component score) and new onset or worsening of existing pulmonary hypertension (mean pulmonary arterial pressure) estimated by echocardiographic criteria
Patients available for the analysis	30 RTX, 30 CYC
Outcomes included in the NMA	1: Absolute change in "FVC % of predicted value at 6 months from baseline<br>2: Deaths at the longest available follow-up
Sponsor	Investigator-initiated
Funding	No funding sources
Trial registration	India, www.ctri.nic.in, CTRI/2017/07/009152.
Summary statistics (outcome)	mean ± SD (1)<br>n (2)
Imputed variables	SD of change score
Formula	SD change score = $\sqrt{}$SDbaseline^2 + SDfinal^2- (2*Corr*SDbaseline*SDfinal). Corr=0.8

	Summary statistics	RTX n=30	CYC n=30
1.Change FVC % predicted	mean ± SD	6.22 ± 8.1	-1.19 ± 7.8
2.Deaths	n	1	1

Risk of Bias (RoB)	Author's judgment	Support for judgement
Random sequence generation	Low risk	*"A computer-generated random number table was used for simple randomization".*
Allocation concealment	Low risk	*"Opaque, sequentially numbered envelopes were used to determine allocation sequence".*
Blinding of participants and personnel	High risk	Open-label study
Blinding of outcome assessment	High risk	Open-label study
Incomplete outcome data	Low risk	30/32 patients in each group completed the study.
Selective reporting	High risk	Lack of correspondence between primary and secondary outcome reported in the registered protocol (CTRI/2017/07/009152) and reported in the paper. Trial registered retrospectively.
Other bias	Low risk	
Overall RoB	High risk	

7. SENSCIS, 2019	
Methods	Design: randomized, double-blind, placebo-controlled trial<br>Duration: 1 year<br>Location: International, 32 countries.<br>Years: 2015-2017
Participants	Population: 580 participants were randomised to nintedanib, NTD (288) and placebo, PBO (288).
Baseline characteristics	Age, mean ± SD: 54.6 ± 11.8 NTD, 53.4 ± 12.6 PBO<br>% female: 76.7 NTD, 73.6 PBO<br>Disease duration; median (range) yrs.: 3.4 (0.3-7.1) NTD, 3.5(0.4-7.2) PBO<br>Diffuse SSc %: 53.1 NTD, 50.7 PBO<br>Baseline FVC % predicted, mean ± SD: 72.4 ± 16.8 NTD, 72.7 ± 16.6 PBO<br>Baseline DLCO % predicted, mean ± SD: 52.9 ± 15.1 NTD, 53.2 ± 15.1 PBO
Inclusion criteria	SSc according to 2013 ACR/EULAR classification criteria. Age ≥18 yrs. Onset of the first non-Raynaud's symptom within 7 years before screening. Lung fibrosis affecting at least 10% of the lungs at HRTC. FVC ≥40% of the predicted value and a DLCO 30 to 89% of the predicted value.
Exclusion criteria	AST, ALT >1.5 x ULN. 2. Bilirubin >1.5 x ULN. 3. Creatinine clearance <30 mL/min. pre-bronchodilator FEV1/FVC <0.7); Significant pulmonary hypertension; Severe hypertension; Myocardial infarction within 6 months of Visit 1. Unstable cardiac angina within 6 months; More than 3 digital fingertip ulcers at Visit 2 or a history of severe digital necrosis requiring hospitalization; High bleeding risk; History of thrombotic event; Known hypersensitivity to the trial medication or its components; Life expectancy of <2.5 years for disease other than SSc in investigator assessment; Patients with clinical signs of malabsorption or needing parenteral nutrition; Previous treatment with nintedanib or pirfenidone. Other investigational therapy received within 1 month or 6 half-lives (whichever was greater) prior to screening Visit (Visit 1). Treatment with: Prednisone >10 mg/day or equivalent received within 2 weeks prior Visit 2, b. Azathioprine, hydroxychloroquine, colchicine, D-penicillamine, sulfasalazine, received within 8 weeks prior Visit 2, c. Cyclophosphamide, rituximab, tocilizumab, abatacept, leflunomide, tacrolimus, newer anti-arthritic treatments like tofacitinib and cyclosporine A, potassium paraaminobenzoate, received within 6 months prior Visit 2; Unstable background therapy with either mycophenolate mofetil or methotrexate (combined therapy of both not allowed). Patients have to be either a. not on immunosuppressive therapy, or b. on stable therapy with either mycophenolate mofetil or methotrexate for 6 months prior Visit 2 and should stay stable on this background therapy for at least 6 months after randomization; Previous hematopoietic stem cell transplantation (HSCT), or HSCT planned within the next year. Major surgical procedures planned to occur during trial period; Women who are pregnant, nursing, or who plan to become pregnant while in the trial: Women of childbearing potential not willing or able to use highly effective methods of birth control.
Interventions	Treatment: nintedanib 150 mg twice daily<br>Comparator: Placebo
Concomitant medications	Prednisone at a dose of up to 10 mg per day or mycophenolate or methotrexate at a stable dose for at least 6 months before randomization (or both therapies) were allowed as concomitant medications.
Primary outcome	Annual rate of decline in FVC assessed over a 52-week period.
Secondary outcomes	Absolute changes from baseline in the modified Rodnan skin score and in the total score on the St. George's Respiratory Questionnaire at week 52.
Patients available for the analysis	288 NTD, 288 PBO
Outcomes included in the NMA	1: Absolute change in "DLCO % of predicted value" at 52 weeks from baseline<br>2: Number of patients with SAEs at the longest available follow-up<br>3: Number of patients discontinuing treatment for AEs<br>4: Number of deaths at the longest available follow-up
Sponsor	Boehringer Ingelheim
Trial registration	SENSCIS ClinicalTrials.gov number, NCT02597933
Summary statistics (outcome)	mean ± SE (1)<br>n (2-4)
Imputed variables	SD
Formula	SD = SE*√(n)

	Summary statistics	NTD n=287	PBO n=288
1.Change DLCO % predicted	mean ± SD	-3.21 ± 9.1	-2.77 ± 9.1
2.Number of patients with SAEs	n	69	62
3.Number of patients discontinuing treatment for AEs	n	46	25
4.Deaths	n	10	9

Risk of Bias (RoB)	Author's judgment	Support for judgement

Random sequence generation	Low risk	*"The randomisation list will be generated using a validated system, which involves a pseudo-random number generator so that the resulting treatment will be both reproducible and non-predictable. The block size will be documented in the clinical trial report. Access to the codes will be controlled and documented. All members of the clinical trial team will remain blinded to the randomization schedule until the final database is locked."*
Allocation concealment	Low risk	*"Patients, investigators and everyone involved in trial conduct or analysis or with any other interest in this double-blind trial (apart from the DMC) will remain blinded with regard to the randomised treatment assignments until after database lock. The randomization code will be kept secret by the sponsor's clinical trial support up to database lock. The DMC may review unblinded data upon request, and only under conditions that ensure that patients, investigators and everyone involved in trial conduct or analysis or with any other interest in this double-blind trial will remain blinded."*
Blinding of participants and personnel	Low risk	*"Trial medication is identified by a medication code number. Packaging and labelling will be otherwise identical"*
Blinding of outcome assessment	Low risk	*"The effect of missing data will be investigated using multiple imputation methods which assume that patients who discontinue treatment will no longer benefit from it in the future."*
Incomplete outcome data	Low risk	Multiple imputation analysis and sensitivity analysis.
Selective reporting	Low risk	Protocol available. Trial registered at ClinicalTrials.gov, NCT02597933
Other bias	Low risk	
Overall RoB	Low risk	

8. Acharya N, 2019	
Methods	Design: double blind, randomised, placebo-controlled trial<br>Duration: 6 months<br>Location: India<br>Years: 2017-2018
Participants	Population: 34 participants were randomised to pirfenidone, PFD (17) and placebo, PBO (17)
Baseline characteristics	Age; median (range) years: 42(26-55) PFD, 40(20-63) PBO<br>% female: 100 PFD, 82.4 PBO<br>Disease duration, median(range) years: 4(1-7) PFD, 3(0.5-7) PBO<br>Diffuse SSc %: 35 PFD, 35 PBO<br>Baseline FVC % predicted, median(range): 65(51-75) PFD, 62.7(52-78) PBO<br>Baseline DLCO % predicted, median(range): 45(35-65) PFD, 50(34-89) PBO
Inclusion criteria	SSc classified using ACR 2013 classification criteria<br>ILD confirmed on HRCT<br>FVC between 50 and 80% of the predicted<br>DLCO > 30% of the predicted<br>A disease duration of less than seven years since the onset of the first non-Raynaud's symptom<br>No new immunosuppressive treatment administered in the previous six months.
Exclusion criteria	Presence of co-existent inflammatory myopathy<br>Severe PAH requiring specific therapy<br>Persistent cytopenia, any other pulmonary abnormalities on imaging apart from ILD, Clinically significant heart failure<br>Use of biologics in the past<br>Abnormal liver functions (transaminases > 3× upper limit of normal (ULN), bilirubin > 1.5× ULN).
Interventions	Treatment: pirfenidone was started at 600 mg/day and increased to 2400 mg/day over one month and continued for the trial period.<br>Comparator: placebo
Concomitant medications	Subjects receiving stable doses of cyclophosphamide, mycophenolate mofetil, azathioprine, methotrexate and/or prednisolone (or equivalent) ≤ 10 mg/day in the preceding 6 months (or more) were not excluded.
Primary outcome	Primary outcome was to compare the proportion of patients with stabilisation or improvement in lung functions (FVC).
Secondary outcomes	Secondary outcome was to compare the change in FVC, Mahler's dyspnoea index, 6 minute walk distance, mRSS and change in serum levels of tumour necrosis factor α and tissue growth factor β at the end of 6 months.
Patients available for the analysis	17 PFD, 17 PBO
Outcomes included in the NMA	1: Absolute change in "FVC % of predicted value at 12 months from baseline<br>2: Number of patients discontinuing treatment for AEs<br>3: Deaths at the longest available follow-up
Sponsor	Investigator-initiated
Funding	n.a
Trial registration	(CTRI/2018/01/011449)
Summary statistics (outcome)	Median (range) (1)
Imputed variables	mean ± SD (1)<br>n (2, 3)
Formula	Mean ± SD derived from median(range) with online calculator at http://www.math.hkbu.edu.hk/~tongt/papers/median2mean.html<br>Estimated mean of the sample from: Luo D et al. Optimally estimating the sample mean from the sample size, median, mid-range and/or mid-quartile range", *Statistical Methods in Medical Research*, 2018.27:1785-1805.<br>Estimated standard deviation of the sample from: Wan X et al. Estimating the sample mean and standard deviation from the sample size, median, range and/or interquartile range, BMC Medical Research Methodology 2014.14: 135.

	Summary statistics	PFD n= 17	PBO n= 17
1.Change FVC % predicted	mean ± SD	-0.69 ± 4.4	-4.25 ± 14.8
2. Number of patients discontinuing treatment for AEs	n	3	0
3.Deaths	n	0	0

Risk of Bias (RoB)	Author's judgment	Support for judgement
Random sequence generation	Low risk	*"Subjects were randomised in a 1:1 ratio to pirfenidone and placebo groups using a computerised random number generator with blocks of variable size (four or six)".*
Allocation concealment	Low risk	*"Allocation concealment was ensured by enclosing the randomization*

		sequence in sealed opaque envelopes".
Blinding of participants and personnel	Low risk	*"The study subjects, and the investigator, involved in the assessment of outcomes and data analysis were all blinded to the treatment received*".
Blinding of outcome assessment	Low risk	See above
Incomplete outcome data	Low risk	3 Dropouts in PFD group and 1 dropout in PBP group. *"An intention to treat analysis (ITT) was performed for all outcomes".*
Selective reporting	Low risk	Trial registration. Outcomes reported in the pre-specified way
Other bias	Low risk	
Overall RoB	Low risk	

9. Hsu VM, 2018	
Methods	Design: randomized, double-blind, placebo-controlled study<br>Duration: 52 weeks<br>Location: Multicenter<br>Years: 2012
Participants	Population: 23 participants were randomized to Pomalidomide, POMA (11) and placebo, PBO (12)
Baseline characteristics	Age, mean ± SD years: 48.9 ± 9.9 POMA, 44.8 ± 13.8 PBO<br>% female: 90.9 POMA, 83.3 PBO<br>Disease duration, mean years: 4.7 POMA, 5.3 PBO<br>Diffuse SSc %: 80 POMA, 75 PBO<br>Baseline FVC % predicted, mean ± SD years: 57.7 ± 7.3 POMA, 60.9 ± 8.6 PBO
Inclusion criteria	Male or females between 18 and 80 years of age (inclusive) at the time of consent<br>Diagnosis of systemic sclerosis (SSC) as defined by American College of Rheumatology (ACR) criteria<br>Onset of the first non-Raynaud's manifestation of SSC within 7 years of Screening<br>Subjects are required to meet at least one of the following 2 pulmonary-related criteria to be eligible for the study: Forced vital capacity (FVC) ≥ 45% and <70% at Screening and Baseline (Visit 2) [with or without a documented pre-specified FVC decline or fibrosis score] OR FVC readings ≥ 70% and ≤ 80% at Screening and Baseline (Visit 2) with a documented history of either or both of: A ≥ 5% decrease (expressed as percent predicted or in liters) in FVC in the 24-month period prior to Baseline (Visit 2) based on 3 or more assessments. Two assessments may be done during the Screening phase provided the assessments are completed at least 2 weeks apart.<br>A high resolution computed tomography (HRCT) fibrosis score > 20%<br>FVC at Baseline (Visit 2) within 5% of the FVC measured at Screening<br>Carbon monoxide diffusing capacity (DLCO) ≥ 35% and ≤ 80% of predicted value at Screening<br>Abnormalities on High-Resolution CT consistent with parenchymal changes encountered in SSc: honeycombing or reticular changes with or without ground glass.
Exclusion criteria	Oxygen saturation (SpO2) < 92% (room air [sea level] at rest) at Screening or Baseline<br>Known diagnosis of obstructive lung disease as defined by forced expiratory volume (FEV1)/FVC ratio < 0.7<br>Diagnosis of pulmonary arterial hypertension (PAH) requiring treatment<br>Known diagnosis of other significant respiratory disorders (e.g., asthma, tuberculosis, sarcoidosis, aspergillosis, chronic bronchitis, neoplastic disease, cystic fibrosis, etc.)<br>Current clinical diagnosis of another inflammatory connective tissue disease (eg, systemic lupus erythematosus, rheumatoid arthritis, primary Sjogren's syndrome, etc.). Subjects having Sjogren's syndrome secondary to SSc are eligible<br>Pregnant or lactating females<br>History of a thromboembolic event (eg, deep vein thrombosis, thrombotic cerebrovascular or cardiovascular events)<br>History or current diagnosis of peripheral neuropathy<br>Use of concomitant medication(s) which could increase the risk for developing deep vein thrombosis, including sex steroid-based contraceptives (oral, injectable or implanted) and hormone replacement therapies, if use of a low-dose aspirin regimen is contraindicated.<br>Additional concomitant medications which prolong the QT/QTc interval (measure of heart's electrical cycle) during the course of the study<br>Use of any anti-coagulant or anti-thrombotic medications (other than low dose-aspirin [≤ 100 mg/day])<br>Use of any cytotoxic/immunosuppressive agent (other than prednisone ≤ 10 mg/day [mean dose] or equivalent), including but not limited to azathioprine, cyclophosphamide, methotrexate, mycophenolate and cyclosporine within 28 days (4 weeks) of Screening<br>Use of any biologic agent within 84 days (12 weeks) or 5 half-lives of Screening. In the case of rituximab, use within 168 days (24 weeks) of Screening or no recovery of CD20-positive B lymphocytes if the last dose of rituximab has been more than 24 weeks prior to Screening<br>Use of bosentan, ambrisentan, sildenafil, tadalafil and macitentan for PAH within 28 days (4 weeks) of Screening<br>Use of medications (e.g., D-penicillamine, Potaba) with putative scleroderma disease-modifying properties within 4 weeks of Screening<br>Use of melphalan within 52 weeks of Screening<br>Use of any investigational drug within 4 weeks of Screening or 5 pharmacodynamic/pharmacokinetic half-lives if known (whichever is longer)<br>Smoking of cigars, pipes or cigarettes within 24 weeks of Screening
Interventions	Treatment: Pomalidomide 1 mg orally every day for 52 weeks

	Comparator: Placebo		
Concomitant medications	Patients were permitted to continue other supportive medications at stable doses including proton pump inhibitors, angiotensin-converting enzyme inhibitors/angiotensin receptor blockers, and cough medications to treat SSc disease-related symptoms		
Primary outcomes	Number of Participants with Treatment-Emergent Adverse Events (TEAEs)<br>Change from Baseline in Percent Predicted FVC at Week 52<br>Change from Baseline in the Modified Rodnan Skin Score (mRSS) at Week 52/Early Termination<br>Change From Baseline in University of California, Los Angeles, Scleroderma Clinical Trial Consortium Gastrointestinal Tract (UCLA SCTC GIT 2.0) Total Score at Week 52/Early Termination		
Secondary outcomes	Change from Baseline in Percent Predicted Forced Vital Capacity Over Time<br>Change from Baseline in Modified Rodnan Skin Score Over Time<br>Change from Baseline in UCLA SCTC GIT 2.0 Total Score Over Time<br>Change from Baseline in UCLA SCTC GIT 2.0 Reflux Subscale Score Over Time<br>Change from Baseline in UCLA SCTC GIT 2.0 Distension/Bloating Subscale Score Over Time<br>Change from Baseline in UCLA SCTC GIT 2.0 Fecal Soilage Subscale Score Over Time<br>Change from Baseline in UCLA SCTC GIT 2.0 Diarrhoea Subscale Score Over Time<br>Change from Baseline in UCLA SCTC GIT 2.0 Social Functioning Subscale Score Over Time<br>Change from Baseline in UCLA SCTC GIT 2.0 Emotional Well Being Subscale Score Over Time<br>Change from Baseline in UCLA SCTC GIT 2.0 Constipation Subscale Score Over Time<br>Change from Baseline in Dyspnea Functional Impairment at Week 12<br>Change from Baseline in Dyspnea Functional Impairment at Week 24<br>Change from Baseline in Dyspnea Functional Impairment at Week 52/Early Termination<br>Change from Baseline in Dyspnea Functional Impairment at Week 64<br>Change from Baseline in Dyspnea Functional Impairment at Week 76<br>Change from Baseline in Dyspnea Functional Impairment at Week 156/Early Termination<br>Change from Baseline in Dyspnea Magnitude of Task at Week 12<br>Change from Baseline in Dyspnea Magnitude of Task at Week 24<br>Change from Baseline in Dyspnea Magnitude of Task at Week 52/Early Termination<br>Change from Baseline in Dyspnea Magnitude of Task at Week 64<br>Change from Baseline in Dyspnea Magnitude of Task at Week 76<br>Change from Baseline in Dyspnea Magnitude of Task at Week 156/Early Termination<br>Change from Baseline in Dyspnea Magnitude of Effort at Week 12<br>Change from Baseline in Dyspnea Magnitude of Effort at Week 24<br>Change from Baseline in Dyspnea Magnitude of Effort at Week 52/Early Termination<br>Change from Baseline in Dyspnea Magnitude of Effort at<br>Change from Baseline in Dyspnea Magnitude of Effort at Week 76<br>Change from Baseline in Dyspnea Magnitude of Effort at Week 156/Early Termination<br>Oxygen Saturation Over Time<br>Pharmacokinetic Parameters of Pomalidomide in Plasma		
Patients available for the analysis	10 POMA, 12 PBO		
Outcomes included in the NMA	1: Absolute change in "FVC % of predicted value" at 1 year from baseline<br>2: Number of patients with SAEs at the longest available follow-up<br>3: Number of patients discontinuing treatment for AEs<br>4: Deaths at the longest available follow-up		
Sponsor	Celgene		
Trial registration	ClinicalTrials.gov, NCT01559129		
Summary statistics (outcome)	mean ± SD (1)<br>n (2-4)		
	Summary statistics	POMA n=8	PBO n=11
1.Change FVC % predicted	mean ± SD	-5.2 ± 5.3	-2.8 ± 4.0
2. Number of patients discontinuing treatment for AEs	n	4	1
3.Number of withdrawals	n	4	0
4.Deaths	n	0	0
Risk of Bias (RoB)	Author's judgment	Support for judgement	
Random sequence generation	Unclear risk	Details not available	

Allocation concealment	Unclear risk	Details not available
Blinding of participants and personnel	Unclear risk	Details not available
Blinding of outcome assessment	Unclear risk	Details not available
Incomplete outcome data	High risk	*"Of these 22 patients, 11 (50.0%) completed 52 weeks, with more PBO patients (7, 58.3%) completing treatment versus (4, 36.4%) POM patients.*
Selective reporting	High risk	Protocol is available (Clinical Trials number: NCT01559129). Not all outcome of interest have been reported in the pre-specified way.
Other bias	Low risk	
Overall RoB	High risk	

1.4 Reference list of RCTs included in the NMA

1. SLS-I, 2006
Tashkin DP, Elashoff R, Clements PJ, et al. Cyclophosphamide versus placebo in scleroderma lung disease. N Engl J Med. 2006;354(25):2655–66.

2. SLS-II, 2016
Tashkin DP, Roth MD, Clements PJ, et al. Mycophenolate Mofetil versus Oral Cyclophosphamide in Scleroderma-related Interstitial Lung Disease: Scleroderma Lung Study II (SLS-II), a double-blind, parallel group, randomised controlled trial. Lancet Respir Med. 2017;4(9):708–19.

3. Domiciano DS, 2011
Domiciano DS, Bonfá E, Borges CT, et al. A long-term prospective randomized controlled study of non-specific interstitial pneumonia (NSIP) treatment in scleroderma. Clin Rheumatol. 2011;30(2):223–9.

4. Hoyles RK, 2006
Hoyles RK, Ellis RW, Wellsbury J, et al. A multicenter, prospective, randomized, double-blind, placebo-controlled trial of corticosteroids and intravenous cyclophosphamide followed by oral azathioprine for the treatment of pulmonary fibrosis in scleroderma. Arthritis Rheum. 2006;54(12):3962–70.

5. Naidu GSRSNK, 2020
Naidu GSRSNK, Sharma SK, Adarsh MB, Dhir V, Sinha A, Dhooria S, et al. Effect of mycophenolate mofetil (MMF) on systemic sclerosis-related interstitial lung disease with mildly impaired lung function: a double-blind, placebo-controlled, randomized trial. Rheumatol Int [Internet]. 2019;(0123456789). Available from: https://doi.org/10.1007/s00296-019-04481-8

6. Sircar G, 2018
Sircar G, Goswami RP, Sircar D, Ghosh A, Ghosh P. Intravenous cyclophosphamide vs rituximab for the treatment of early diffuse scleroderma lung disease: Open label, randomized, controlled trial. Rheumatol (United Kingdom). 2018;57(12):2106–13.

7. SENSCIS, 2019
Distler O, Highland KB, Gahlemann M, Azuma A, Fischer A, Mayes MD, et al. Nintedanib for systemic sclerosis-associated interstitial lung disease. N Engl J Med. 2019;380(26):2518–28.

8. Acharya N, 2019
Acharya N, Sharma SK, Mishra D, Dhooria S, Dhir V, Jain S. Efficacy and safety of pirfenidone in systemic sclerosis-related interstitial lung disease-a randomised controlled trial. *Rheumatol Int*. 2020;40(5):703–710. doi:10.1007/s00296-020-04565-w

9. Hsu VM, 2018
Hsu VM, Denton CP, Domsic RT, et al. Pomalidomide in patients with interstitial lung disease due to systemic sclerosis: A phase II, multicenter, randomized, double-blind, placebo-controlled, parallel-group study. J Rheumatol. 2018;45(3):405–10.

2. Studies excluded from the NMA

2.1 List of studies excluded from the NMA and reasons for exclusion

	Study	Arms	Reason of exclusion
1	Abou-Raya A, 2013	Irbesartan, PBO	Data incomplete
2	Allanore Y, 2018	LPA1-r antagonist, PBO	Short follow-up
3	Allanore Y, 2019	Romilkimab, PBO	Data incomplete
4	ASSET, 2019	ABA, PBO	No definite diagnosis of SSc-ILD
5	ASSIST, 2011	HSCT, PBO	No definite diagnosis of SSc-ILD
6	Boonstra M, 2017	RTX, PBO	No definite diagnosis of SSc-ILD
7	Chakravarty EF, 2015	ABA, PBO	No definite diagnosis of SSc-ILD
8	Daoussis D, 2010	RTX	Observational study
9	Daoussis D, 2012	RTX	Observational study
10	Daoussis D, 2017	RTX	Observational study
11	Denton C, 2007	Anti–TGFβ 1 (CAT-192), PBO	Data incomplete
12	EDITA, 2019	Ambrisentan, PBO	No definite diagnosis of SSc-ILD
13	FaSScinate, 2016	Tocilizumab, PBO	No definite diagnosis of SSc-ILD
14	FocuSSced, 2020	Tocilizumab, PBO	No definite diagnosis of SSc-ILD
15	Gordon JK, 2018	Belimumab, PBO	No definite diagnosis of SSc-ILD
16	Gruber BL, 1991	Ketotifen, PBO	Data incomplete
17	Guillevin L, 1982	FXIII, PBO	Data incomplete
18	Guo M.-H. M.-H, 2008	Penicillamine, Yiqi-Huoxue medicine	No connections in the network
19	Henes J, 2020	HSCT, PBO	Observational study
20	Herrick AL, 2017	CYC	Observational study
21	Hoffman-Vold AM, 2019	Fecal transplantation, PBO	Data incomplete
22	Khanna D, 2009	Relaxin, PBO	No definite diagnosis of SSc-ILD
23	Khanna D, 2019	Tofacitinib, PBO	Data incomplete
24	Mehrabi S, 2019	NAC, PBO	Data incomplete
25	Nadashkevich O, 2008	CYC, AZA	No definite diagnosis of SSc-ILD
26	NCT02283762	Riociguat, PBO	No definite diagnosis of SSc-ILD
27	NCT02465437	Lenabasum (JBT-101), PBO	Short follow-up
28	NCT02745145	Abituzumab, PBO	Outcomes expressed in a format not suitable for NMA
29	Pakas J, 2002	CYC+low, CYC+high dose steroids	Observational study
30	Panopoulos ST, 2013	CYC, MMF	Observational study
31	Poormoghim H, 2013	CYC, AZA	Observational study
32	Pope JE, 2001	Methotrexate, PBO	No definite diagnosis of SSc-ILD
33	Prey S, 2012	Imatinib, PBO	Data incomplete
34	Quillinan NP, 2014	Hyperimmune caprine serum, PBO	Data incomplete
35	Schiopu E, 2016	Anti-CD19, PBO	Short follow-up
36	Sclero XIII, 2019	FXIII, PBO	No definite diagnosis of SSc-ILD
37	Seibold JR, 2000	Relaxin, PBO	No definite diagnosis of SSc-ILD
38	Seibold JR, 2010	Bosentan, PBO	Outcomes expressed in a format not suitable for NMA
39	Su TIK, 2009	Rapamycin, methotrexate	No definite diagnosis of SSc-ILD
40	Sullivan A, 2018	HSCT, CYC	Outcomes expressed in a format not suitable for NMA
41	van den Hoogen FHJ, 1996	MTX, PBO	Data incomplete

42	van Laar JM, 2014	HSCT, CYC	Outcomes expressed in a format not suitable for NMA

2.2 Reference list of studies excluded from the NMA

1. Abou-Raya A, 2013
Abou-Raya A, Abou-Raya S, Helmii M. OP0038 Effects of Angiotensin II Receptor Blockade in Systemic Sclerosis: Randomized Controlled Trial. Ann Rheum Dis 2013; 72: A61.

2. Allanore Y, 2018
Allanore Y, Distler O, Jagerschmidt A, *et al.* Lysophosphatidic Acid Receptor 1 Antagonist SAR100842 for Patients with Diffuse Cutaneous Systemic Sclerosis: A Double-Blind, Randomized, Eight-Week Placebo-Controlled Study Followed by a Sixteen-Week Open-Label Extension Study. Arthritis Rheumatol 2018; 70:1634-1643. doi:10.1002/art.40547.

3. Allanore Y, 2019
Allanore Y, Denton C, Khanna D, et al. Efficacy and Safety of Romilkimab in Diffuse Cutaneous Systemic Sclerosis (dcSSc): A Randomized, Double-Blind, Placebo-Controlled, 24-week, Proof of Concept Study [abstract]. *Arthritis Rheumatol.* 2019; 71 (suppl 10).

4. ASSET, 2019
Khanna D, Spino C, Johnson S, et al. Abatacept in Early Diffuse Cutaneous Systemic Sclerosis: Results of a Phase II Investigator-Initiated, Multicenter, Double-Blind, Randomized, Placebo-Controlled Trial. Arthritis Rheumatol. 2020;72(1):125–36.

5. ASSIST, 2011
Burt RK, Shah SJ, Dill K, et al. Autologous non-myeloablative haemopoietic stem-cell transplantation compared with pulse cyclophosphamide once per month for systemic sclerosis (ASSIST): an open-label, randomised phase 2 trial. Lancet [Internet]. 2011;378(9790):498–506. Available from: http://dx.doi.org/10.1016/S0140-6736(11)60982-3

6. Boonstra M, 2017
Boonstra M, Meijs J, Dorjée AL, et al. Rituximab in early systemic sclerosis. RMD Open. 2017;3(2).

7. Chakravarty EF, 2015
Chakravarty EF, Martyanov V, Fiorentino D, et al. Gene expression changes reflect clinical response in a placebo-controlled randomized trial of abatacept in patients with diffuse cutaneous systemic sclerosis. Arthritis Res Ther [Internet]. 2015;1–14. Available from: http://dx.doi.org/10.1186/s13075-015-0669-3

8. Daoussis D, 2010
Daoussis D, Liossis SN, Tsamandas AC, et al. Experience with rituximab in scleroderma: results from a 1-year, proof-of-principle study. Rheumatology (Oxford) 2010; 49:271-80. doi: 10.1093/rheumatology/kep093.

9. Daoussis D, 2012
Daoussis D, Liossis SN, Tsamandas AC, et al. Effect of long-term treatment with rituximab on pulmonary function and skin fibrosis in patients with diffuse systemic sclerosis. Clin Exp Rheumatol 2012;30(2 Suppl 71): S17-22.

10. Daoussis D, 2017
Daoussis D, Melissaropoulos K, Sakellaropoulos G, et al. A multicenter, open-label, comparative study of B-cell depletion therapy with Rituximab for systemic sclerosis-associated interstitial lung disease. Semin Arthritis Rheum 2017; 46:625-631. doi: 10.1016/j.semarthrit.2016.10.003.

11. Denton CP, 2007
Denton CP, Merkel PA, Furst DE, et al. Recombinant human anti-transforming growth factor beta1 antibody therapy in systemic sclerosis: a multicenter, randomized, placebo-controlled phase I/II trial of CAT-192. Arthritis Rheum 2007; 56:323-33.

12. EDITA, 2019
Pan Z, Marra AM, Benjamin N, et al. Early treatment with ambrisentan of mildly elevated mean pulmonary arterial pressure associated with systemic sclerosis: A randomized, controlled, double-blind, parallel group study (EDITA study). Arthritis Res Ther. 2019;21(1):1–15.

13. FaSScinate, 2016
Khanna D, Denton CP, Lin CJF, et al. Safety and efficacy of subcutaneous tocilizumab in systemic sclerosis: Results from the open-label period of a phase II randomised controlled trial (faSScinate). Ann Rheum Dis. 2018;77(2):212–20.

14. FocuSSced, 2020
NCT02453256. A Study of the Efficacy and Safety of Tocilizumab in Participants with Systemic Sclerosis (SSc) [focuSSced] [Internet]. 2020. Available from: https://clinicaltrials.gov/ct2/show/NCT02453256

15. Gordon JK, 2018
Gordon JK, Martyanov V, Franks JM, Bernstein EJ, et al. Belimumab for the Treatment of Early Diffuse Systemic Sclerosis: Results of a Randomized, Double-Blind, Placebo-Controlled, Pilot Trial. Arthritis Rheumatol. 2018;70(2):308–16.

16. Gruber BL, 1991

Gruber BL, Kaufman LD. A double-blind randomized controlled trial of ketotifen versus placebo in early diffuse scleroderma. Arthritis Rheum 1991; 34:362-6.

17. Guillevin L, 1982
Guillevin L, Chouvet B, Mery C, et al. [Treatment of generalized scleroderma with factor XIII. Study of 25 cases. Rev Med Interne 1982;3(3):273-7.

18. Guo MH, 2008
Guo MH, Tu WZ, Chen DD, et al. Therapeutic effects on systemic scleroderma of integrated therapy of Traditional Chinese Medicine with penicillamine. J Clin Derm 2008; 7

19. Henes J, 2019
Henes J, Oliveira MC, Labopin M, et al. Autologous stem cell transplantation for progressive systemic sclerosis: a prospective non-interventional study from the European Society for Blood and Marrow Transplantation Autoimmune Disease Working Party. Haematologica 2020;16. doi: 10.3324/haematol.2019.230128.

20. Herrick AL, 2019
Herrick AL, Pan X, Peytrignet S, et al. Treatment outcome in early diffuse cutaneous systemic sclerosis: The European Scleroderma Observational Study (ESOS). Ann Rheum Dis. 2017 Jul;76(7):1207-1218. doi: 10.1136/annrheumdis-2016-210503.

21. Hoffmann-Vold A, 2019
Hoffmann-Vold A, Fretheim H, Chung BK, et al. OP03207 Fecal microbiota transplantation in systemic sclerosis: a double-blind, placebo-controlled randomized pilot trial. Ann Rheum Dis 2019; 78:246-247.

22. Khanna D, 2009
Khanna D, Clements PJ, Furst DE, et al. Recombinant human relaxin in the treatment of systemic sclerosis with diffuse cutaneous involvement: A Randomized, Double-Blind, Placebo-Controlled Trial. Arthritis Rheum. 2009;60(4):1102–11.

23. Khanna D, 2019
Khanna D, Bush E, Nagaraja V, et al. Results of Phase I/II Investigator-Initiated, Double-Blind Randomized Placebo-Controlled Trial [abstract]. Arthritis Rheumatol 2019; 71 (suppl 10). https://acrabstracts.org/abstract/tofacitinib-in-early-diffuse-cutaneous-systemic-sclerosis-results-of-phase-i-ii-investigator-initiated-double-blind-randomized-placebo-controlled-trial/.

24. Mehrabi S, 2019
Mehrabi S, Moradi MM, Khodamoradi Z, et al. Effects of N-acetylcysteine on Pulmonary Functions in Patients with Systemic Sclerosis: A double blind, placebo controlled study. Curr Rheumatol Rev 2019;10.2174. doi:10.2174/1573397115666191212092608

25. Nadashkevich O, 2008
Nadashkevich O, Davis P, Fritzler M, Kovalenko W. A randomized unblinded trial of cyclophosphamide versus azathioprine in the treatment of systemic sclerosis. Clin Rheumatol. 2006;25(2):205–12.

26. NCT02283762, 2020
NCT02283762. Efficacy and Safety of Riociguat in Patients with Systemic Sclerosis [Internet]. 2020. Available from: https://clinicaltrials.gov/ct2/show/NCT02283762

27. NCT02465437
Safety, Tolerability, Efficacy, and Pharmacokinetics of JBT-101 in Systemic Sclerosis.
Available at: https://clinicaltrials.gov/ct2/show/study/NCT02465437

28. NCT02745145
Abituzumab in SSc-ILD. Available at
Available at: https://clinicaltrials.gov/ct2/show/results/NCT02745145?term=NCT02745145&draw=2&rank=1

29. Pakas J, 2002
Pakas I, Ioannidis JP, Malagari K, et al. Cyclophosphamide with low or high dose prednisolone for systemic sclerosis lung disease. J Rheumatol 2002; 29:298-304.

30. Panopoulos ST, 2013
Panopoulos ST, Bournia VK, Trakada G, et al. Mycophenolate versus cyclophosphamide for progressive interstitial lung disease associated with systemic sclerosis: a 2-year case control study. Lung 2013; 191: 483-9. doi: 10.1007/s00408-013-9499-8.

31. Poormoghim H, 2013
Poormoghim H, Rezaei N, Sheidaie Z, et al. Systemic sclerosis: comparison of efficacy of oral cyclophosphamide and azathioprine on skin score and pulmonary involvement-a retrospective study. Rheumatol Int. 2014 Dec;34(12):1691-9. doi: 10.1007/s00296-014-3026-y.

32. Pope JE, 2001
Pope JE, Bellamy N, Seibold JR, Baron M, Ellman M, Carette S, et al. A randomized, controlled trial of methotrexate versus placebo in early diffuse scleroderma. Arthritis Rheum. 2001;44(6):1351–8.

33. Prey S, 2012
Prey S, Ezzedine K, Doussau A, et al. Imatinib mesylate in scleroderma-associated diffuse skin fibrosis: a phase II multicentre randomized double-blinded controlled trial. Br J Dermatol 2012; 167: 1138-44. doi: 10.1111/j.1365-2133.2012.11186. x.

34. Quillinan NP, 2013
Quillinan NP, McIntosh D, Vernes J, et al. Treatment of diffuse systemic sclerosis with hyperimmune caprine serum (AIMSPRO): a phase II double-blind placebo-controlled trial. *Ann Rheum Dis* 2014; 73:56–61. doi:10.1136/annrheumdis-2013-203674

35. Schiopu E, 2016
Schiopu E, Chatterjee S, Hsu V, Flor A, et al. Safety and tolerability of an anti-CD19 monoclonal antibody, MEDI-551, in subjects with systemic sclerosis: a phase I, randomized, placebo-controlled, escalating single-dose study. Arthritis Res Ther. 2016 Jun 7;18(1):131. doi:10.1186/s13075-016-1021-2.

36. Sclero XIII, 2019
EudraCT 2014-001101-40. Sclero XIII: a phase II, double-blind, randomised, placebo-controlled study to investigate the pharmacokinetics, safety and efficacy of intravenous factor XIII treatment in patients with systemic sclerosis [Internet]. 2019. Available from: https://www.clinicaltrialsregister.eu/ctr-search/search?query=Sclero+XIII

37. Seibold JR, 2000
Seibold JR, Korn JH, Simms R, Clements PJ, Moreland LW, Mayes MD, et al. Recombinant human relaxin in the treatment of scleroderma. A randomized, double-blind, placebo-controlled trial. Ann Intern Med. 2000;132(11):871–9.

38. Seibold JR, 2010
Seibold JR, Denton CP, Furst DE, et al. Randomized, prospective, placebo-controlled trial of bosentan in interstitial lung disease secondary to systemic sclerosis. Arthritis Rheum 2010; 62:2101-8. doi: 10.1002/art.27466.

39. Su TIK, 2009
Su TIK, Khanna D, Furst DE, Danovitch G, Burger C, Maranian P, et al. Rapamycin versus methotrexate in early diffuse systemic sclerosis: Results from a randomized, single-blind pilot study. Arthritis Rheum. 2009;60(12):3821–30.

40. Sullivan KM, 2018
Sullivan KM, Goldmuntz EA, Keyes-Elstein L, et al. Myeloablative Autologous Stem-Cell Transplantation for Severe Scleroderma. N Engl J Med 2018; 378: 35-47.

41. van den Hoogen FH, 1996
van den Hoogen FH, Boerbooms AM, Swaak AJ, Rasker JJ, van Lier HJ, van de Putte LB. Comparison of methotrexate with placebo in the treatment of systemic sclerosis: a 24 week randomized double-blind trial, followed by a 24 week observational trial. Br J Rheumatol 1996; 35:364-72.

42. van Laar JM, 2014
van Laar JM, Farge D, Sont JK, et al. Autologous hematopoietic stem cell transplantation vs intravenous pulse cyclophosphamide in diffuse cutaneous systemic sclerosis: a randomized clinical trial. JAMA. 2014 Jun 25;311(24):2490-8. doi: 10.1001/jama.2014.6368.

3. Evaluation of Risk of Bias

3.1 Summary of Risk of Bias

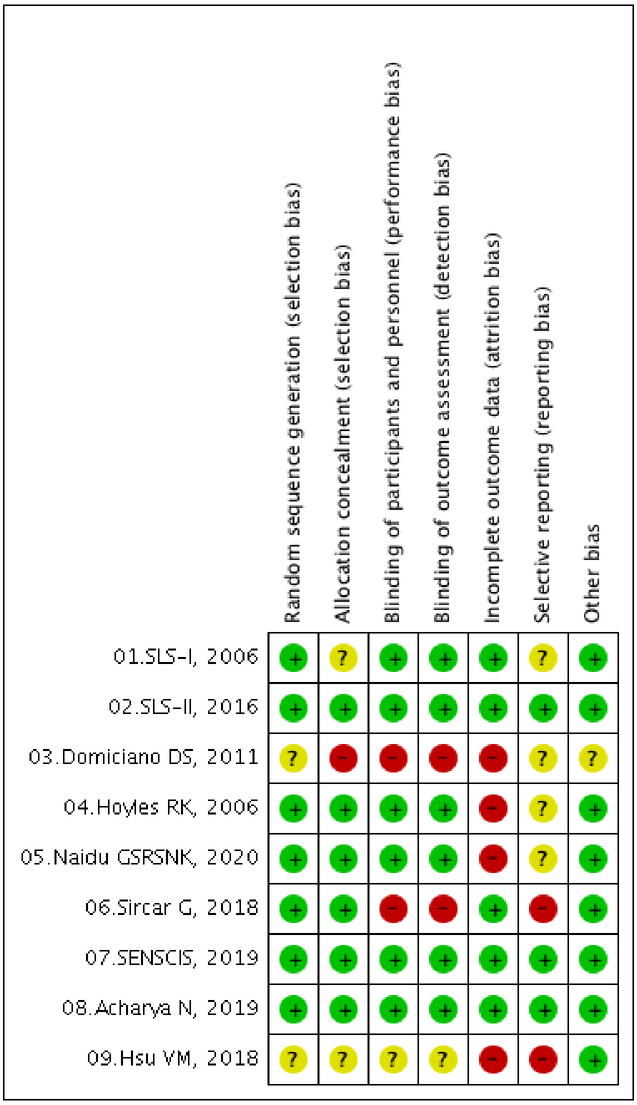

Review authors' judgements about each RoB item for each included RCT

3.2 Graph of Risk of Bias

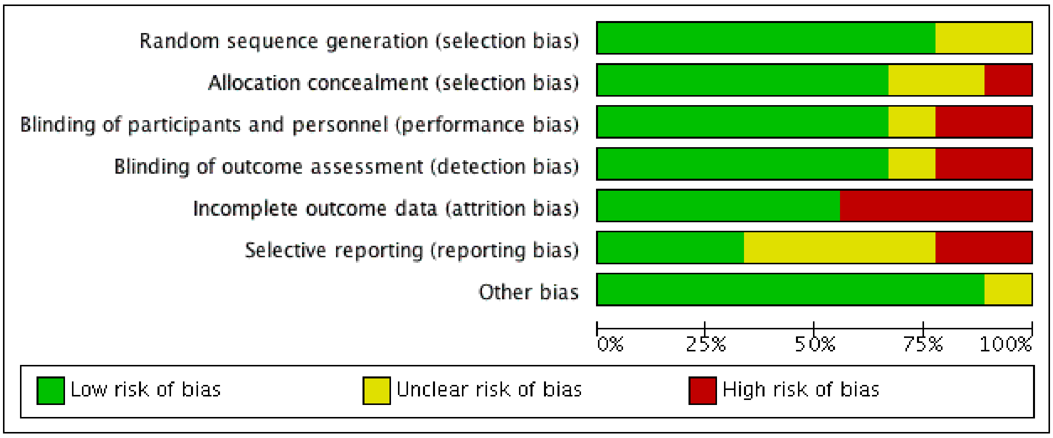

Review authors' judgements about each RoB item presented as percentages across all included RCTs

3.3 Overall Risk of Bias of RCTs included in the NMA

	Study	Arms	Overall RoB
1	SLS-I, 2006	Cyclophosphamide vs placebo	Low risk
2	SLS-II, 2016	Cyclophosphamide vs mycophenolate	Low risk
3	Domiciano DS, 2011	CYCPRED vs cyclophosphamide	High risk
4	Hoyles RK, 2006	CYCAZA vs placebo	High risk
5	Naidu GSRSNK, 2020	Mycophenolate vs placebo	High risk
6	Sircar G, 2018	Rituximab vs cyclophosphamide	High risk
7	SENSCIS, 2019	Nintedanib vs placebo	Low risk
8	Acharya N, 2019	Pirfenidone vs placebo	Low risk
9	Hsu VM, 2018	Pomalidomide vs placebo	High risk

Overall RoB of each RCT was assessed as follows: 1) Low RoB: none of the seven domains was rated as high RoB and three or less were rated as unclear risk; 2) Moderate RoB: one was rated as high risk of bias or none was rated as high risk of bias but four or more were rated as unclear risk 3) High RoB: all other combinations.

4. Network meta-analysis

4.1 League table "change in FVC % of predicted"

**PBO**	0.08 (-0.22,0.38)	-0.29 (-0.69,0.10)	0.23 (-0.74,1.20)	0.51 (-0.14,1.17)	1.00 (0.39,1.61)	0.32 (-0.36,1.00)	-0.50 (-1.43,0.43)
-0.08 (-0.38,0.22)	**CYC**	-0.38 (-0.71,-0.04)	0.15 (-0.78,1.07)	0.43 (-0.29,1.15)	0.92 (0.39,1.45)	0.24 (-0.50,0.98)	-0.58 (-1.56,0.39)
0.29 (-0.10,0.69)	0.38 (0.04,0.71)	**MMF**	0.52 (-0.46,1.51)	0.81 (0.04,1.57)	1.29 (0.67,1.92)	0.61 (-0.17,1.39)	-0.21 (-1.21,0.80)
-0.23 (-1.20,0.74)	-0.15 (-1.07,0.78)	-0.52 (-1.51,0.46)	**CYCPRED**	0.28 (-0.89,1.46)	0.77 (-0.30,1.84)	0.09 (-1.10,1.27)	-0.73 (-2.07,0.61)
-0.51 (-1.17,0.14)	-0.43 (-1.15,0.29)	-0.81 (-1.57,-0.04)	-0.28 (-1.46,0.89)	**CYCAZA**	0.49 (-0.41,1.39)	-0.20 (-1.14,0.75)	-1.01 (-2.15,0.12)
-1.00 (-1.61,-0.39)	-0.92 (-1.45,-0.39)	-1.29 (-1.92,-0.67)	-0.77 (-1.84,0.30)	-0.49 (-1.39,0.41)	**RTX**	-0.68 (-1.60,0.23)	-1.50 (-2.61,-0.39)
-0.32 (-1.00,0.36)	-0.24 (-0.98,0.50)	-0.61 (-1.39,0.17)	-0.09 (-1.27,1.10)	0.20 (-0.75,1.14)	0.68 (-0.23,1.60)	**PFD**	-0.82 (-1.97,0.33)
0.50 (-0.43,1.43)	0.58 (-0.39,1.56)	0.21 (-0.80,1.21)	0.73 (-0.61,2.07)	1.01 (-0.12,2.15)	1.50 (0.39,2.61)	0.82 (-0.33,1.97)	**POMA**

4.2 League table "change in DLCO % of predicted"

**PBO**	-0.08 (-0.38,0.21)	0.14 (-0.25,0.53)	0.90 (-0.44,2.24)	-0.01 (-0.66,0.63)	-0.05 (-0.21,0.12)
0.08 (-0.21,0.38)	**CYC**	0.22 (-0.11,0.55)	0.98 (-0.32,2.29)	0.07 (-0.64,0.78)	0.04 (-0.30,0.37)
-0.14 (-0.53,0.25)	-0.22 (-0.55,0.11)	**MMF**	0.76 (-0.59,2.11)	-0.15 (-0.90,0.60)	-0.19 (-0.61,0.23)
-0.90 (-2.24,0.44)	-0.98 (-2.29,0.32)	-0.76 (-2.11,0.59)	**CYCPRED**	-0.91 (-2.40,0.57)	-0.95 (-2.30,0.40)
0.01 (-0.63,0.66)	-0.07 (-0.78,0.64)	0.15 (-0.60,0.90)	0.91 (-0.57,2.40)	**CYCAZA**	-0.04 (-0.70,0.63)
0.05 (-0.12,0.21)	-0.04 (-0.37,0.30)	0.19 (-0.23,0.61)	0.95 (-0.40,2.30)	0.04 (-0.63,0.70)	**NTD**

4.3 League table "number of patients with SAEs"

**PBO**	0.32 (-0.42, 1.06)	0.67 (-0.36, 1.70)	0.14 (-0.25, 0.53)	2.30 (-0.18, 4.78)
-0.32 (-1.06, 0.42)	**CYC**	0.35 (-0.40, 1.11)	-0.17 (-1.01, 0.66)	1.99 (-0.60, 4.57)
-0.67 (-1.70, 0.36)	-0.35 (-1.11, 0.40)	**MMF**	-0.53 (-1.63, 0.57)	1.63 (-1.05, 4.31)
-0.14 (-0.53, 0.25)	0.17 (-0.66, 1.01)	0.53 (-0.57, 1.63)	**NTD**	2.16 (-0.35, 4.67)
-2.30 (-4.78, 0.18)	-1.99 (-4.57, 0.60)	-1.63 (-4.31, 1.05)	-2.16 (-4.67, 0.35)	**POMA**

4.4 League table "number of patients discontinuing treatment for AEs"

**PBO**	3.40 (0.19, 6.60)	2.39 (-0.66, 5.44)	1.67 (-1.44, 4.77)	0.70 (0.18, 1.21)	2.13 (-0.91, 5.18)	3.14 (0.02, 6.25)
-3.40 (-6.60, -0.19)	**CYC**	-1.01 (-2.00, -0.01)	-1.73 (-6.20, 2.73)	-2.70 (-5.95, 0.55)	-1.26 (-5.68, 3.16)	-0.26 (-4.73, 4.21)
-2.39 (-5.44, 0.66)	1.01 (0.01, 2.00)	**MMF**	-0.73 (-5.08, 3.63)	-1.69 (-4.78, 1.40)	-0.26 (-4.56, 4.05)	0.74 (-3.61, 5.10)
-1.67 (-4.77, 1.44)	1.73 (-2.73, 6.20)	0.73 (-3.63, 5.08)	**CYCAZA**	-0.97 (-4.12, 2.18)	0.47 (-3.88, 4.82)	1.47 (-2.93, 5.87)
-0.70 (-1.21, -0.18)	2.70 (-0.55, 5.95)	1.69 (-1.40, 4.78)	0.97 (-2.18, 4.12)	**NTD**	1.44 (-1.65, 4.52)	2.44 (-0.72, 5.60)
-2.13 (-5.18, 0.91)	1.26 (-3.16, 5.68)	0.26 (-4.05, 4.56)	-0.47 (-4.82, 3.88)	-1.44 (-4.52, 1.65)	**PFD**	1.00 (-3.36, 5.36)
-3.14 (-6.25, -0.02)	0.26 (-4.21, 4.73)	-0.74 (-5.10, 3.61)	-1.47 (-5.87, 2.93)	-2.44 (-5.60, 0.72)	-1.00 (-5.36, 3.36)	**POMA**

4.5 League table "deaths"

**PBO**	-0.02 (-1.20, 1.17)	-0.99 (-2.63, 0.65)	-0.02 (-3.18, 3.15)	-0.02 (-3.07, 3.04)	0.11 (-0.80, 1.03)	0.00 (-3.98, 3.98)	0.30 (-3.72, 4.32)
0.02 (-1.17, 1.20)	**CYC**	-0.97 (-2.11, 0.17)	0.00 (-2.94, 2.94)	0.0 0 (-2.82, 2.82)	0.13 (-1.37, 1.62)	0.02 (-4.13, 4.16)	0.32 (-3.87, 4.51)
0.99 (-0.65, 2.63)	0.97 (-0.17, 2.11)	**MMF**	0.97 (-2.18, 4.12)	0.97 (-2.07, 4.01)	1.10 (-0.78, 2.98)	0.99 (-3.31, 5.29)	1.29 (-3.05, 5.63)
0.02 (-3.15, 3.18)	-0.00 (-2.94, 2.94)	-0.97 (-4.12, 2.18)	**CYCPRED**	-0.00 (-4.07, 4.07)	0.13 (-3.17, 3.43)	0.02 (-5.07, 5.10)	0.32 (-4.80, 5.44)
0.02 (-3.04, 3.07)	0.00 (-2.82, 2.82)	-0.97 (-4.01, 2.07)	0.00 (-4.07, 4.07)	**RTX**	0.13 (-3.06, 3.32)	0.02 (-5.00, 5.03)	0.32 (-4.73, 5.37)
-0.11 (-1.03, 0.80)	-0.13 (-1.62, 1.37)	-1.10 (-2.98, 0.78)	-0.13 (-3.43, 3.17)	-0.13 (-3.32, 3.06)	**NTD**	-0.11 (-4.19, 3.97)	0.19 (-3.93, 4.31)
0.00 (-3.98, 3.98)	-0.02 (-4.16, 4.13)	-0.99 (-5.29, 3.31)	-0.02 (-5.10, 5.07)	-0.02 (-5.03, 5.00)	0.11 (-3.97, 4.19)	**PFD**	0.30 (-5.35, 5.96)
-0.30 (-4.32, 3.72)	-0.32 (-4.51, 3.87)	-1.29 (-5.63, 3.05)	-0.32 (-5.44, 4.80)	-0.32 (-5.37, 4.73)	-0.19 (-4.31, 3.93)	-0.30 (-5.96, 5.35)	**POMA**

Values are SMDs (95% CI) or logORs (95% CI) in the column-defining treatment compared with the row-defining treatment.
Vales in blue cells are significant

5. Netweight

5.1 Netweight "change in FVC % predicted"

Direct comparisons in the network

Network meta-analysis estimates	1vs2	1vs3	1vs5	1vs7	1vs8	2vs3	2vs4	2vs6
Mixed estimates								
1vs2	74.9	12.5				12.5		
1vs3	40.0	20.0				40.0		
1vs5			100.0					
1vs7				100.0				
1vs8					100.0			
2vs3	16.0	16.0				68.0		
2vs4							100.0	
2vs6								100.0
Indirect estimates								
1vs4	40.0	6.7				6.7	46.7	
1vs6	40.0	6.7				6.7		46.7
2vs5	40.0	6.7	46.7			6.7		
2vs7	40.0	6.7		46.7		6.7		
2vs8	40.0	6.7			46.7	6.7		
3vs4	8.7	8.7				37.0	45.7	
3vs5	25.0	12.5	37.5			25.0		
3vs6	8.7	8.7				37.0		45.7
3vs7	25.0	12.5		37.5		25.0		
3vs8	25.0	12.5			37.5	25.0		
4vs5	27.2	4.6	31.8			4.6	31.8	
4vs6							50.0	50.0
4vs7	27.2	4.6		31.8		4.6	31.8	
4vs8	27.2	4.6			31.8	4.6	31.8	
5vs6	27.2	4.6	31.8			4.6		31.8
5vs7			50.0	50.0				
5vs8			50.0		50.0			
6vs7	27.2	4.6		31.8		4.6		31.8
6vs8	27.2	4.6			31.8	4.6		31.8
7vs8				50.0	50.0			
**Entire network**	22.9	6.1	11.9	11.9	11.9	11.6	11.9	11.9
**Included studies**	1	1	1	1	1	1	1	1

1 PBO, 2 CYC, 3 MMF, 4 CYCPRED, 5 CYCAZA, 6 RTX, 7 PFD, 8 POMA

5.2 Netweight "change in DLCO % predicted"

Direct comparisons in the network

Network meta-analysis estimates	1vs2	1vs3	1vs5	1vs6	2vs3	2vs4
Mixed estimates						
1vs2	73.9	13.0			13.0	
1vs3	39.3	21.3			39.3	
1vs5			100.0			
1vs6				100.0		
2vs3	16.8	16.8			66.5	
2vs4						100.0
Indirect estimates						
1vs4	39.5	7.0			7.0	46.5
2vs5	39.5	7.0	46.5		7.0	
2vs6	39.5	7.0		46.5	7.0	
3vs4	9.1	9.2			36.3	45.4
3vs5	24.5	13.3	37.8		24.5	
3vs6	24.5	13.3		37.8	24.5	
4vs5	27.0	4.8	31.7		4.8	31.7
4vs6	27.0	4.8		31.8	4.8	31.7
5vs6			50.0	50.0		
**Entire network**	25.5	8.1	17.1	17.1	15.2	17.1
**Included studies**	1	1	1	1	1	1

1 PBO, 2 CYC, 3 MMF, 4 CYCPRED, 5 CYCAZA, 6 NTD

5.3 Netweight "number of patients with SAEs"

Direct comparisons in the network

Network meta-analysis estimates		1-2	1-3	1-4	1-5	2-3
Mixed estimates						
	1-2	95.2	2.4			2.4
	1-3	45.1	9.9			45.1
	1-4			100.0		
	1-5				100.0	
	2-3	2.5	2.5			95.0
Indirect estimates						
	2-4	47.6	1.6	49.2		1.6
	2-5	47.6	1.6		49.2	1.6
	3-4	30.0	4.9	35.0		30.0
	3-5	30.0	4.9		35.0	30.0
	4-5			50.0	50.0	
**Entire network**		29.8	2.8	23.4	23.4	20.6
**Included studies**		1	1	1	1	1

1 PBO, 2 CYC, 3 MMF, 4 NTD, 5 POMA

5.4 Netweight "number of patients discontinuing treatment for AEs"

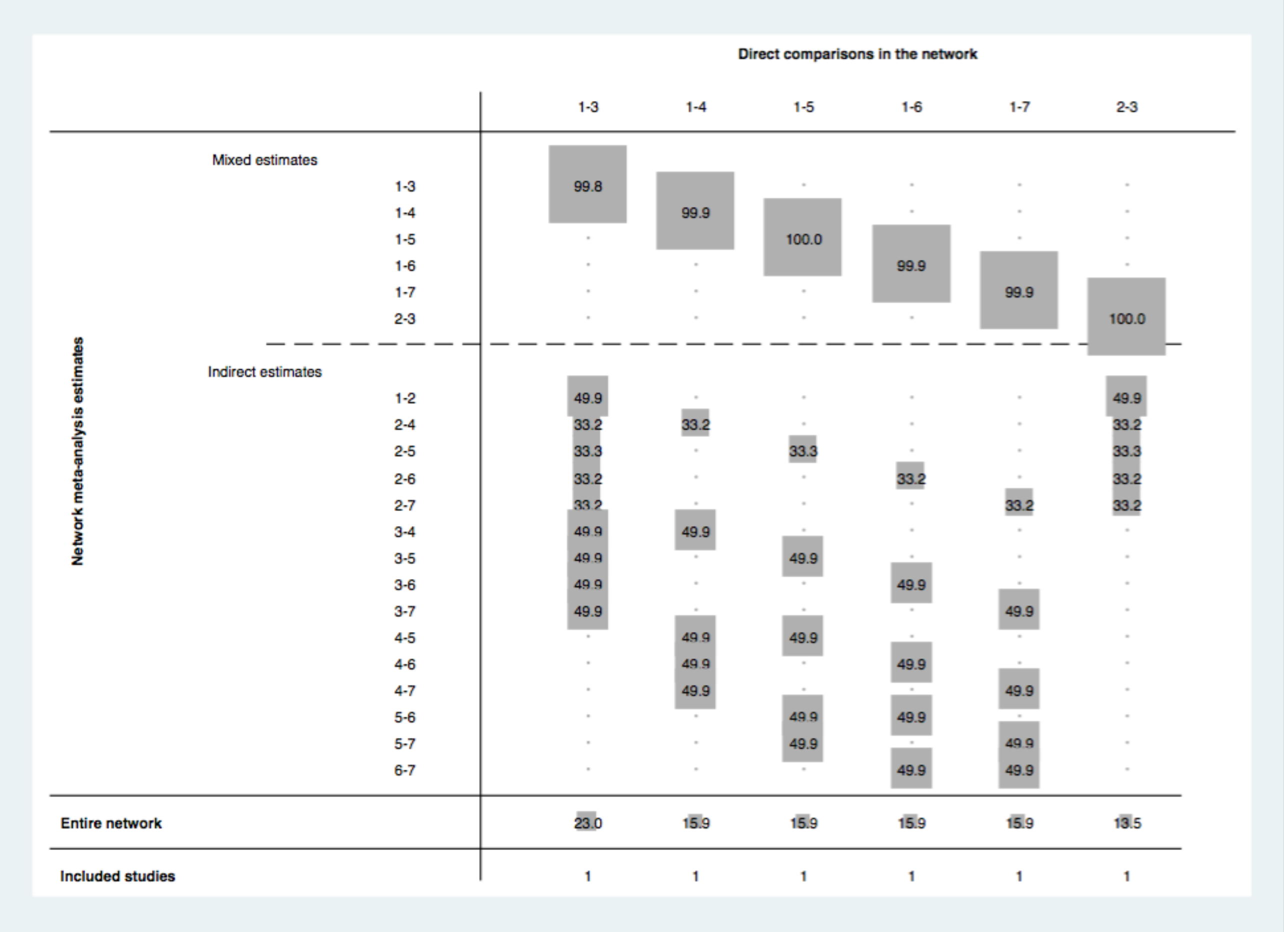

1 PBO, 2 CYC, 3 MMF, 4 CYCAZA, 5 NTD, 6 PFD, 7 POMA

5.5 Netweight "Deaths"

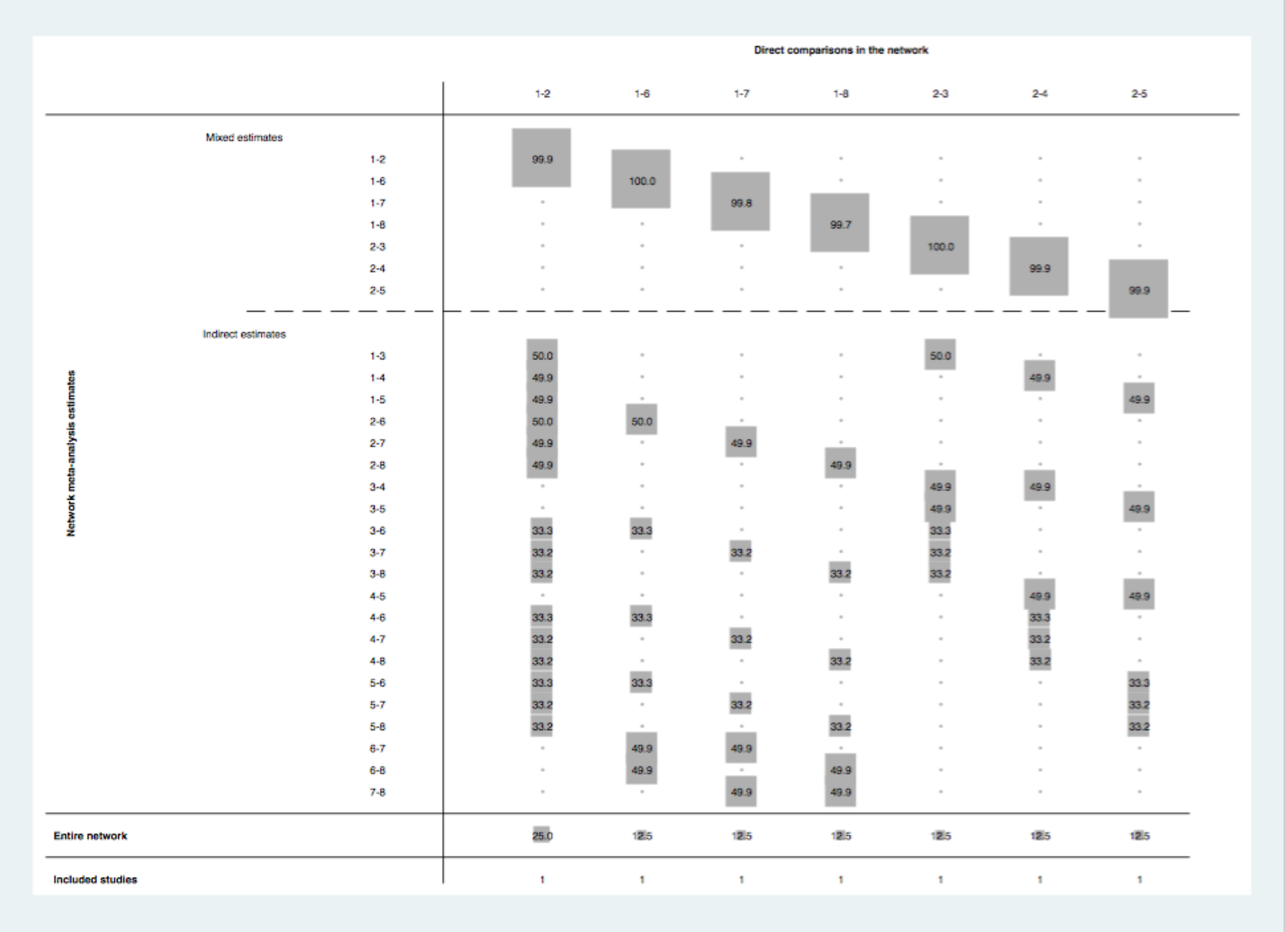

6. Evaluation of inconsistency

6.1 Change in "FVC % of predicted"

6.1.1 Loop-specific heterogeneity estimate

Loop	IF	seIF	z_value	p_value	CI_95	Loop_Heterog_tau2
1 2 3	0.764	0.412	1.852	0.064	(0.00,1.57)	0.000

6.1.2 Node-splitting approach

```
. network sidesplit PBO CYC
```

	Coef.	Std. Err.	z	P>|z|	[95% Conf. Interval]	
direct	.2066582	.1707027	1.21	0.226	-.1279131	.5412294
indirect	-.5568957	.4045283	-1.38	0.169	-1.349757	.2359653
difference	.7635538	.4264319	1.79	0.073	-.0722373	1.599345

```
. network sidesplit PBO MMF
```

	Coef.	Std. Err.	z	P>|z|	[95% Conf. Interval]	
direct	-.7668896	.3258537	-2.35	0.019	-1.405551	-.1282282
indirect	-.0033079	.2666092	-0.01	0.990	-.5258523	.5192364
difference	-.7635816	.4160166	-1.84	0.066	-1.578959	.0517958

```
. network sidesplit MMF CYC
```

	Coef.	Std. Err.	z	P>|z|	[95% Conf. Interval]	
direct	.2099653	.193411	1.09	0.278	-.1691133	.5890438
indirect	.9735494	.378602	2.57	0.010	.2315031	1.715596
difference	-.7635842	.4310735	-1.77	0.077	-1.608473	.0813043

6.1.3 Design-by-treatment test

chi2(1)= 3.43
Prob>chi2 = 0.0640

6.2 Change in "DLCO % of predicted"

6.2.1 Loop-specific heterogeneity estimate

Loop	IF	seIF	z_value	p_value	CI_95	Loop_Heterog_tau2
1 2 3	0.049	0.403	0.120	0.904	(0.00,0.84)	0.000

6.2.2 Node-splitting approach

```
. network sidesplit PBO CYC
```

	Coef.	Std. Err.	z	P>|z|	[95% Conf.	Interval]
direct	-.0758041	.1691187	-0.45	0.654	-.4072707	.2556625
indirect	-.1243596	.3685008	-0.34	0.736	-.8466078	.5978886
difference	.0485555	.4031122	0.12	0.904	-.7415299	.8386408

```
. network sidesplit PBO MMF
```

	Coef.	Std. Err.	z	P>|z|	[95% Conf.	Interval]
direct	.109137	.3138095	0.35	0.728	-.5059184	.7241924
indirect	.1577	.2633696	0.60	0.549	-.358495	.673895
difference	-.048563	.4052704	-0.12	0.905	-.8428783	.7457523

```
. network sidesplit MMF CYC
```

	Coef.	Std. Err.	z	P>|z|	[95% Conf.	Interval]
direct	-.2335031	.2015741	-1.16	0.247	-.628581	.1615749
indirect	-.184942	.4330839	-0.43	0.669	-1.033771	.6638869
difference	-.0485611	.5077231	-0.10	0.924	-1.04368	.9465579

6.2.3 Design-by-treatment test

```
chi2(1)= 0.01
Prob>chi2 = 0.9041
```

6.3 Number of patients with SAEs

6.3.1 Loop-specific heterogeneity estimate

Loop	IF	seIF	z_value	p_value	CI_95	Loop_Heterog_tau2
PBO CYC MMF	0.803	1.758	0.457	0.648	(0.00,4.25)	0.000

6.3.2 Node-splitting approach

. network sidesplit PBO CYC

	Coef.	Std. Err.	z	P>|z|	[95% Conf.	Interval]
direct	.2782039	.3866726	0.72	0.472	-.4796606	1.036068
indirect	1.080837	1.753839	0.62	0.538	-2.356625	4.518299
difference	-.8026333	1.792491	-0.45	0.654	-4.31585	2.710584

. network sidesplit PBO MMF

	Coef.	Std. Err.	z	P>|z|	[95% Conf.	Interval]
direct	1.394878	1.669424	0.84	0.403	-1.877132	4.666888
indirect	.5921924	.5590786	1.06	0.289	-.5035814	1.687966
difference	.8026857	1.760171	0.46	0.648	-2.647186	4.252558

. network sidesplit MMF CYC

	Coef.	Std. Err.	z	P>|z|	[95% Conf.	Interval]
direct	-.3139936	.3957663	-0.79	0.428	-1.089681	.461694
indirect	-1.116665	2.071355	-0.54	0.590	-5.176445	2.943116
difference	.8026711	2.127817	0.38	0.706	-3.367773	4.973115

6.3.3 Design-by-treatment test

chi2(1)= 0.21
Prob>chi2=0.6480

7. Quality ratings

7.1 GRADE "change in FVC % of predicted".

	Direct evidence		Indirect evidence		NMA	
**Comparison**	**SMD (95%CI)**	**Quality of evidence Reason for downgrading**	**SMD (95%CI)**	**Quality of evidence Reason for downgrading**	**SMD (95%CI)**	**Quality of evidence**
Cyclophosphamide vs Placebo	0.20 (-0.12, 0.54)	⊕⊕OO ***low*** Imprecise estimate (-2)	-0.55 (-1.34, 0.23)	⊕OOO ***very low*** Study limitations (-1) Imprecise estimate (-2) Indirectness (-1)	0.08 (-0.22,0.38)	⊕⊕OO ***low***
Mycophenolate vs Placebo	-0.76 (-1.40, -0.12)	⊕⊕OO ***low*** Study limitations (-1) Indirectness (-1)	-0.003 (-0.52, 0.51)	⊕⊕OO ***low*** Imprecise estimate (-2)	-0.29 (-0.69,0.10)	⊕⊕OO ***low***
CYCPRED vs Placebo	-	-	0.23 (-0.74,1.20)	⊕OOO ***very low*** Study limitations (-1) Imprecise estimate (-2)	0.23 (-0.74,1.20)	⊕OOO ***very low***
CYCAZA vs Placebo	0.51 (-0.14,1.17)	⊕OOO ***very low*** Study limitations (-1) Imprecise estimate (-2) Indirectness (-1)	Not estimable	Not estimable	0.51 (-0.14,1.17)	⊕OOO ***very low***
Rituximab vs Placebo	-	-	1.00 (0.39,1.61)	⊕⊕OO ***low*** Study limitations (-1) Indirectness (-1)	1.00 (0.39,1.61)	⊕⊕OO ***low***
Pirfenidone vs Placebo	0.32 (-0.36,1.00)	⊕OOO ***very low*** Imprecise estimate (-2) Indirectness (-1)	Not estimable	Not estimable	0.32 (-0.36,1.00)	⊕OOO ***very low***
Pomalidomide vs Placebo	-0.50 (-1.43,0.43)	⊕OOO ***very low*** Study limitations (-1) Imprecise estimate (-2)	Not estimable	Not estimable	-0.50 (-1.43,0.43)	⊕OOO ***very low***
Cyclophosphamide vs Mycophenolate	0.20 (-0.16, 0.58)	⊕⊕OO ***low*** Imprecise estimate (-2)	0.97 (0.23, 1.71)	⊕⊕OO ***low*** Study limitations (-1) Indirectness (-1)	0.380 (0.04,0.71)	⊕⊕OO ***low***
Cyclophosphamide vs CYCAZA	-	-	-0.43 (-1.15,0.29)	⊕OOO ***very low*** Study limitations (-1) Imprecise estimate (-2)	-0.43 (-1.15,0.29)	⊕OOO ***very low***
Cyclophosphamide vs CYCPRED	-0.15 (-1.07,0.78)	⊕OOO ***very low*** Study limitations (-1)	Not estimable	Not estimable	-0.15 (-1.07,0.78)	⊕OOO ***very low***

		Imprecise estimate (-2)				
Cyclophosphamide vs Rituximab	-0.92<br>(-1.45,-0.39)	⊕⊕OO ***low***<br>Study limitations (-1)<br>Indirectness (-1)	Not estimable	Not estimable	-0.92<br>(-1.45,-0.39)	⊕⊕OO ***low***
Cyclophosphamide vs Pirfenidone	-	-	-0.24<br>(-0.98,0.50)	⊕OOO ***very low***<br>Imprecise estimate (-2)<br>Indirectness (-1)	-0.24<br>(-0.98,0.50)	⊕OOO ***very low***
Cyclophosphamide vs Pomalidomide	-	-	0.58<br>(-0.39,1.56)	⊕OOO ***very low***<br>Study limitations (-1)<br>Imprecise estimate (-2)	0.58<br>(-0.39,1.56)	⊕OOO ***very low***
Mycophenolate vs CYCAZA	-	-	-0.81<br>(-1.57,-0.04)	⊕⊕OO ***low***<br>Study limitations (-1)<br>Indirectness (-1)	-0.81<br>(-1.57,-0.04)	⊕⊕OO ***low***
Mycophenolate vs CYCPRED	-	-	-0.52<br>(-1.51,0.46)	⊕OOO ***very low***<br>Study limitations (-1)<br>Imprecise estimate (-2)	-0.52<br>(-1.51,0.46)	⊕OOO ***very low***
Mycophenolate vs Rituximab	-	-	-1.29<br>(-1.92,-0.67)	⊕⊕OO ***low***<br>Study limitations (-1)<br>Indirectness (-1)	-1.29<br>(-1.92,-0.67)	⊕⊕OO ***low***
Mycophenolate vs Pirfenidone	-	-	-0.61<br>(-1.39,0.17)	⊕OOO ***very low***<br>Study limitations (-1)<br>Imprecise estimate (-2)<br>Indirectness (-1)	-0.61<br>(-1.39,0.17)	⊕OOO ***very low***
Mycophenolate vs Pomalidomide	-	-	0.21<br>(-0.80,1.21)	⊕OOO ***very low***<br>Study limitations (-1)<br>Imprecise estimate (-2)<br>Indirectness (-1)	0.21<br>(-0.80,1.21)	⊕OOO ***very low***
CYCAZA vs CYCPRED	-	-	0.28<br>(-0.89,1.46)	⊕OOO ***very low***<br>Study limitations (-1)<br>Imprecise estimate (-2)<br>Indirectness (-1)	0.28<br>(-0.89,1.46)	⊕OOO ***very low***
CYCAZA vs Rituximab	-	-	-0.49<br>(-1.39,0.41)	⊕OOO ***very low***<br>Study limitations (-1)<br>Imprecise estimate (-2)<br>Indirectness (-1)	-0.49<br>(-1.39,0.41)	⊕OOO ***very low***
CYCAZA Pirfenidone	-	-	0.20<br>(-0.75,1.14)	⊕OOO ***very low***<br>Study limitations (-1)<br>Imprecise estimate (-2)<br>Indirectness (-1)	0.20<br>(-0.75,1.14)	⊕OOO ***very low***

CYCAZA vs Pomalidomide	-	-	1.01<br>(-0.12,2.15)	⊕OOO ***very low***<br>Study limitations (-1)<br>Imprecise estimate (-2)	1.01<br>(-0.12,2.15)	⊕OOO ***very low***
CYCPRED vs Rituximab	-	-	-0.77<br>(-1.84,0.30)	⊕OOO ***very low***<br>Study limitations (-1)<br>Imprecise estimate (-2)	-0.77<br>(-1.84,0.30)	⊕OOO ***very low***
CYCPRED vs Pirfenidone	-	-	-0.09<br>(-1.27,1.10)	⊕OOO ***very low***<br>Study limitations (-1)<br>Imprecise estimate (-2)	-0.09<br>(-1.27,1.10)	⊕OOO ***very low***
CYCPRED vs Pomalidomide	-	-	0.73<br>(-0.61,2.07)	⊕OOO ***very low***<br>Study limitations (-1)<br>Imprecise estimate (-2)	0.73<br>(-0.61,2.07)	⊕OOO ***very low***
Rituximab vs Pirfenidone	-	-	0.68<br>(-0.23,1.60)	⊕OOO ***very low***<br>Study limitations (-1)<br>Imprecise estimate (2)<br>Indirectness (-1)	0.68<br>(-0.23,1.60)	⊕OOO ***very low***
Rituximab vs Pomalidomide	-	-	1.50<br>(0.39,2.61)	⊕⊕OO ***low***<br>Study limitations (-1)<br>Indirectness (-1)	1.50<br>(0.39,2.61)	⊕⊕OO ***low***
Pirfenidone vs Pomalidomide	-	-	0.82<br>(-0.33,1.97)	⊕OOO ***very low***<br>Study limitations (-1)<br>Imprecise estimate (-2)<br>Indirectness (-1)	0.82<br>(-0.33,1.97)	⊕OOO ***very low***

7.2 GRADE "change in DLCO % of predicted".

Comparison	Direct evidence SMD (95%CI)	Direct evidence Quality of evidence Reason for downgrading	Indirect evidence SMD (95%CI)	Indirect evidence Quality of evidence Reason for downgrading	NMA SMD (95%CI)	NMA Quality of evidence
Cyclophosphamide vs Placebo	-0.07 (-0.40, 0.25)	⊕⊕OO ***low*** Imprecise estimate (-2)	-0.12 (-0.84, 0.59)	⊕OOO ***very low*** Study limitations (-1) Imprecise estimate (-2) Indirectness (-1)	-0.08 (-0.38,0.21)	⊕⊕OO ***low***
Mycophenolate vs Placebo	0.10 (-0.50, 0.72)	⊕OOO ***very low*** Study limitations (-1) Imprecise estimate (-2) Indirectness (-1)	0.15 (-0.35, 0.67)	⊕⊕OO ***low*** Imprecise estimate (-2)	0.14 (-0.25,0.53)	⊕⊕OO ***low***
CYCPRED vs Placebo	-	-	0.90 (-0.44,2.24)	⊕OOO ***very low*** Study limitations (-1) Imprecise estimate (-2)	0.90 (-0.44,2.24)	⊕OOO ***very low***
CYCAZA vs Placebo	-0.01 (-0.66, 0.63)	⊕OOO ***very low*** Study limitations (-1) Imprecise estimate (-2) Indirectness (-1)	Not estimable	Not estimable	-0.01 (-0.66,0.63)	⊕OOO ***very low***
Nintedanib vs Placebo	-0.05 (-0.21,0.12)	⊕⊕OO ***low*** Imprecise estimate (-2)	Not estimable	Not estimable	-0.05 (-0.21,0.12)	⊕⊕OO ***low*** Imprecise estimate (-2)
Cyclophosphamide vs Mycophenolate	-0.22 (-0.55,0.11)	⊕⊕OO ***low*** Imprecise estimate (-2)	-0.18 (-1.03, 0.66)	⊕OOO ***very low*** Study limitations (-1) Imprecise estimate (-2) Indirectness (-1)	-0.22 (-0.55,0.11)	⊕⊕OO ***low***
Cyclophosphamide vs CYCAZA	-	-	-0.07 (-0.78,0.64)	⊕OOO ***very low*** Study limitations (-1) Imprecise estimate (-2)	-0.07 (-0.78, 0.64)	⊕OOO ***very low***
Cyclophosphamide vs CYCPRED	-0.98 (-2.29,0.32)	⊕OOO ***very low*** Study limitations (-1) Imprecise estimate (-2)	Not estimable	Not estimable	-0.98 (-2.29,0.32)	⊕OOO ***very low***
Cyclophosphamide vs Nintedanib	-	-	-0.04 (-0.37,0.30)	⊕⊕OO ***low*** Imprecise estimate (-2)	-0.04 (-0.37,0.30)	⊕⊕OO ***low*** Imprecise estimate (-2)
Mycophenolate vs CYCAZA	-	-	0.15 (-0.60,0.90)	⊕OOO ***very low*** Study limitations (-1) Imprecise estimate (-2) Indirectness (-1)	0.15 (-0.60,0.90)	⊕OOO ***very low***
Mycophenolate vs CYCPRED	-	-	-0.76	⊕OOO ***very low***	-0.76	⊕OOO ***very low***

			(-2.11,0.59)	Study limitations (-1)<br>Imprecise estimate (-2)	(-2.11,0.59)	
Mycophenolate vs Nintedanib	-	-	0.19<br>(-0.23,0.61)	⨁OOO ***very low***<br>Study limitations (-1)<br>Imprecise estimate (-2)<br>Indirectness (-1)	0.19<br>(-0.23,0.61)	⨁OOO ***very low***
CYCAZA vs CYCPRED	-	-	-0.91<br>(-2.40,0.57)	⨁OOO ***very low***<br>Study limitations (-1)<br>Imprecise estimate (-2)<br>Indirectness (-1)	-0.91<br>(-2.40,0.57)	⨁OOO ***very low***
CYCAZA vs Nintedanib	-	-	0.04<br>(-0.63,0.70)	⨁OOO ***very low***<br>Study limitations (-1)<br>Imprecise estimate (-2)	0.87<br>(0.20, 1.53)	⨁OOO ***very low***
CYCPRED vs Nintedanib	-	-	0.95<br>(-0.40,2.30)	⨁OOO ***very low***<br>Study limitations (-1)<br>Imprecise estimate (-2)	0.95<br>(-0.40,2.30)	⨁OOO ***very low***

7.3 GRADE "number of patients with SAEs"

Comparison	Direct evidence logOR (95%CI)	Direct evidence Quality of evidence Reason for downgrading	Indirect evidence logOR (95%CI)	Indirect evidence Quality of evidence Reason for downgrading	NMA logOR (95%CI)	NMA Quality of evidence
Cyclophosphamide vs Placebo	0.27 (-0.47, 1.03)	⊕⊕OO ***low*** Imprecise estimate (-2)	1.08 (-2.35, 4.51)	⊕OOO ***very low*** Study limitations (-1) Imprecise estimate (-2) Indirectness (-1)	0.32 (-0.42, 1.06)	⊕⊕OO ***low***
Mycophenolate vs Placebo	1.39 (-1.87, 4.66)	⊕OOO ***very low*** Study limitations (-1) Imprecise estimate (-2) Indirectness (-1)	0.59 (-0.50, 1.68)	⊕⊕OO ***low*** Imprecise estimate (-2)	0.67 (-0.36, 1.70)	⊕⊕OO ***low***
Nintedanib vs Placebo	0.14 (-0.25, 0.53)	⊕⊕OO ***low*** Imprecise estimate (-2)	Not estimable	Not estimable	0.14 (-0.25, 0.53)	⊕⊕OO ***low***
Pomalidomide vs Placebo	2.30 (-0.18, 4.78)	⊕OOO ***very low*** Study limitations (-1) Imprecise estimate (-2)	Not estimable	Not estimable	2.30 (-0.18, 4.78)	⊕OOO ***very low***
Cyclophosphamide vs Mycophenolate	-0.31 (-1.08, 0.46)	⊕⊕OO ***low*** Imprecise estimate (-2)	-1.11 (-5.17, 2.94)	⊕OOO ***very low*** Study limitations (-1) Imprecise estimate (-2) Indirectness (-1)	-0.35 (-1.11, 0.40)	⊕⊕OO ***low***
Cyclophosphamide vs Nintedanib	-	-	0.17 (-0.66, 1.01)	⊕OOO ***very low*** Study limitations (-1) Imprecise estimate (-2)	0.17 (-0.66, 1.01)	⊕OOO ***very low***
Cyclophosphamide vs Pomalidomide	-	-	-1.99 (-4.57, 0.60)	⊕OOO ***very low*** Study limitations (-1) Imprecise estimate (-2)	-1.99 (-4.57, 0.60)	⊕OOO ***very low***
Mycophenolate vs Nintedanib			0.53 (-0.57, 1.63)	⊕OOO ***very low*** Study limitations (-1) Imprecise estimate (-2) Indirectness (-1)	0.53 (-0.57, 1.63)	⊕OOO ***very low***
Mycophenolate vs Pomalidomide	-	-	-1.63 (-4.31, 1.05)	⊕OOO ***very low*** Study limitations (-1) Imprecise estimate (-2) Indirectness (-1)	-1.63 (-4.31, 1.05)	⊕OOO ***very low***
Nintedanib vs Pomalidomide	-	-	-2.16 (-4.67, 0.35)	⊕OOO ***very low*** Study limitations (-1) Imprecise estimate (-2)	-2.16 (-4.67, 0.35)	⊕OOO ***very low***

7.4 GRADE "number of patients discontinuing treatment for AEs"

	Direct evidence		Indirect evidence		NMA	
**Comparison**	**logOR (95%CI)**	**Quality of evidence Reason for downgrading**	**logOR (95%CI)**	**Quality of evidence Reason for downgrading**	**logOR (95%CI)**	**Quality of evidence**
Cyclophosphamide vs Placebo	-	-	3.40 (0.19, 6.60)	⨁OOO ***very low*** Study limitations (-1) Imprecise estimate (-1) Indirectness (-1)	3.40 (0.19, 6.60)	⨁OOO ***very low***
Mycophenolate vs Placebo	2.39 (-0.66, 5.44)	⨁OOO ***very low*** Study limitations (-1) Imprecise estimate (-2) Indirectness (-1)	Not estimable	Not estimable	2.39 (-0.66, 5.44)	⨁OOO ***very low***
CYCAZA vs Placebo	1.67 (-1.44, 4.77)	⨁OOO ***very low*** Study limitations (-1) Imprecise estimate (-2) Indirectness (-1)	Not estimable	Not estimable	1.67 (-1.44, 4.77)	⨁OOO ***very low***
Nintedanib vs Placebo	0.70 (0.18, 1.21)	⨁⨁⨁⨁ ***high***	Not estimable	Not estimable	0.70 (0.18, 1.21)	⨁⨁⨁⨁ ***high***
Pirfenidone vs Placebo	2.13 (-0.91, 5.18)	⨁OOO ***very low*** Imprecise estimate (-2) Indirectness (-1)	Not estimable	Not estimable	2.13 (-0.91, 5.18)	⨁OOO ***very low***
Pomalidomide vs Placebo	3.14 (0.02, 6.25)	⨁OOO ***very low*** Study limitations (-1) Imprecise estimate (-2)	Not estimable	Not estimable	3.14 (0.02, 6.25)	⨁OOO ***very low***
Cyclophosphamide vs Mycophenolate	1.01 (0.01, 2.00)	⨁⨁⨁⨁ ***high***	Not estimable	Not estimable	1.01 (0.01, 2.00)	⨁⨁⨁⨁ ***high***
Cyclophosphamide vs CYCAZA	-	--	1.73 (-2.73, 6.20)	⨁OOO ***very low*** Study limitations (-1) Imprecise estimate (-2) Indirectness (-1)	1.73 (-2.73, 6.20)	⨁OOO ***very low***
Cyclophosphamide vs Nintedanib			2.70 (-0.55, 5.95)	⨁OOO ***very low*** Study limitations (-1) Imprecise estimate (-2) Indirectness (-1)	2.70 (-0.55, 5.95)	⨁OOO ***very low***
Cyclophosphamide vs Pirfenidone	-	-	1.26 (-3.16, 5.68)	⨁OOO ***very low*** Study limitations (-1) Imprecise estimate (-2) Indirectness (-1)	1.26 (-3.16, 5.68)	⨁OOO ***very low***

Cyclophosphamide vs Pomalidomide	-	-	0.26<br>(-4.21, 4.73)	⊕OOO ***very low***<br>Study limitations (-1)<br>Imprecise estimate (-2)<br>Indirectness (-1)	0.26<br>(-4.21, 4.73)	⊕OOO ***very low***
Mycophenolate vs CYCAZA	-	-	0.73<br>(-3.63, 5.08)	⊕OOO ***very low***<br>Study limitations (-1)<br>Imprecise estimate (-2)<br>Indirectness (-1)	0.73<br>(-3.63, 5.08)	⊕OOO ***very low***
Mycophenolate vs Nintedanib	-	-	1.69<br>(-1.40, 4.78)	⊕OOO ***very low***<br>Study limitations (-1)<br>Imprecise estimate (-2)<br>Indirectness (-1)	1.69<br>(-1.40, 4.78)	⊕OOO ***very low***
Mycophenolate vs Pirfenidone	-	-	0.26<br>(-4.05, 4.56)	⊕OOO ***very low***<br>Study limitations (-1)<br>Imprecise estimate (-2)<br>Indirectness (-1)	0.26<br>(-4.05, 4.56)	⊕OOO ***very low***
Mycophenolate vs Pomalidomide	-	-	-0.74<br>(-5.10, 3.61)	⊕OOO ***very low***<br>Study limitations (-1)<br>Imprecise estimate (-2)<br>Indirectness (-1)	-0.74<br>(-5.10, 3.61)	⊕OOO ***very low***
CYCAZA vs Nintedanib	-	-	0.97<br>(-2.18, 4.12)	⊕OOO ***very low***<br>Study limitations (-1)<br>Imprecise estimate (-2)	0.97<br>(-2.18, 4.12)	⊕OOO ***very low***
CYCAZA Pirfenidone	-	-	-0.47<br>(-4.82, 3.88)	⊕OOO ***very low***<br>Study limitations (-1)<br>Imprecise estimate (-2)<br>Indirectness (-1)	-0.47<br>(-4.82, 3.88)	⊕OOO ***very low***
CYCAZA vs Pomalidomide	-	-	-1.47<br>(-5.87, 2.93)	⊕OOO ***very low***<br>Study limitations (-1)<br>Imprecise estimate (-2)	-1.47<br>(-5.87, 2.93)	⊕OOO ***very low***
Nintedanib vs Pirfenidone			-1.44<br>(-4.52, 1.65)	⊕⊕OO ***low***<br>Imprecise estimate (-2)	-1.44<br>(-4.52, 1.65)	⊕⊕OO ***low***
Nintedanib vs Pomalidomide	-	-	-2.44<br>(-5.60, 0.72)	⊕⊕OO ***low***<br>Study limitations (-1)<br>Imprecise estimate (-2)	-2.44<br>(-5.60, 0.72)	⊕OOO ***very low***
Pirfenidone vs Pomalidomide	-	-	-1.00<br>(-5.36, 3.36)	⊕OOO ***very low***<br>Study limitations (-1)<br>Imprecise estimate (-2)<br>Indirectness (-1)	-1.00<br>(-5.36, 3.36)	⊕OOO ***very low***

7.5 GRADE "Deaths"

Comparison	Direct evidence logOR (95%CI)	Direct evidence Quality of evidence Reason for downgrading	Indirect evidence logOR (95%CI)	Indirect evidence Quality of evidence Reason for downgrading	NMA logOR (95%CI)	NMA Quality of evidence
Cyclophosphamide vs Placebo	-0.02<br>(-1.20, 1.17)	⨁⨁OO ***low***<br>Imprecise estimate (-2)	Not estimable	Not estimable	-0.02<br>(-1.20, 1.17)	⨁⨁OO ***low***
Mycophenolate vs Placebo	-	-	-0.99<br>(-2.63, 0.65)	⨁⨁OO ***low***<br>Imprecise estimate (-2)	-0.99<br>(-2.63, 0.65)	⨁⨁OO ***low***
CYCPRED vs Placebo	-	-	-0.02<br>(-3.18, 3.15)	⨁⨁OO ***low***<br>Study limitations (-1)<br>Imprecise estimate (-2)	-0.02<br>(-3.18, 3.15)	⨁⨁OO ***low***
Rituximab vs Placebo	-	-	-0.02<br>(-3.07, 3.04)	⨁OOO ***very low***<br>Study limitations (-1)<br>Imprecise estimate (-2)<br>Indirectness (-1)	-0.02<br>(-3.07, 3.04)	⨁OOO ***very low***
Nintedanib vs Placebo	0.11<br>(-0.80, 1.03)	⨁⨁OO ***low***<br>Imprecise estimate (-2)	Not estimable	Not estimable	0.11<br>(-0.80, 1.03)	⨁⨁OO ***low***
Pirfenidone vs Placebo	0.00<br>(-3.98, 3.98)	⨁OOO ***very low***<br>Imprecise estimate (-2)<br>Indirectness (-1)	Not estimable	Not estimable	0.00<br>(-3.98, 3.98)	⨁OOO ***very low***
Pomalidomide vs Placebo	0.30<br>(-3.72, 4.32)	⨁OOO ***very low***<br>Study limitations (-1)<br>Imprecise estimate (-2)	Not estimable	Not estimable	0.30<br>(-3.72, 4.32)	⨁OOO ***very low***
Cyclophosphamide vs Mycophenolate	0.97<br>(-0.17, 2.11)	⨁⨁OO ***low***<br>Imprecise estimate (-2)	Not estimable	Not estimable	0.97<br>(-0.17, 2.11)	⨁⨁OO ***low***
Cyclophosphamide vs CYCPRED	-0.00<br>(-2.94, 2.94)	⨁OOO ***very low***<br>Study limitations (-1)<br>Imprecise estimate (-2)	Not estimable	Not estimable	-0.00<br>(-2.94, 2.94)	⨁OOO ***very low***
Cyclophosphamide vs Rituximab	0.00<br>(-2.82, 2.82)	⨁OOO ***very low***<br>Study limitations (-1)<br>Imprecise estimate (-2)<br>Indirectness (-1)	Not estimable	Not estimable	0.00<br>(-2.82, 2.82)	⨁OOO ***very low***
Cyclophosphamide vs Nintedanib	-	-	-0.13<br>(-1.62, 1.37)	⨁⨁OO ***low***<br>Imprecise estimate (-2)	-0.13<br>(-1.62, 1.37)	⨁⨁OO ***low***
Cyclophosphamide vs Pirfenidone	-	-	-0.02<br>(-4.16, 4.13)	⨁⨁OO ***low***<br>Imprecise estimate (-2)	-0.02<br>(-4.16, 4.13)	⨁⨁OO ***low***
Cyclophosphamide vs Pomalidomide	-	-	-0.32	⨁OOO ***very low***	-0.32	⨁OOO ***very low***

			(-4.51, 3.87)	Study limitations (-1)<br>Imprecise estimate (-2)	(-4.51, 3.87)	
Mycophenolate vs CYCPRED	-	-	-0.97<br>(-4.12, 2.18)	⨁OOO ***very low***<br>Study limitations (-1)<br>Imprecise estimate (-2)	-0.97<br>(-4.12, 2.18)	⨁OOO ***very low***
Mycophenolate vs Rituximab	-	-	-0.97<br>(-4.01, 2.07)	⨁OOO ***very low***<br>Study limitations (-1)<br>Imprecise estimate (-2)<br>Indirectness (-1)	-0.97<br>(-4.01, 2.07)	⨁OOO ***very low***
Mycophenolate vs Nintedanib	-	-	-1.10<br>(-2.98,0.78)	⨁⨁OO ***low***<br>Imprecise estimate (-2)	-1.10<br>(-2.98, 0.78)	⨁⨁OO ***low***
Mycophenolate vs Pirfenidone	-	-	-0.99<br>(-5.29, 3.31)	⨁OOO ***very low***<br>Imprecise estimate (-2)<br>Indirectness (-1)	-0.99<br>(-5.29, 3.31)	⨁OOO ***very low***
Mycophenolate vs Pomalidomide	-	-	-1.29<br>(-5.63, 3.05)	⨁OOO ***very low***<br>Study limitations (-1)<br>Imprecise estimate (-2)<br>Indirectness (-1)	-1.29<br>(-5.63, 3.05)	⨁OOO ***very low***
CYCPRED vs Rituximab	-	-	0.00<br>(-4.07, 4.07)	⨁OOO ***very low***<br>Study limitations (-1)<br>Imprecise estimate (-2)<br>Indirectness (-1)	0.00<br>(-4.07, 4.07)	⨁OOO ***very low***
CYCPRED vs Nintedanib	-	-	-0.13<br>(-3.43, 3.17)	⨁OOO ***very low***<br>Study limitations (-1)<br>Imprecise estimate (-2)	-0.13<br>(-3.43, 3.17)	⨁OOO ***very low***
CYCPRED vs Pirfenidone	-	-	-0.02<br>(-5.10, 5.07)	⨁OOO ***very low***<br>Study limitations (-1)<br>Imprecise estimate (-2)<br>Indirectness (-1)	-0.02<br>(-5.10, 5.07)	⨁OOO ***very low***
CYCPRED vs Pomalidomide	-	-	-0.32<br>(-5.44, 4.80)	⨁OOO ***very low***<br>Study limitations (-1)<br>Imprecise estimate (-2)	-0.32<br>(-5.44, 4.80)	⨁OOO ***very low***
Rituximab vs Nintedanib	-	-	-0.13<br>(-3.32, 3.06)	⨁OOO ***very low***<br>Study limitations (-1)<br>Imprecise estimate (-2)<br>Indirectness (-1)	-0.13<br>(-3.32, 3.06)	⨁OOO ***very low***
Rituximab vs Pirfenidone	-	-	-0.02<br>(-5.03, 5.00)	⨁OOO ***very low***<br>Study limitations (-1)<br>Imprecise estimate (-2)	-0.02<br>(-5.03, 5.00)	⨁OOO ***very low***

				Indirectness (-1)		
Rituximab vs Pomalidomide	-	-	-0.02<br>(-5.03, 5.00)	⊕OOO ***very low***<br>Study limitations (-1)<br>Imprecise estimate (-2)<br>Indirectness (-1)	-0.02<br>(-5.03, 5.00)	⊕OOO ***very low***
Nintedanib vs Pirfenidone	-	-	0.11<br>(-3.97, 4.19)	⊕OOO ***very low***<br>Imprecise estimate (-2)<br>Indirectness (-1)	0.11<br>(-3.97, 4.19)	⊕OOO ***very low***
Nintedanib vs Pomalidomide	-	-	-0.19<br>(-4.31, 3.93)	⊕OOO ***very low***<br>Study limitations (-1)<br>Imprecise estimate (-2)	-0.19<br>(-4.31, 3.93)	⊕OOO ***very low***
Pirfenidone vs Pomalidomide	-	-	-0.30<br>(-5.96, 5.35)	⊕OOO ***very low***<br>Study limitations (-1)<br>Imprecise estimate (-2)<br>Indirectness (-1)	-0.30<br>(-5.96, 5.35)	⊕OOO ***very low***

8. SUCRAs and cumulative probability plots

8.1 SUCRA "change in FVC % of predicted"

Treatment Relative Ranking of Estimated probabilities

Treatment	SUCRA	PrBest	MeanRank
Placebo	37.6	00.0	05.4
Cyclophosphamide	47.7	00.0	04.7
Mycophenolate	13.8	00.0	07.0
CYCPRED	55.1	06.2	04.1
CYCAZA	74.8	12.3	02.8
Rituximab	95.7	76.4	01.3
Pirfenidone	62.8	04.9	03.6
Pomalidomide	12.5	00.3	07.1

Treatment Relative Ranking of Predictive probabilities

Treatment	SUCRA	PrBest	MeanRank
Placebo	37.8	00.0	05.4
Cyclophosphamide	47.5	00.0	04.7
Mycophenolate	13.9	00.0	07.0
CYCPRED	55.0	06.2	04.2
CYCAZA	74.7	12.1	02.8
Rituximab	95.7	76.7	01.3
Pirfenidone	62.8	04.9	03.6
Pomalidomide	12.5	00.2	07.1

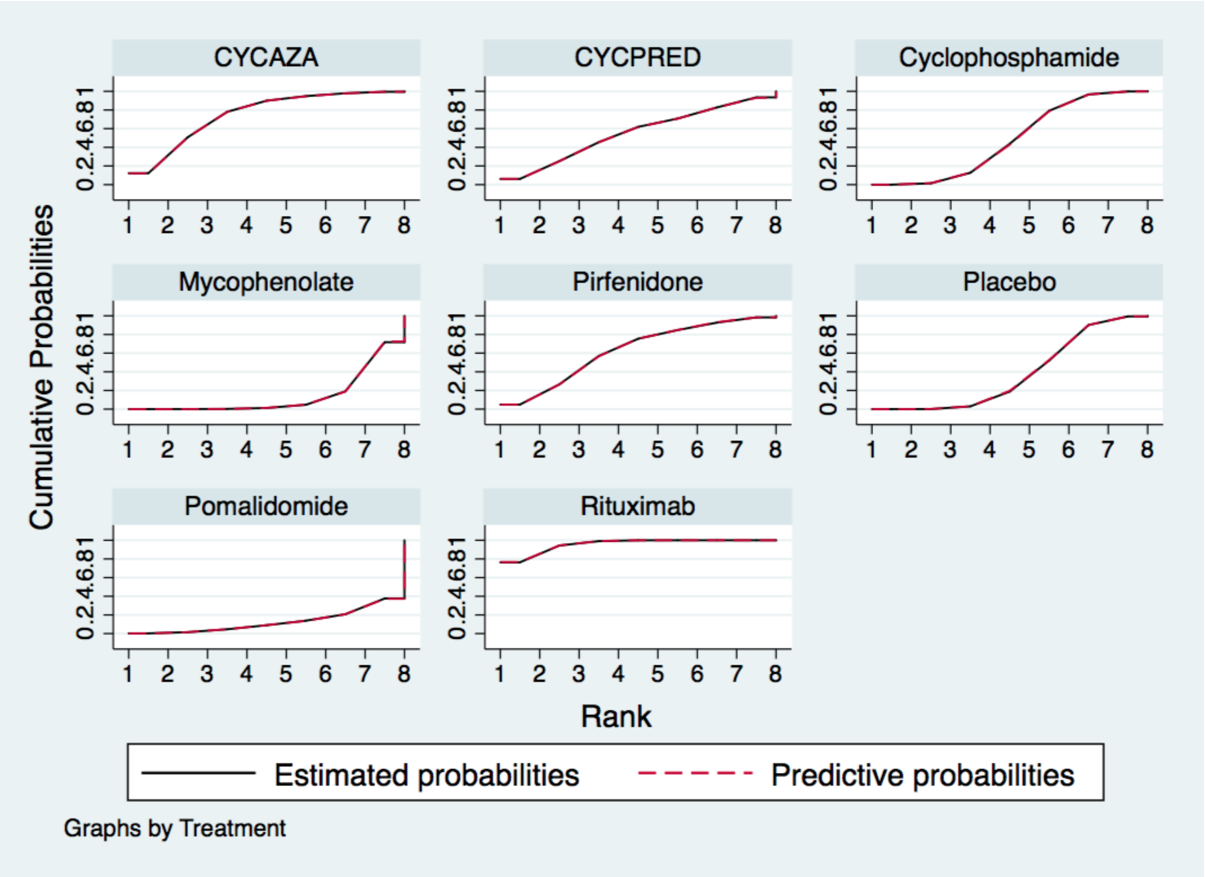

8.2 SUCRA "change in DLCO % of predicted"

Treatment Relative Ranking of Estimated probabilities

Treatment	SUCRA	PrBest	MeanRank
Placebo	45.5	01.0	03.7
Cyclophosphamide	25.9	00.2	04.7
Mycophenolate	65.3	08.6	02.7
CYCPRED	89.8	82.6	01.5
CYCAZA	41.7	06.8	03.9
Nintedanib	31.9	00.8	04.4

Treatment Relative Ranking of Predictive probabilities

Treatment	SUCRA	PrBest	MeanRank
Placebo	45.4	01.1	03.7
Cyclophosphamide	26.2	00.1	04.7
Mycophenolate	65.3	08.5	02.7
CYCPRED	90.2	83.0	01.5
CYCAZA	41.5	06.6	03.9
Nintedanib	31.4	00.7	04.4

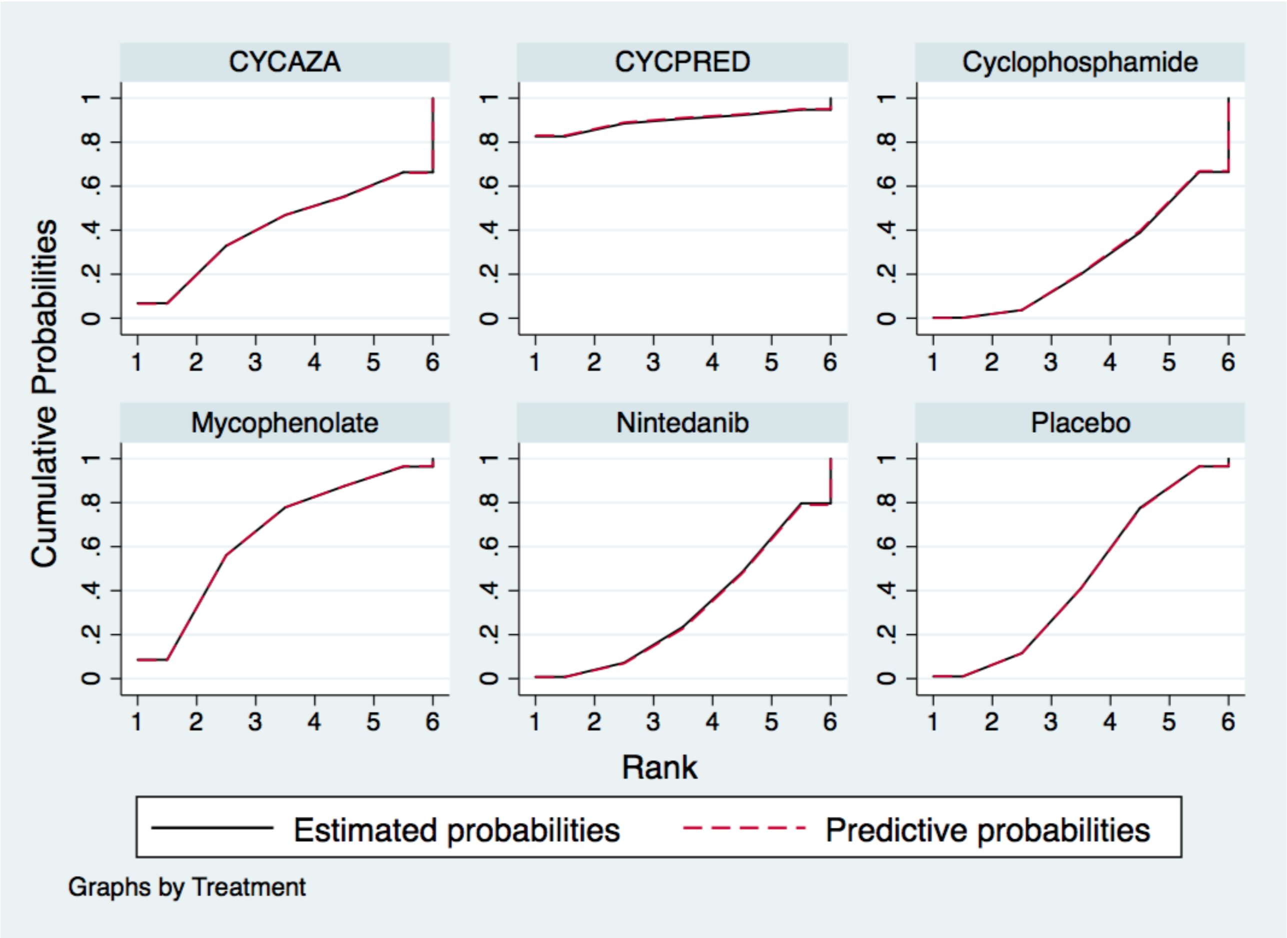

8.3 SUCRA "number of patients with SAEs"

Treatment	SUCRA	PrBest	MeanRank
Placebo	85.7	56.5	01.6
Cyclophosphamide	57.5	15.4	02.7
Mycophenolate	33.2	05.9	03.7
Nintedanib	67.0	19.3	02.3
Pomalidomide	06.5	03.0	04.7

Treatment Relative Ranking of Predictive probabilities

Treatment	SUCRA	PrBest	MeanRank
Placebo	86.1	57.7	01.6
Cyclophosphamide	57.3	14.7	02.7
Mycophenolate	33.0	05.7	03.7
Nintedanib	66.9	18.7	02.3
Pomalidomide	06.6	03.2	04.7

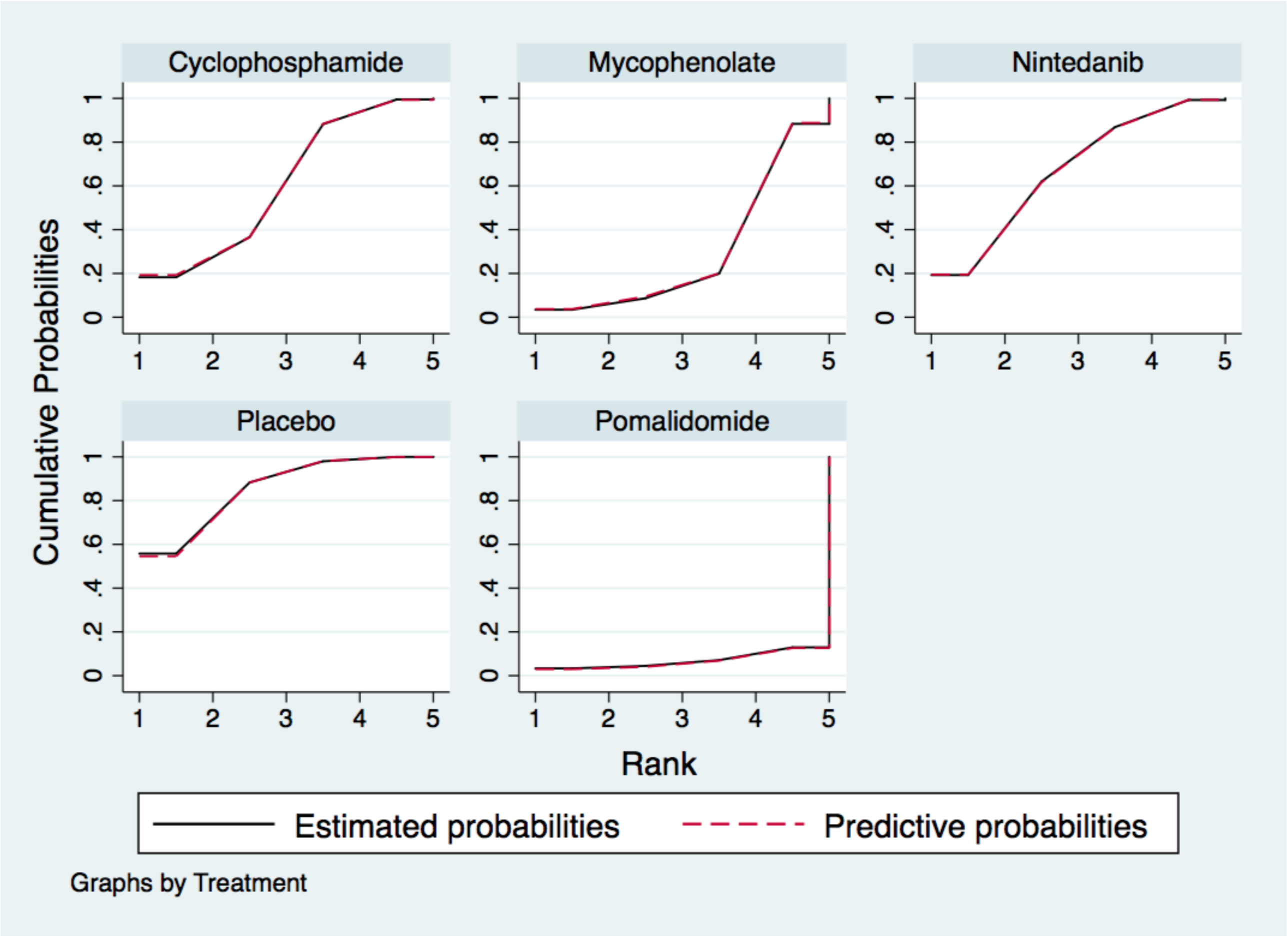

8.4 SUCRA "number of patients discontinuing treatment for AEs"

Treatment Relative Ranking of Estimated probabilities

Treatment	SUCRA	PrBest	MeanRank
Placebo	94.2	70.7	01.3
Cyclophosphamide	17.8	00.1	05.9
Mycophenolate	44.0	05.3	04.4
CYCAZA	53.0	13.8	03.8
Nintedanib	71.5	00.2	02.7
Pirfenidone	43.4	08.0	04.4
Pomalidomide	26.1	01.9	05.4

Treatment Relative Ranking of Predictive probabilities

Treatment	SUCRA	PrBest	MeanRank
Placebo	94.3	71.1	01.3
Cyclophosphamide	17.8	00.2	05.9
Mycophenolate	44.1	05.2	04.4
CYCAZA	52.3	13.6	03.9
Nintedanib	71.5	00.3	02.7
Pirfenidone	43.8	07.6	04.4
Pomalidomide	26.2	02.0	05.4

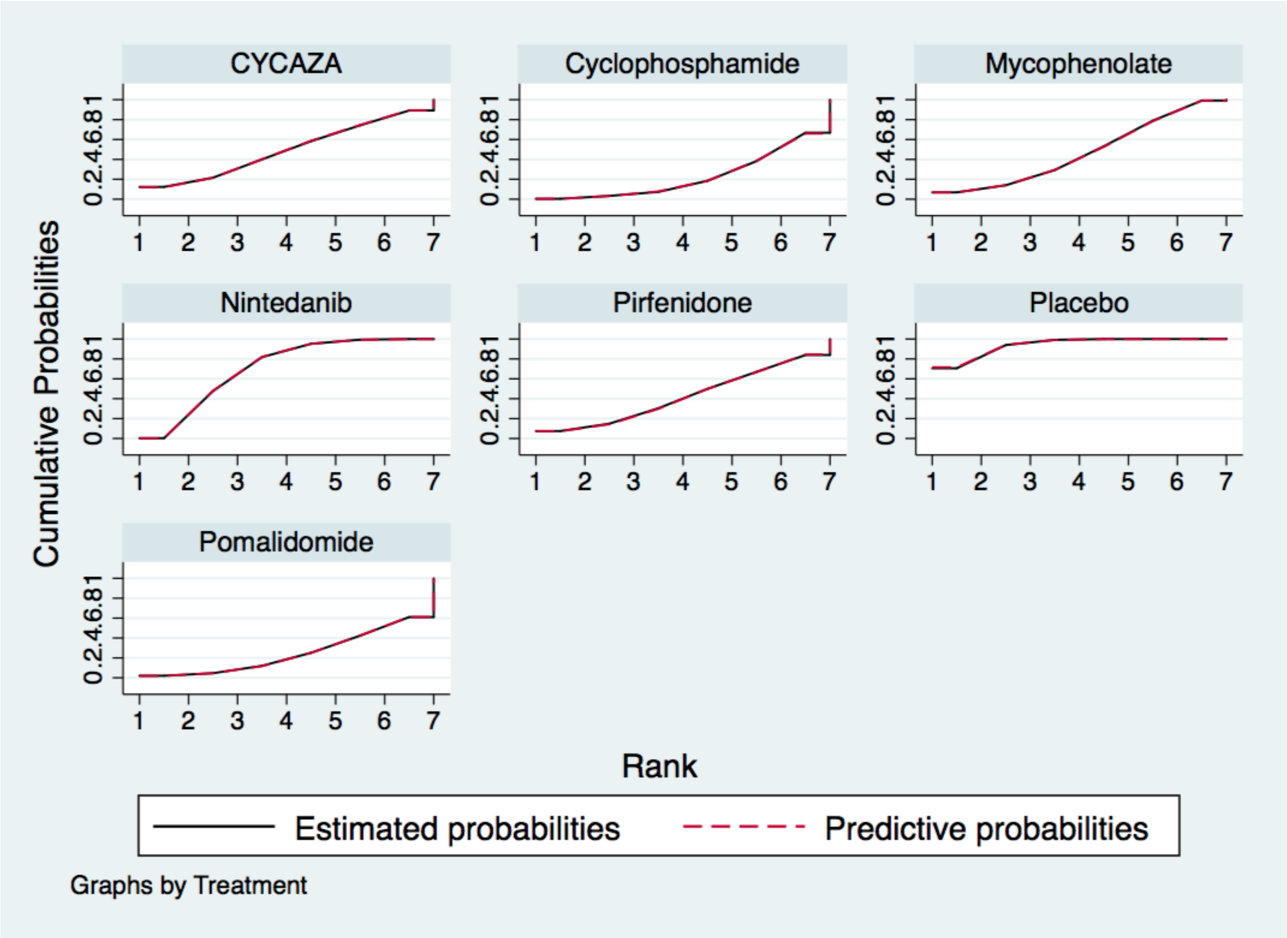

8.5 SUCRA "Deaths"

Treatment Relative Ranking of Estimated probabilities

Treatment	SUCRA	PrBest	MeanRank
Placebo	46.4	00.9	04.8
Cyclophosphamide	45.4	00.2	04.8
Mycophenolate	79.5	28.3	02.4
CYCPRED	48.1	14.7	04.6
Rituximab	47.7	14.3	04.7
Nintedanib	41.9	02.0	05.1
Pirfenidone	48.5	22.3	04.6
Pomalidomide	42.7	17.5	05.0

Treatment Relative Ranking of Predictive probabilities

Treatment	SUCRA	PrBest	MeanRank
Placebo	46.0	00.8	04.8
Cyclophosphamide	45.7	00.2	04.8
Mycophenolate	79.7	28.3	02.4
CYCPRED	48.3	15.8	04.6
Rituximab	48.1	14.2	04.6
Nintedanib	41.1	01.7	05.1
Pirfenidone	48.5	21.8	04.6
Pomalidomide	42.6	17.3	05.0

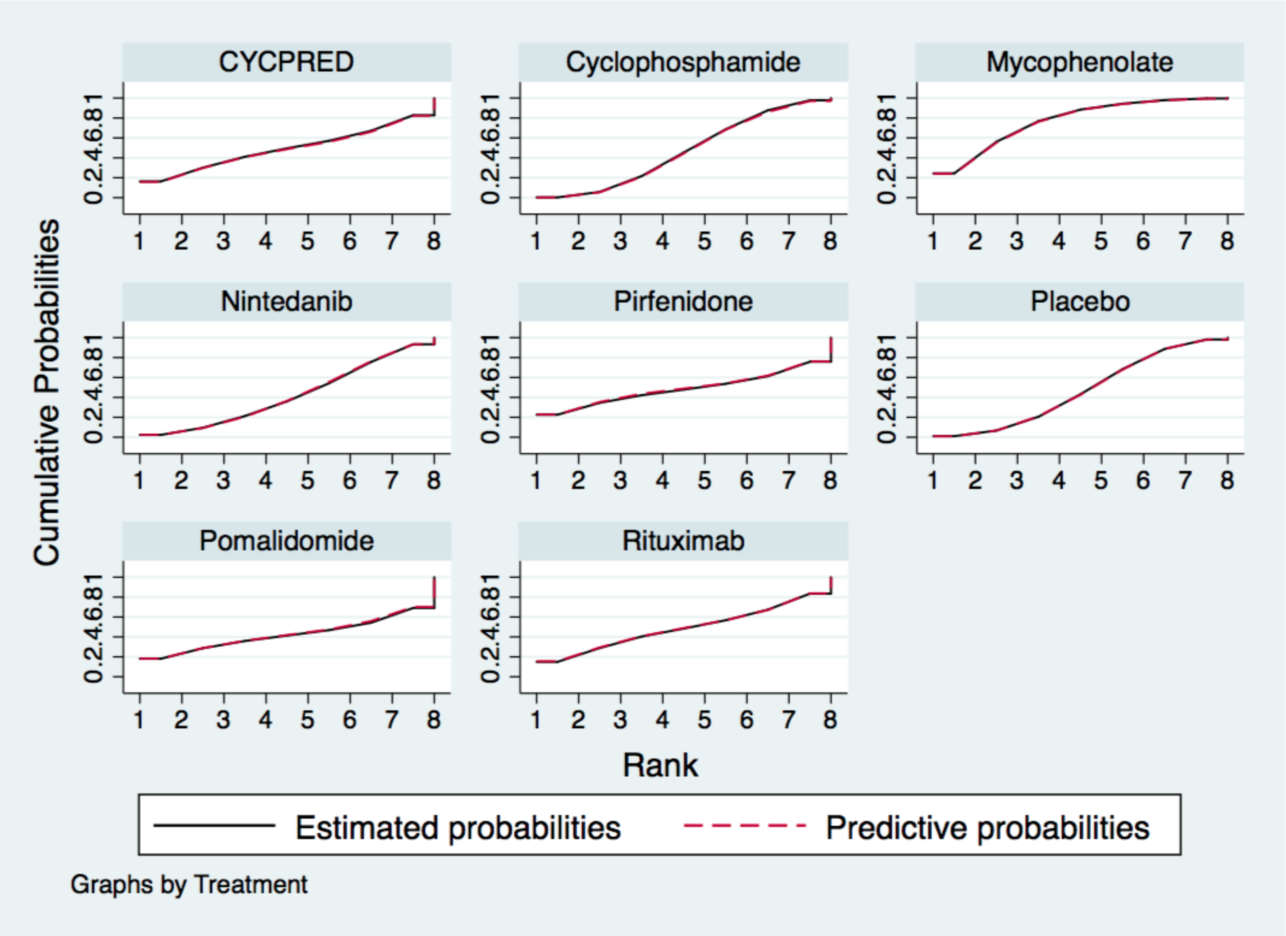

9. Sensitivity analysis

9.1 Effect estimates according to correlation factor

9.1.1 FVC % of predicted

	SMD (95% CI) Corr 0.8	SMD (95% CI) Corr 0.7	SMD (95% CI) Corr 0.5
Pomalidomide	-0.50(-1.43, 0.43)	-0.50(-1.43, 0.43)	-0.50(-1.43, 0.43)
Mycophenolate	-0.29(-0.69, 0.10)	-0.27(-0.66, 0.12)	-0.25(-0.54, 0.14)
Cyclophosphamide	0.08(-0.22, 0.38)	0.08(-0.22, 0.38)	0.07(-0.23, 0.37)
CYCPRED	0.23(-0.74, 1.20)	0.20(-0.76, 1.17)	0.16(-0.81, 1.14)
Pirfenidone	0.32(-0.36, 1.00)	0.33(-0.36, 1.00)	0.33(-0.32, 0.98)
CYCAZA	0.51(-0.14, 1.17)	0.42(-0.23, 1.07)	0.32(-0.36, 1.00)
Rituximab	1.00(0.39, 1.61)	0.83 (0.23, 1.44)	0.66(0.07, 1.26)

9.1.2 DLCO % of predicted

	SMD (95% CI) Corr 0.8	SMD (95% CI) Corr 0.7	SMD (95% CI) Corr 0.5
Cyclophosphamide	-0.08(-0.38, 0.21)	-0.08(-0.38, 0.23)	-0.07(-0.37, 0.23)
Nintedanib	-0.05(-0.21, -0.12)	-0.05(-0.21, 0.12)	-0.05(-0.21, 0.12)
CYCAZA	-0.01(-0.66, 0.63)	-0.01(-0.65, 0.63)	-0.01(-0.65, 0.64)
Mycophenolate	0.14(-0.25, 0.53)	0.12(-0.27, 0.50)	0.09(-0.30, 0.48)
CYCPRED	0.90(-0.44, 2.24)	0.75(-0.5, 2.06)	0.58(-0.69, 1.86)

9.2 Effect estimates according to the length of follow-up

9.2.1 FVC % of predicted

	SMD (95% CI) All studies	SMD (95% CI) Studies with 12-months of follow-up
Pomalidomide	-0.50(-1.43, 0.43)	-0.50(-1.43, 0.43)
Mycophenolate	-0.29(-0.69, 0.10)	0.00(-0.50, 0.49)
Cyclophosphamide	0.08(-0.22, 0.38)	0.21(-0.12, 0.53)
CYCPRED	0.23(-0.74, 1.20)	0.35(-0.63, 1.34)
Pirfenidone	0.32(-0.36, 1.00)	-
CYCAZA	0.51(-0.14, 1.17)	0.51(-0.14, 1.17)
Rituximab	1.00(0.39, 1.61)	-

9.2.2 DLCO % of predicted

	SMD (95% CI) All studies	SMD (95% CI) Studies with 12-months of follow-up
Cyclophosphamide	-0.08(-0.38, 0.21)	-0.08(-0.40, 0.25)
Nintedanib	-0.05(-0.21, -0.12)	-0.05(-0.21, 0.12)
CYCAZA	-0.01(-0.66, 0.63)	-0.01(-0.65, 0.63)
Mycophenolate	0.14(-0.25, 0.53)	0.16(-0.34, 0.66)
CYCPRED	0.90(-0.44, 2.24)	0.91(-0.44, 2.25)

10. Dataset

Paste on a Stata.dta the following dataset

study	study id	outcome	months	trt	Arm	n	mean	sd	events	rob
1	SLS-I, 2006	FVC	12	1	PBO	72	-2.60	7.6		1
1	SLS-I, 2006	FVC	12	2	CYC	73	-1.00	7.8		1
1	SLS-I, 2006	DLCO	12	1	PBO	72	-3.50	8.4		1
1	SLS-I, 2006	DLCO	12	2	CYC	73	-4.20	9.9		1
1	SLS-I, 2006	SAE	12	1	PBO	76			16	1
1	SLS-I, 2006	SAE	12	2	CYC	79			20	1
1	SLS-I, 2006	Deaths	12	1	PBO	76			6	1
1	SLS-I, 2006	Deaths	12	2	CYC	79			6	1
2	SLS-II, 2016	FVC	12	2	CYC	51	3.36	6.6		1
2	SLS-II, 2016	FVC	12	3	MMF	59	1.93	6.9		1
2	SLS-II, 2016	DLCO	12	2	CYC	51	-7.88	10.3		1
2	SLS-II, 2016	DLCO	12	3	MMF	58	-5.58	9.3		1
2	SLS-II, 2016	SAE	12	2	CYC	73			22	1
2	SLS-II, 2016	SAE	12	3	MMF	69			27	1
2	SLS-II, 2016	Withdrawals	12	2	CYC	73			15	1
2	SLS-II, 2016	Withdrawals	12	3	MMF	69			7	1
2	SLS-II, 2016	Deaths	12	2	CYC	73			11	1
2	SLS-II, 2016	Deaths	12	3	MMF	69			5	1
3	Domiciano DS, 2011	FVC	12	2	CYC	9	-2.11	10.7		3
3	Domiciano DS, 2011	FVC	12	4	CYCPRED	9	-0.77	5.8		3
3	Domiciano DS, 2011	DLCO	12	2	CYC	5	-14.60	9.1		3
3	Domiciano DS, 2011	DLCO	12	4	CYCPRED	6	-4.00	10.2		3
3	Domiciano DS, 2011	Deaths	12	2	CYC	9			1	3
3	Domiciano DS, 2011	Deaths	12	4	CYCPRED	9			1	3
4	Hoyles RK, 2006	FVC	12	1	PBO	18	-3.00	13.0		3
4	Hoyles RK, 2006	FVC	12	5	CYCAZA	19	2.40	6.8		3
4	Hoyles RK, 2006	DLCO	12	1	PBO	18	-3.20	8.9		3
4	Hoyles RK, 2006	DLCO	12	5	CYCAZA	19	-3.30	7.0		3
4	Hoyles RK, 2006	Withdrawals	12	1	PBO	23			0	3
4	Hoyles RK, 2006	Withdrawals	12	5	CYCAZA	22			2	3
5	Naidu GSRSNK, 2020	FVC	6	1	PBO	21	1.28	4.2		3
5	Naidu GSRSNK, 2020	FVC	6	3	MMF	20	-3.68	8.0		3
5	Naidu GSRSNK, 2020	DLCO	6	1	PBO	21	1.50	10.8		3
5	Naidu GSRSNK, 2020	DLCO	6	3	MMF	20	2.93	14.7		3
5	Naidu GSRSNK, 2020	SAE	6	1	PBO	21			0	3
5	Naidu GSRSNK, 2020	SAE	6	3	MMF	20			1	3
5	Naidu GSRSNK, 2020	Withdrawals	6	1	PBO	21			0	3
5	Naidu GSRSNK, 2020	Withdrawals	6	3	MMF	20			3	3
6	Sircar G, 2018	FVC	6	2	CYC	30	-1.19	7.8		3
6	Sircar G, 2018	FVC	6	6	RTX	30	6.22	8.1		3
6	Sircar G, 2018	Deaths	6	2	CYC	30			1	3
6	Sircar G, 2018	Deaths	6	6	RTX	30			1	3
7	SENSCIS, 2019	DLCO	12	1	PBO	288	-2.77	9.1		1
7	SENSCIS, 2019	DLCO	12	7	NTD	287	-3.21	9.1		1
7	SENSCIS, 2019	SAE	12	1	PBO	288			62	1
7	SENSCIS, 2019	SAE	12	7	NTD	287			69	1
7	SENSCIS, 2019	Withdrawals	12	1	PBO	288			25	1
7	SENSCIS, 2019	Withdrawals	12	7	NTD	287			46	1
7	SENSCIS, 2019	Deaths	12	1	PBO	288			9	1
7	SENSCIS, 2019	Deaths	12	7	NTD	287			10	1
8	Acharya N, 2019	FVC	6	1	PBO	17	-4.25	14.8		1
8	Acharya N, 2019	FVC	6	8	PFD	17	-0.69	4.4		1
8	Acharya N, 2019	Withdrawals	6	1	PBO	17			0	1
8	Acharya N, 2019	Withdrawals	6	8	PFD	17			3	1
8	Acharya N, 2019	Deaths	6	1	PBO	17			0	1
8	Acharya N, 2019	Deaths	6	8	PFD	17			0	1
9	Hsu VM, 2018	FVC	12	1	PBO	11	-2.80	4.0		3

9	Hsu VM, 2018	FVC	12	9	POMA	8	-5.20	5.3		3
9	Hsu VM, 2018	SAE	12	1	PBO	11			1	3
9	Hsu VM, 2018	SAE	12	9	POMA	8			4	3
9	Hsu VM, 2018	Withdrawals	12	1	PBO	11			0	3
9	Hsu VM, 2018	Withdrawals	12	9	POMA	8			4	3
9	Hsu VM, 2018	Deaths	12	1	PBO	11			0	3
9	Hsu VM, 2018	Deaths	12	9	POMA	8			0	3

11. Stata syntax

Paste the following commands into a Stata.do

```
use "/Users/user/Desktop/NMA.dta", clear
* OUTCOME SELECTION "Change FVC % of predicted"
keep if outcome=="FVC"
* NETWORK SETUP
network setup mean sd n, studyvar(study) trtvar(trt) armvars(drop) numcodes ref(1) smd
list study _*
network convert pairs
network table
gen invvarES=1/( _stderr^2)
list study _*
* NETWORK MAP
networkplot _t1 _t2, nodecolor(navy) labels(Placebo Cyclophosphamide Mycophenolate CYCPRED CYCAZA Rituximab
Pirfenidone Pomalidomide) edgecolor(by rob mean)
* NETWEIGHT
netweight _y _stderr _t1 _t2, scale(0.7) asp(0.7)
* CONSISTENCY and INCONSISTENCY testing
ifplot _y _stderr _t1 _t2 study, plotopt(texts(140)) xlabel(0, 5, 10) notab tau2(loop)
network convert augment
network meta c, fixed
network meta i, fixed
network sidesplit 1 2
network sidesplit 1 3
network sidesplit 3 2
* INTERVALPLOT
network meta c, fixed
intervalplot, null(0) reference(Placebo) labels(Placebo Cyclophosphamide Mycophenolate CYCPRED CYCAZA Rituximab
Pirfenidone Pomalidomide) margin(1 20 5 5)
* SUCRA
network meta c, fixed
network rank max, zero all reps(10000) gen(prob)
sucra prob*, labels(Placebo Cyclophosphamide Mycophenolate CYCPRED CYCAZA Rituximab Pirfenidone Pomalidomide)
network rank max, zero all reps(10000) gen(pred_prob) predict
sucra prob*, labels(Placebo Cyclophosphamide Mycophenolate CYCPRED CYCAZA Rituximab Pirfenidone Pomalidomide)
compare(pred_prob*) names("Estimated probabilities" "Predictive probabilities")
* NETLEAGUE
network meta c, fixed
netleague, labels(Placebo Cyclophosphamide Mycophenolate CYCPRED CYCAZA Rituximab Pirfenidone Pomalidomide)
sort(Placebo Cyclophosphamide Mycophenolate CYCPRED CYCAZA Rituximab Pirfenidone Pomalidomide)

use "/Users/user/Desktop/NMA.dta", clear
* OUTCOME SELECTION "Change DLCO % of predicted"
keep if outcome=="DLCO"
* NETWORK SETUP
network setup mean sd n, studyvar(study) trtvar(trt) armvars(drop) numcodes ref(1) smd
network convert pairs
network table
gen invvarES=1/( _stderr^2)
list study _*
* NETWORK MAP
networkplot _t1 _t2, labels(Placebo Cyclophosphamide Mycophenolate CYCPRED CYCAZA Nintedanib) edgecolor(by rob
mean)
* NETWEIGHT
netweight _y _stderr _t1 _t2, asp(0.7)
* CONSISTENCY and INCONSISTENCY testing
ifplot _y _stderr _t1 _t2 study, plotopt(texts(140)) xlabel(0, 5, 10, 15) notab tau2(loop)
network convert augment
network meta c, fixed
network meta i, fixed
network sidesplit 1 2
network sidesplit 1 3
network sidesplit 3 2
* INTERVALPLOT
network meta c, fixed
intervalplot, null(0) reference(Placebo) labels(Placebo Cyclophosphamide Mycophenolate CYCPRED CYCAZA Nintedanib)
margin(1 20 5 5)
* SUCRA
network meta c, fixed
network rank max, zero all reps(10000) gen(prob)
sucra prob*, labels(Placebo Cyclophosphamide Mycophenolate CYCPRED CYCAZA Nintedanib)
network rank max, zero all reps(10000) gen(pred_prob) predict
sucra prob*, labels(Placebo Cyclophosphamide Mycophenolate CYCPRED CYCAZA Nintedanib) compare(pred_prob*)
names("Estimated probabilities" "Predictive probabilities")
* NETLEAGUE
network meta c, fixed
netleague, labels(Placebo Cyclophosphamide Mycophenolate CYCPRED CYCAZA Nintedanib) sort(Placebo Cyclophosphamide
Mycophenolate CYCPRED CYCAZA Nintedanib)

use "/Users/user/Desktop/NMA.dta", clear
* OUTCOME SELECTION "Mumber of patients with serious adverse events"
keep if outcome=="SAE"
* NETWORK SETUP
network setup events n, studyvar(study) trtvar(trt) armvars(drop) numcodes ref(1) or
network convert pairs
network table
gen invvarES=1/( _stderr^2)
```

```
list study _*
* NETWORK MAP
networkplot _t1 _t2, labels(Placebo Cyclophosphamide Mycophenolate Nintedanib Pomalidomide) edgecolor(by rob mean)
* NETWEIGHT
netweight _y _stderr _t1 _t2, asp(0.7)
* CONSISTENCY and INCONSISTENCY testing
ifplot _y _stderr _t1 _t2 study, plotopt(texts(140)) xlabel(0, 2.5, 5) notab tau2(loop)
network convert augment
network meta c, fixed
network meta i, fixed
network sidesplit all
network sidesplit 1 2
network sidesplit 1 3
network sidesplit 3 2
* INTERVALPLOT
network meta c, fixed
intervalplot, null(1) reference(Placebo) labels(Placebo Cyclophosphamide Mycophenolate Nintedanib Pomalidomide)
margin(1 20 5 5)
* SUCRA
network meta c, fixed
network rank min, zero all reps(10000) gen(prob)
sucra prob*, labels(Placebo Cyclophosphamide Mycophenolate Nintedanib Pomalidomide)
network rank min, zero all reps(10000) gen(pred_prob) predict
sucra prob*, labels(Placebo Cyclophosphamide Mycophenolate Nintedanib Pomalidomide) compare(pred_prob*)
names("Estimated probabilities" "Predictive probabilities")
* NETLEAGUE
network meta c, fixed
netleague, labels(Placebo Cyclophosphamide Mycophenolate Nintedanib Pomalidomide) sort(Placebo Cyclophosphamide
Mycophenolate Nintedanib Pomalidomide)

use "/Users/user/Desktop/NMA.dta", clear
* OUTCOME SELECTION "Mumber of patients with serious adverse events"
keep if outcome=="Withdrawals"
* NETWORK SETUP
network setup events n, studyvar(study) trtvar(trt) armvars(drop) numcodes ref(1) or
network convert pairs
network table
gen invvarES=1/( _stderr^2)
list study _*
* NETWORK MAP
networkplot _t1 _t2, labels(Placebo Cyclophosphamide Mycophenolate CYCAZA Nintedanib Pirfenidone Pomalidomide)
edgecolor(by rob mean)
* NETWEIGHT
netweight _y _stderr _t1 _t2, asp(0.7)
* CONSISTENCY and INCONSISTENCY testing
ifplot _y _stderr _t1 _t2 study, plotopt(texts(140)) xlabel(0, 2.5, 5) notab tau2(loop)
network convert augment
network meta c, fixed
* INTERVALPLOT
network meta c, fixed
intervalplot, null(1) reference(Placebo) labels(Placebo Cyclophosphamide Mycophenolate CYCAZA Nintedanib
Pirfenidone Pomalidomide) margin(1 20 5 5)
* SUCRA
network meta c, fixed
network rank min, zero all reps(10000) gen(prob)
sucra prob*, labels(Placebo Cyclophosphamide Mycophenolate CYCAZA Nintedanib Pirfenidone Pomalidomide)
network rank min, zero all reps(10000) gen(pred_prob) predict
sucra prob*, labels(Placebo Cyclophosphamide Mycophenolate CYCAZA Nintedanib Pirfenidone Pomalidomide)
compare(pred_prob*) names("Estimated probabilities" "Predictive probabilities")
* NETLEAGUE
network meta c, fixed
netleague, labels(Placebo Cyclophosphamide Mycophenolate CYCAZA Nintedanib Pirfenidone Pomalidomide) sort(Placebo
Cyclophosphamide Mycophenolate CYCAZA Nintedanib Pirfenidone Pomalidomide)

use "/Users/user/Desktop/NMA.dta", clear
* OUTCOME SELECTION "Mumber of patients with serious adverse events"
keep if outcome=="Deaths"
* NETWORK SETUP
network setup events n, studyvar(study) trtvar(trt) armvars(drop) numcodes ref(1) or
network convert pairs
network table
gen invvarES=1/( _stderr^2)
list study _*
* NETWORK MAP
networkplot _t1 _t2, labels(Placebo Cyclophosphamide Mycophenolate CYCPRED Rituximab Nintedanib Pirfenidone
Pomalidomide) edgecolor(by rob mean)
* NETWEIGHT
netweight _y _stderr _t1 _t2, asp(0.7)
* CONSISTENCY and INCONSISTENCY testing
ifplot _y _stderr _t1 _t2 study, plotopt(texts(140)) xlabel(0, 2.5, 5) notab tau2(loop)
network convert augment
network meta c, fixed
* INTERVALPLOT
network meta c, fixed
intervalplot, null(1) reference(Placebo) labels(Placebo Cyclophosphamide Mycophenolate CYCPRED Rituximab
Nintedanib Pirfenidone Pomalidomide) margin(1 20 5 5)
* SUCRA
network meta c, fixed
network rank min, zero all reps(10000) gen(prob)
sucra prob*, labels(Placebo Cyclophosphamide Mycophenolate CYCPRED Rituximab Nintedanib Pirfenidone Pomalidomide)
```

```
network rank min, zero all reps(10000) gen(pred_prob) predict
sucra prob*, labels(Placebo Cyclophosphamide Mycophenolate CYCPRED Rituximab Nintedanib Pirfenidone Pomalidomide)
compare(pred_prob*) names("Estimated probabilities" "Predictive probabilities")
graph save SUCRA_SAE, replace
* NETLEAGUE
network meta c, fixed
netleague, labels(Placebo Cyclophosphamide Mycophenolate CYCPRED Rituximab Nintedanib Pirfenidone Pomalidomide)
sort(Placebo Cyclophosphamide Mycophenolate CYCPRED Rituximab Nintedanib Pirfenidone Pomalidomide)
```

12. Search strategy

EU Clinical Trials Registry EudraCT
"systemic sclerosis" NOT "multiple sclerosis", filter: adult, trials with results

ClinicalTrials.gov
lung (pulmonary, Pulmo), Systemic Sclerosis (Scleroderma, Diffuse sclerosis), Sclerosis (sclerose, Sclerotic); Studies with results; Interventional Studies.

Web of Science; All Databases (Web of Science Core Collection; Biological Abstracts; KCI, Korean Journal Database; MEDLINE®; Russian Science Citation Index: SciELO Citation Index).
#1 TS="systemic sclerosis"
#2 TS= scleroderma
#3 TS= "SSc-ILD"
#4 #3 OR #2 OR #1
#5 TS= "tuberous sclerosis"
#6 TS= "multiple sclerosis"
#7 #6 OR #5
#8 #4 NOT #7
#9 TI= randomized
#10 TI= trial
#11 TI= blind
#12 TI= randomly
#13 TI= placebo
#14 TI= randomised
#15 TI= versus
#16 TI= rituximab
#17 TI= cyclophosphamide
#18 TI= azathioprine
#19 TI= methotrexate
#20 TI= mycophenolate
#21 TI= belimumab
#22 TI= abatacept
#23 #22 OR #21 OR #20 OR #19 OR #18 OR #17 OR #16 OR #15 OR #14 OR #13 OR #12 OR #11 OR #10 OR #9
#24 #8 AND #23
#25 TI= review
#26 TI= retrospective
#27 TI= design
#28 TI= protocol
#29 #28 OR #27 OR #26 OR #25
#30 #24 NOT #29

Scopus
(((((KEY(systemic sclerosis)) OR (KEY(scleroderma)) OR (KEY(SSc-ILD))) AND NOT ((KEY(multiple sclerosis)) OR (KEY(tuberous sclerosis)))) AND ((KEY(pulmonary fibrosis)) OR (KEY(interstitial lung disease)) OR (KEY(pneumonia)) OR (KEY(lung)))) AND ((TITLE-ABS-KEY(randomized)) OR (TITLE-ABS-KEY(randomised)) OR (TITLE-ABS-KEY(trial)) OR (TITLE-ABS-KEY(controlled)) OR (TITLE-ABS-KEY(placebo)) OR (TITLE-ABS-KEY(versus)))) AND NOT ((TITLE-ABS-KEY(review)) OR (TITLE-ABS-KEY(open-label)) OR (TITLE-ABS-KEY(retrospective))) AND NOT INDEX(medline)
